# Genomic analyses of aminoacyl tRNA synthetases from human-infecting helminths

**DOI:** 10.1186/s12864-019-5679-0

**Published:** 2019-05-02

**Authors:** Preeti Goel, Suhel Parvez, Amit Sharma

**Affiliations:** 1 Structural Parasitology Group, International Centre for Genetic Engineering and Biotechnology (ICGEB), New Delhi, 110067 India; 2 Department of Toxicology, School of Chemical and Life Sciences, Jamia Hamdard, New Delhi, 110063 India

**Keywords:** Aminoacyl tRNA synthetases, Cladosporin, Halofuginone, Helminth, Inhibitors

## Abstract

**Background:**

Helminth infections affect ~ 60% of the human population that lives in tropical and subtropical regions worldwide. These infections result in diseases like schistosomiasis, lymphatic filariasis, river blindness and echinococcosis. Here we provide a comprehensive computational analysis of the aminoacyl tRNA synthetase (aaRS) enzyme family from 27 human-infecting helminths. Our analyses support the idea that several helminth aaRSs can be targeted for drug repurposing or for development of new drugs. For experimental validation, we focused on Onchocerciasis (also known as “river blindness”), a filarial vector-borne disease that is prevalent in Africa and Latin America. We show that halofuginone (HF) can act as a potent inhibitor of *Onchocerca volvulus* prolyl tRNA synthetase (*Ov*PRS).

**Results:**

The conserved enzyme family of aaRSs has been validated as druggable targets in numerous eukaryotic parasites. We thus embarked on assessing aaRSs from the genomes of 27 helminths that cause infections in humans. In order to delineate the distribution of aaRSs per genome we utilized Hidden Markov Models of aaRS catalytic domains to identify all orthologues. We note that *Fasciola hepatica* genome encodes the highest number of aaRS-like proteins (69) whereas *Taenia asiatica* has the lowest count (32). The number of genes for any particular aaRS-like protein varies from 1 to 8 in these 27 studied helminths. Sequence alignments of helminth-encoded lysyl, prolyl, leucyl and threonyl tRNA synthetases suggest that various known aaRS inhibitors like Cladosporin, Halofuginone, Benzoborale and Borrelidin may be of utility against helminths. The recombinantly expressed *Onchocerca volvulus* PRS was used as proof of concept for targeting aaRS with drug-like molecules like HF.

**Conclusions:**

Systematic analysis of unique subdomains within helminth aaRSs reveals the presence of a number of non-canonical domains like PAC3, Utp-14, Pex2_Pex12 fused to catalytic domains in the predicted helminth aaRSs. We have established a platform for biochemical validation of a large number of helminth aaRSs that can be targeted using available inhibitors to jump-start drug repurposing against human helminths.

**Electronic supplementary material:**

The online version of this article (10.1186/s12864-019-5679-0) contains supplementary material, which is available to authorized users.

## Background

Helminths are common infectious agents of humans in developing countries and cause significant mortality and morbidity [1]. Helminths are comprised of nematodes (roundworms- intestinal and filarial worms) as well as of platyhelminths (flatworms- flukes and schistosomes) [1]. Prevalence of soil transmitted helminth infections remains high in Sub-Saharan Africa, Latin America and Asia [2]. Helminth infections also contribute to physical disabilities, anemia, malnourishment and result in decreased productivity in the workplace [1, 3]. Some common diseases caused by helminths are river blindness, echinococcosis, dracunculiasis, taeniasis, schistosomiasis and lymphatic filariasis. Schistosomiasis alone infects ~ 230 million people in the developing world [4]. Both Hookworm and Schistosomiasis threaten pregnant women as these infections increase the risk of child and maternal morbidity [5]. Among people living in impoverished areas of developing countries, Onchocerciasis is prevalent and causes skin disease and visual impairment, while limb and genital deformities are the outcome of lymphatic filariasis [1]. Taeniasis caused by *Taenia solium* is known to cause blindness, headache, convulsions and epileptic seizures that can be fatal [6]. The threadworm *Strongyloides stercolaris* is found in digestive tracts where it can damage the duodenum and produce abdominal pain, epigastric tenderness and diarrhea [7]. Thus, helminth infections are a serious cause of poor health in many countries.

Despite the success of MDA (Mass Drug Administration) programmes, helminth diseases have not been eliminated in endemic areas and limitations have been observed in the effectiveness of treatments [1, 8, 9]. A list of known anthelmintics with their mechanism of action is provided in Table 1. There is concern of drug resistance to prime drugs including Benzimidazoles and Ivermectin [10–12]. Ivermectin is the drug of choice in helminth infections but is contraindicated in high-intensity *Loa loa* infection due to potential fatal side-effects [13, 14]. For soil-transmitted helminth infections, the most widely used drugs Albendazole and Mebendazole have shortcomings in their efficacy profiles, especially against infections by hookworm and *T. trichiura* [15, 16]. Hence, there is a need for the discovery of new druggable targets and inhibitor scaffolds in the context of human helminth infections.

**Table 1:**
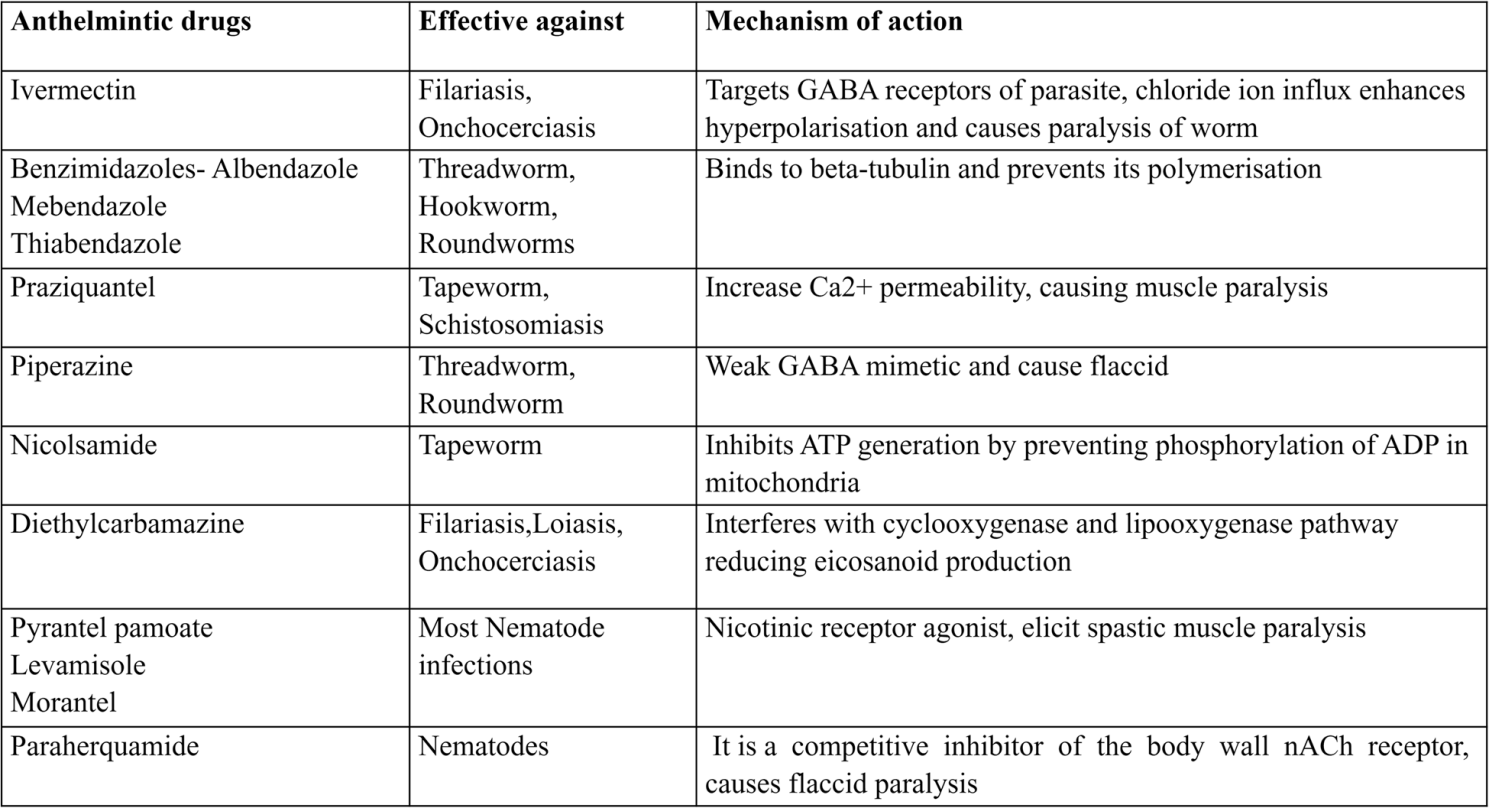
Major anthelmintic drugs and their mechanisms of action

Protein synthesis machinery is an important focus of drug targeting against many human pathogens. The aaRSs constitute a family of enzymes that are responsible for transfer of amino acid to their cognate tRNA molecules, a step integral to synthesis of proteins. The aaRSs are an important component of protein translational machinery in all cells, and have been validated as druggable targets. Few examples of aaRSs inhibitors include Cladosporin (CLD), Halofuginone (HF), Borrelidin and Benzoborale AN2690 (5-fluoro-1, 3-dihydro-1-hydroxy-2, 1-benzoxaborole) [17]. CLD competitively inhibits malaria parasite lysyl tRNA synthetase (KRS). It mimics ribose and adenine moieties of ATP as it has a THP ring (2, 6-disubstituted tetrahydropyran) fused to an iso-coumarin moiety [18]. HF has a quinazolinone ring fused to hydroxypiperidine ring and these respectively mimic the tRNA adenine and L-proline. HF is a potential inhibitor of prolyl tRNA synthetase of *Plasmodium falciparum* [19]. Borrelidin is known to inhibit threonyl tRNA synthetase (TRS). Borrelidin is an 18-membered macrolide-polyketide which simultaneously binds to four distinct pockets of ATP, amino acid, tRNA binding site and a fourth accessory site [20]. Benzoborale AN2690 (5-fluoro-1, 3-dihydro-1-hydroxy-2, 1-benzoxaborole) is known to block leucyl tRNA synthetase by covalently trapping tRNA in the editing site of the enzyme [21]. Apart from their translational functions, aaRSs are associated with various non-canonical functions such as role in angiogenesis, apoptosis, inflammation and immune responses [22]. Paralogs of aaRSs exist that have lost their aminoacylation functions and have adapted other roles [22, 23]. Hence our computational work lays the foundation for experimental dissection of helminth aaRSs and for understanding roles of aaRSs in parasite biology.

## Results

### Phylogenetic analyses of human-infecting helminth

**Fig. 1:**
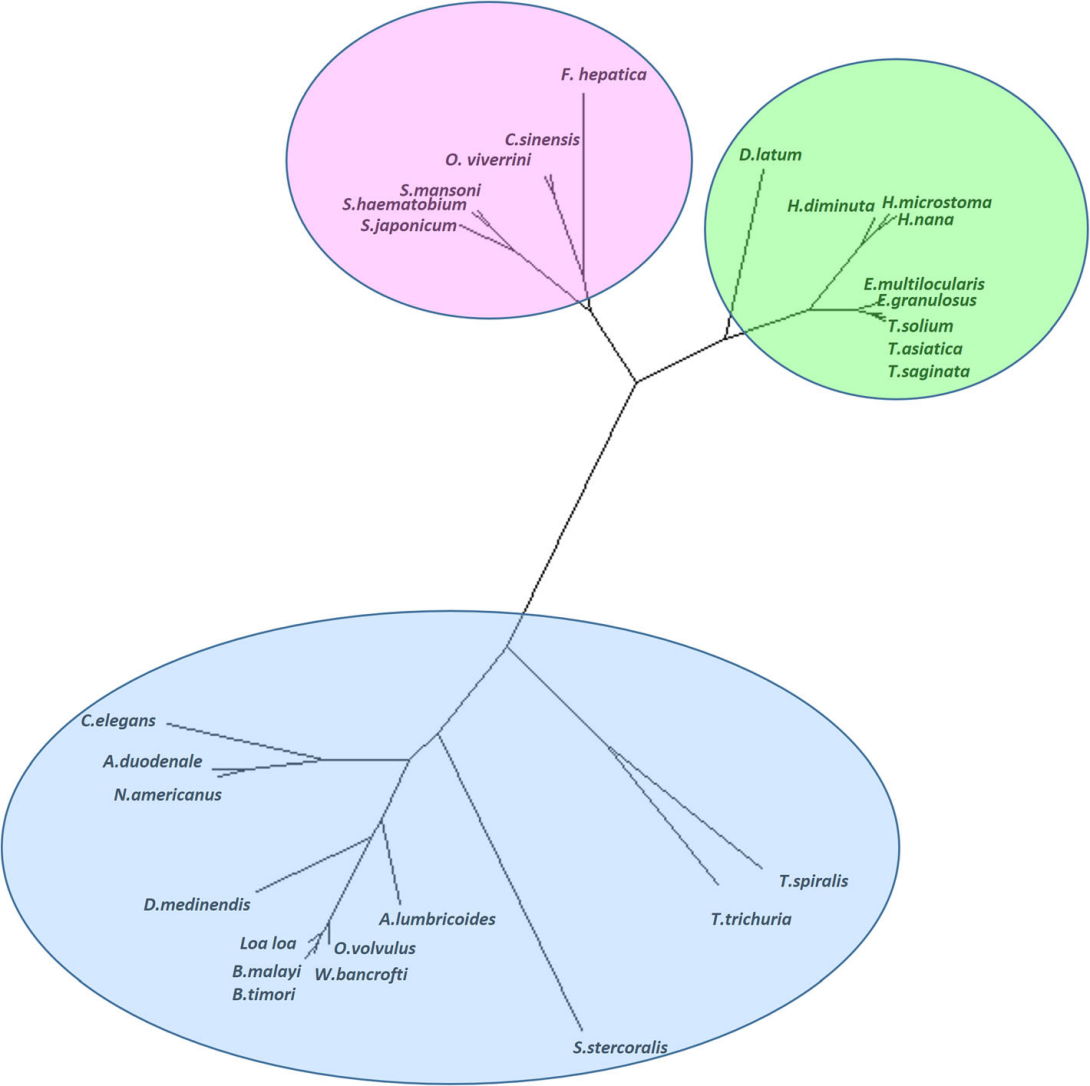
Phylogenetic tree of human infecting helminths based on their proteomes. Pink circle shows Trematodes, green shows Cestodes and the blue shows Nematodes

Humans are parasitized by two major groups of parasitic worms - the Nematoda (roundworms) and Platyhelminthes (flatworms). Platyhelminthes are part of the superphylum Lophotrochozoa whereas Nematoda is part of the superphylum Ecdysozoa - these two phylas are not related zoologically. Trematoda and Cestoda are likely monophyletic and have different pre-turbellarian ancestors. Trematoda (endoparasitic flukes) and Cestoda (endoparasitic tapeworms) have evolved in different directions each having unique morphology and life style [24, 25]. We constructed phylogenetic tree of the 27 human-infecting helminths based on their whole proteomes (Fig. 1). The tree displays groups of helminths as platyhelminths and nemathelminths.

The nematodes (round worms) are major intestinal worms and platyhelminths include cestodes (tapeworms) and trematodes (blood, tissue and organ flukes).

Phylogenetic analyses reveal close evolutionary linkage between various human-infecting helminths. However, helminths belonging to the same phylogenetic group (trematodes, cestodes and nematodes) do not cause similar human infections. Therefore above three groups are not divided based on types of infection caused or prevalence in any particular region.

To study the evolutionary aspects of aminoacyl tRNA synthetases present in these 27 helminths, as an example, we studied the evolutionary linkages of the two PRSs in *Onchocerca volvulus* of its total of 39 aaRSs. All *Onchocerca volvulus* aaRSs were evaluated for the presence of signal peptides and predictions suggest that 9 out of the 39 are likely to be mitochondrial. We surmised that of the two PRSs in *Onchocerca volvulus* one is likely cytoplasmic. We subjected both PRSs to phylogenetic analyses in order to determine their evolutionary relatedness to bacterial and eukaryotic aaRSs [26]. Interestingly, one OvPRS gene (OVOC 1179) clustered with bacterial PRSs and with mitochondrial PRSs in eukaryotes whereas the other OvPRS (OVOC 11842) clustered with cytoplasmic eukaryotic PRSs indicating different evolutionary origins for the two *Onchocerca volvulus* PRSs (Fig. 7). This phylogenetic analysis thus can be extended to annotate the evolutionary relatedness of all helminth aaRSs with each other. It is thus likely that the cytoplasmic and mitochondrial versions of aaRSs in these helminth have eukaryotic-like and prokaroytic-like lineages.

### Aminoacyl tRNA synthetase-like proteins in helminths

Aminoacyl tRNA synthetases are important enzymes of protein translation machinery and are also involved in various non canonical functions. It has been fruitful to compile genome-wide data on the distribution of aaRSs in various pathogens [27–29]. Comparative analyses of aaRSs genes has revealed that numerous genomes contain open reading frames that encode synthetase-like proteins that have a catalytic domain which is specific for a given tRNA synthetase [27]. Amongst these aaRS-like proteins, many are smaller than the corresponding functional tRNA synthetases [17]. It seems that evolution has recruited this family of enzymes into a range of diverse non-canonical, non aminoacylation functions [17]. Using similar ideas, we catalogued all aaRS-like proteins in the 27 human infecting helminths. The proteins with tRNA synthetase catalytic domains were identified based on HMM-profile searches, and then categorized into specific aaRS-like proteins based on blast searches against a database of well-annotated aaRSs (AARS database). Predominantly, an aaRS consists of a catalytic domain (which is involved in aminoacylation) and an anticodon binding domain (which binds to the anticodon region of the tRNA and ensures binding of tRNA to the correct amino acid). Some aaRSs have editing domains that cleave incorrectly paired amino acyl-tRNA molecules and few additional non canonical domains are also present in some tRNA synthetases [29, 30].

**Fig. 2:**
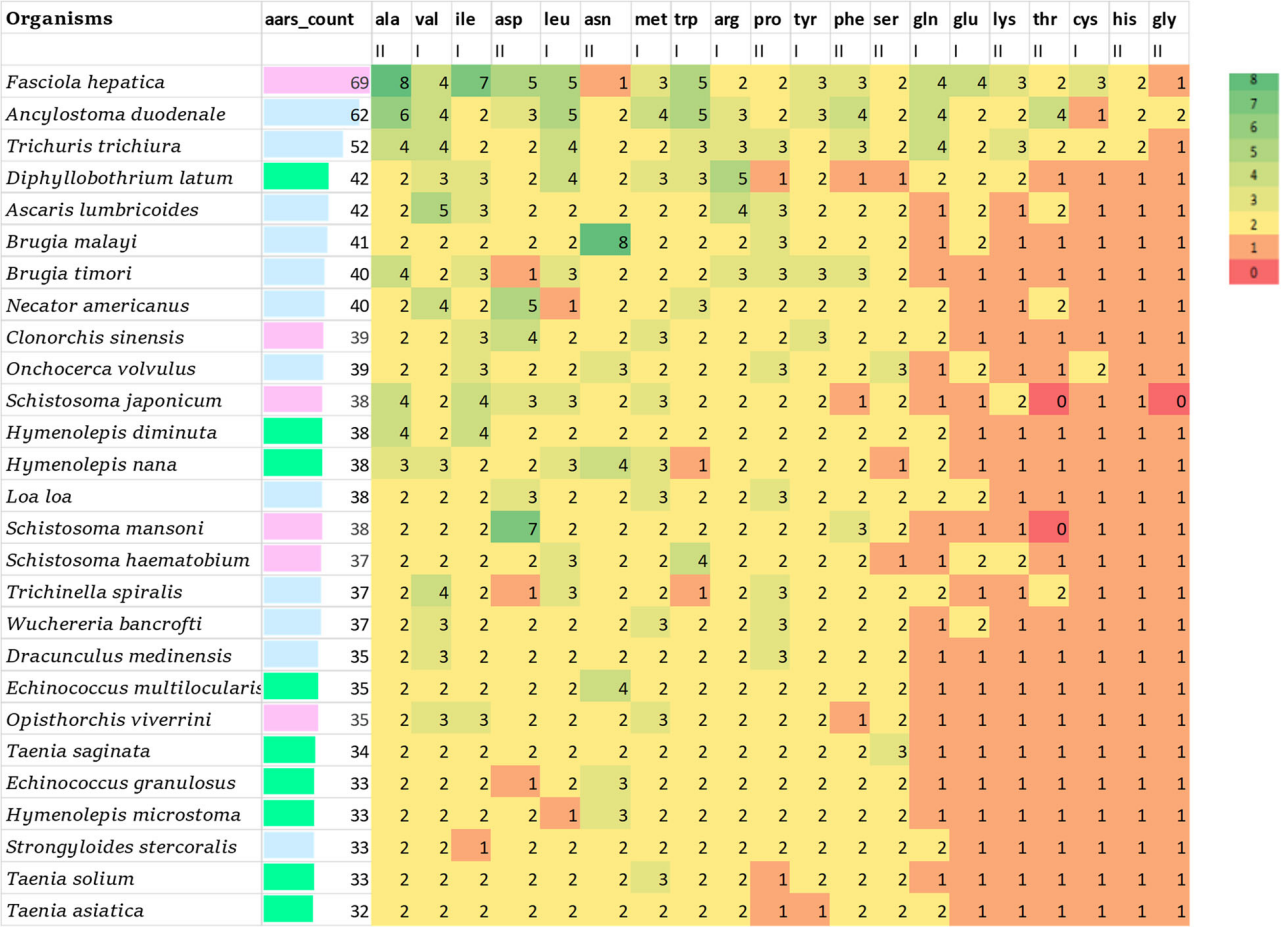
The number of aaRS-like proteins in human infecting helminths. The proteins are in descending order from left to right based on their number in each helminth. The green to orange is descending aaRS-like protein number for each amino acid in all 27 human infecting helminths. Green colour shows the presence of exceptionally high number of particular aaRSs (8–3), Yellow colour shows the presence of optimal number of particular aaRSs (2), Orange and red colour shows low number of particular aaRSs (1–0). The pink bars shows Trematodes, green shows Cestodes and the blue shows Nematodes

**Fig. 3:**
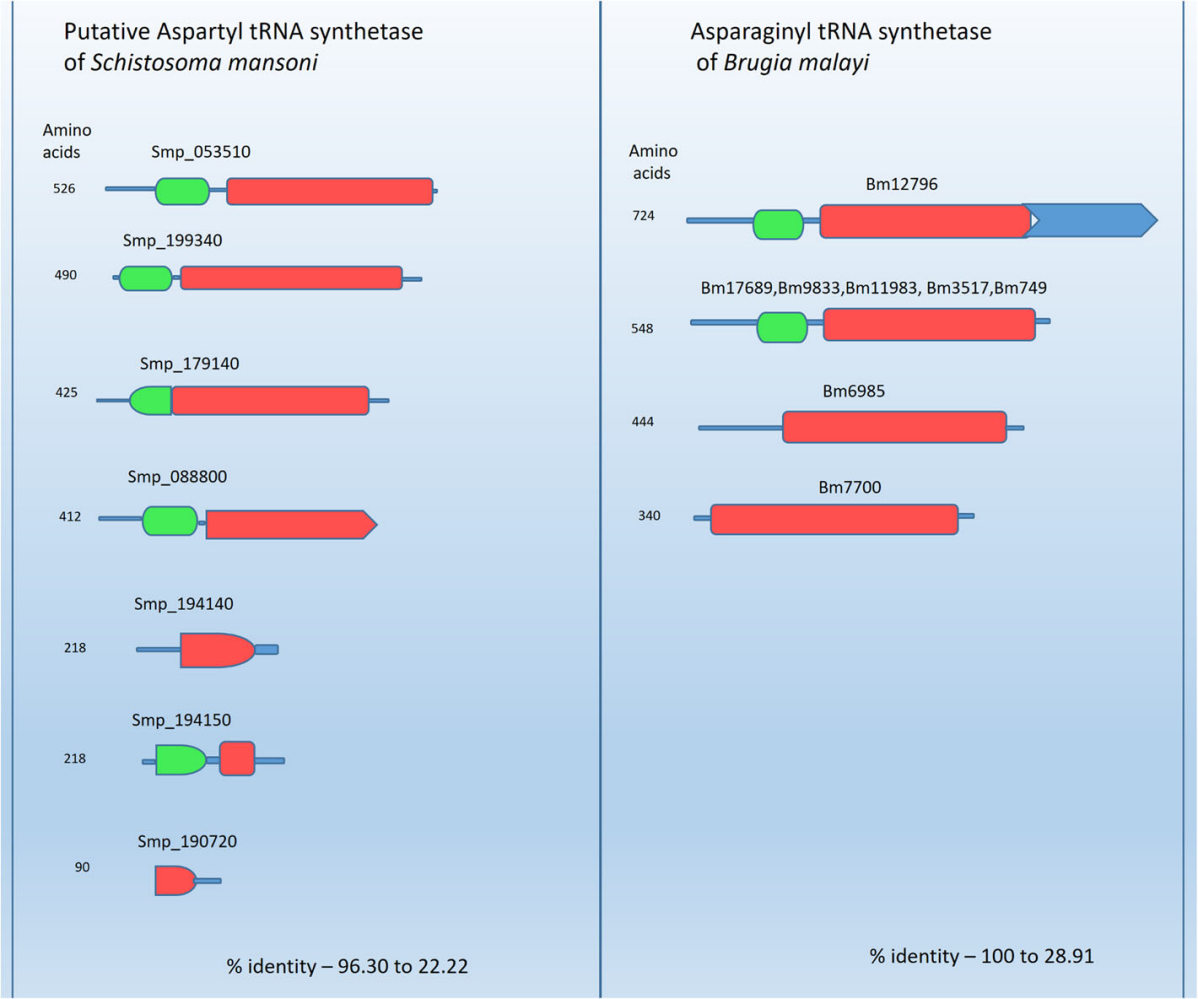
Domain diagrams of the predicted aspartyl tRNA synthetases in *Schistosoma mansoni* and asparaginyl tRNA synthetases in *Brugia malayi*. Red color denotes catalytic domain and green shows the anticodon-binding domain. The percent sequence identity in each of these groups is shown at bottom indicating that many of the redundant proteins are substantially different in their primary sequence

Interestingly different numbers of aaRS-like proteins are predicted in the 27 helminth genomes (Fig. 2 and Additional file 1: Table S1). For example, there are 69 aaRS-like proteins in *Fasciola hepatica* and this may be related to its large genome size. For regular protein synthesis, the expectation would be of 20 aaRSs each for cytoplasmic and mitochondrial translation, however, this does not hold true for many of the helminths (Fig. 2). We also observed multiple aaRS-like proteins for any particular amino acid in several helminth genomes - for example 8 NRSs in *Brugia malayi* and 7 DRSs in *Schistosoma mansoni* (Fig. 3). In *Brugia malayi*, a 5 gene cluster of NRS (IDs: Bm17689, Bm9833, Bm11983, Bm3517 and Bm749) is present on chromosome 3 (Fig. 3). These 5 genes encode for cytoplasmic NRS in this organism and this protein also serves as an antigenic determinant in *Brugia malayi* [31]. The cytoplasmic NRSs in *Brugia malayi* are secreted from the helminth and these NRS genes encode an immunodominant antigen which is responsible for strong antibody response in humans with lymphatic filariasis [31]. The atomic structure of *B*. *malayi* NRS suggests two structured domains: (1) an amino-terminal domain in which 80 amino acids fold as for interleukin-8 (IL-8) and interacts with the extracellular loops of G-protein coupled IL-8 receptors and (2) a 438 amino acid catalytic domain [32]. It has been shown that this *Bm*NRS promotes pro-inflammatory activities such as chemotaxis of cells that express IL-8 receptors [33]. The *Brugia malayi* NRS is an example of aaRSs that are present in high copy number and that likely perform additional non-canonical functions.

In *Schistosoma mansoni*, 7 DRS-like proteins were found and each of the respective genes vary in sequence and chromosomal location (Fig. 3). Transcriptome data indicates expression of 4 of 7 DRS-like genes in *Schistosoma mansoni* [34]. However, it remains unclear if all of these genes encode functional aaRSs although they encode catalytic domains of different molecular sizes (Fig. 3). In general, we found that several aaRS-like proteins such as alanyl, aspartyl, asparaginyl and isoleucyl aaRSs are present in multiple copies within the helminth genomes whereas threonyl, cysteinyl, glycyl and histidyl aaRSs are present as a single copy (Fig. 2). Apart from the presence of an unusually large number of aaRs-like proteins (total of 38) in *Schistosoma mansoni*, we were intrigued to note that no discernible TRS was identified in our searches. However, in literature two putative threonyl tRNA synthetases have been reported in *Schistosoma mansoni* [35], but we note that neither of these have the canonical catalytic domain of a tRNA synthetase. Additionally, our searches failed to find TRS and GRS in *Schistosoma japonicum.*

**Fig. 4:**
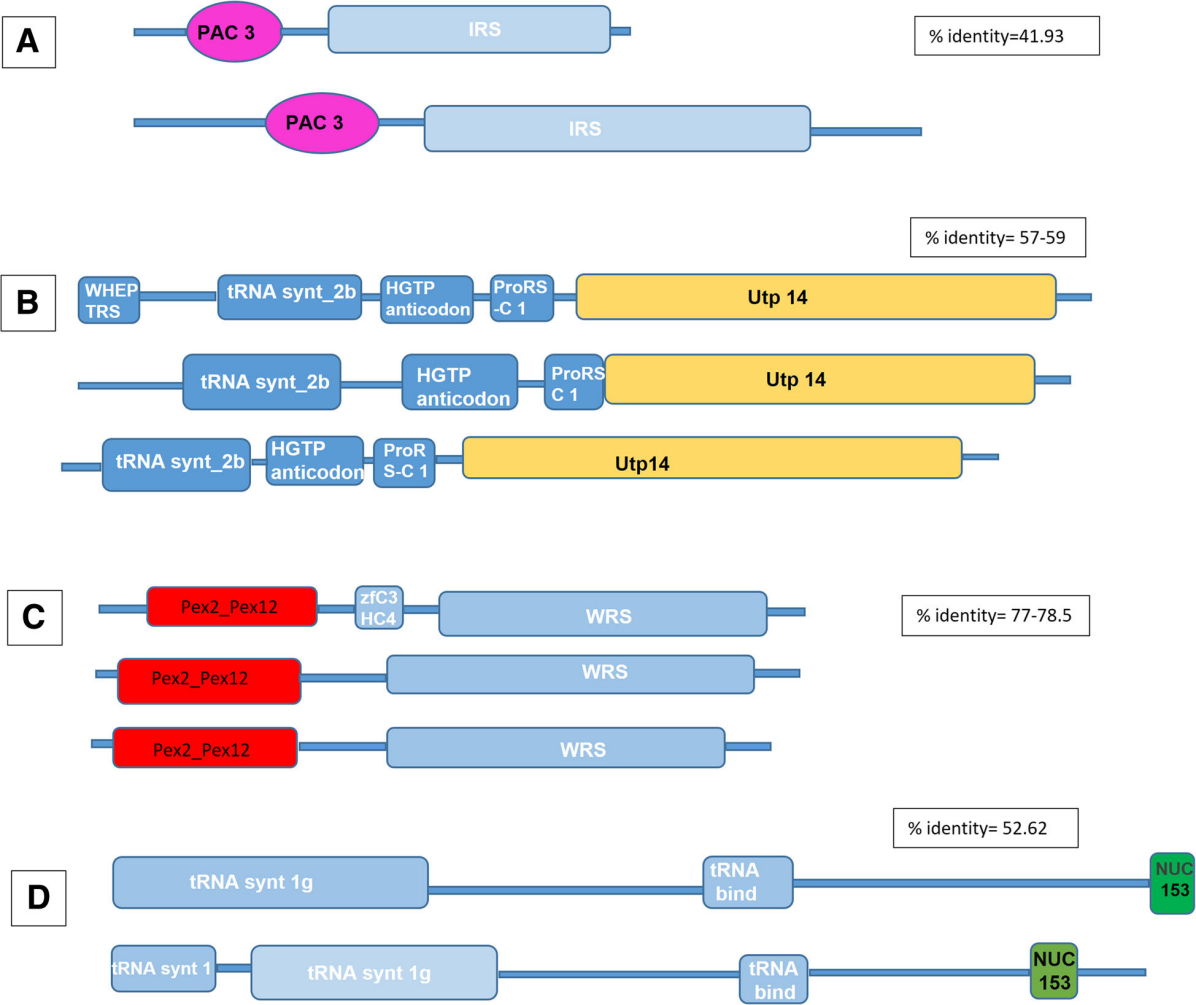
Distribution of various unusual domain fusions in aaRS-like proteins of human infecting helminths. **a**) PAC3 domain fused to IRS of *Brugia malayi* and *Necator americanus*, **b**) Utp14 domain fused to Bifunctional glutamate proline of *Ascaris lumbricoides*, *Loa loa* and *Dracunculas medinensis*, **c**) Pex2_Pex12 domain fused to WRS of *Brugia malayi*, *Loa loa* and *Onchocerca volvulus*, **d**) NUC 153 domain fused to methionyl tRNA synthetase of *Ascaris lumbricoides* and *Necator americanus*

We also found numerous non-canonical domains fused with the catalytic domains in helminth aaRS-like proteins (Additional file 2: Table S2). Two interesting examples are: (1) presence of PAC3 (proteasome assembly chaperone 3 proteins) domain in IRSs from *Brugia malayi* and *Necator americanus,* (2) presence of Utp-14 (part of a large ribonucleoprotein protein complex containing the U3 snoRNA) in PRSs from *Ascaris lumbricoides*, *Loa loa* and *Dracunculus medinensis* (Fig. 4). A complete list of such unusual domain fusions with helminth aaRSs is provided in Additional file 2: Table S2.

### Sequence and structural similarities in aaRS-like proteins

**Fig. 5:**
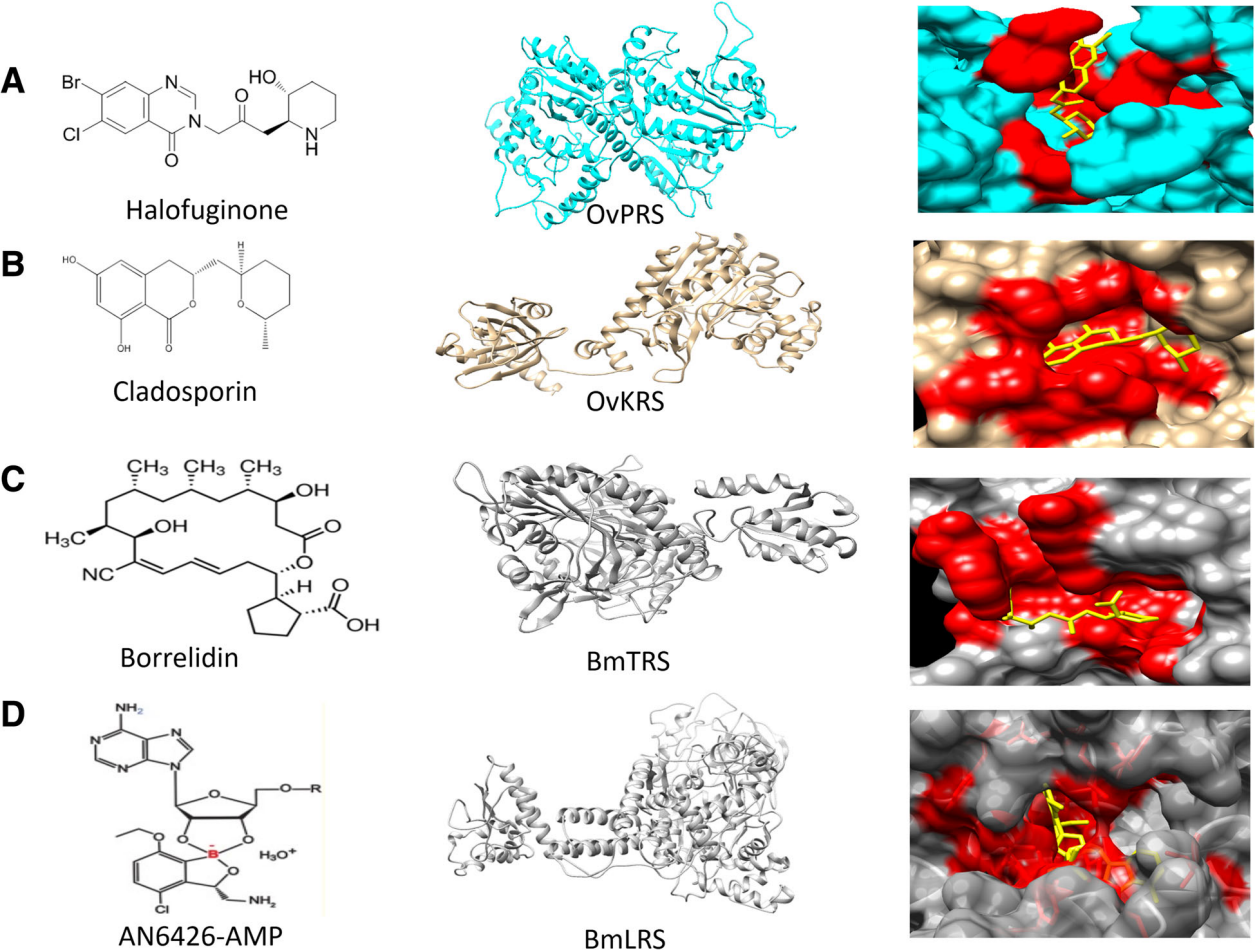
Modelling of drug-bound structures of tRNA synthetases from two helminths. The enzyme active sites are shown in red and the drug molecules are shown in yellow. **a**) Halofuginone bound to *Onchocerca volvulus* prolyl tRNA synthetase, **b**) Cladosporin bound to *Onchocerca volvulus* lysyl tRNA synthetase, **c**) Borrelidin bound to threonyl tRNA synthetase of *Brugia malayi*, **d**) Benzoborale bound to leucyl tRNA synthetase of *Brugia malayi*

We next decided to focus on specific tRNA synthetases in an effort to study their druggable properties. Analysis of lysyl tRNA synthetase from *Onchocerca volvulus* suggests the presence of only one copy of lysyl tRNA synthetase (KRS, gene ID- Ov2569) in it. We assessed via sequence alignments the possible structural similarities between *Onchocerca volvulus* KRS and *Plasmodium falciparum* KRS where the drug Cladosporin binds (Fig. 5b) [18, 35, 36]. We noted considerable sequence identity between *Ov/Pf* KRS cladosporin interacting residues (PDB ID: 4PG3). Similar analysis was done for *Brugia malayi* TRS (gene ID - Bm13920) using the complex *E. coli* TRS with the drug Borrelidin (PDB ID 4P3O). There are two LRSs predicted in *Brugia malayi* (Bm5408 and Bm7489). Sequence alignments with LRS of *Cryptosporidium muris* (PDB ID 5FOM*)* show conservation of benzoborale binding residues in one of the predicted LRS of *Brugia malayi* (Bm5408)*.* There are three predicted PRSs (Gene IDs Bm11475, Bm6054, Bm5875) in *Brugia malayi* and in other helminths like *Onchocerca volvulus*. Sequence alignments of these with *Pf*PRS show conservation in HF interacting residues for many helminths. These sequence and structural comparisons suggest that known inhibitors of tRNA synthetases may be of utility as starting points of drug discovery against helminths. Assessment of sequence conservation within inhibitor-binding residues in enzyme-inhibitor complexes can be used to identify potential lead compounds and their molecular targets in multiple helminths (Fig. 5). We then decided to validate these observations by recombinantly producing the *Ov*PRS.

### Activity and inhibition by halofuginone

**Fig. 6:**
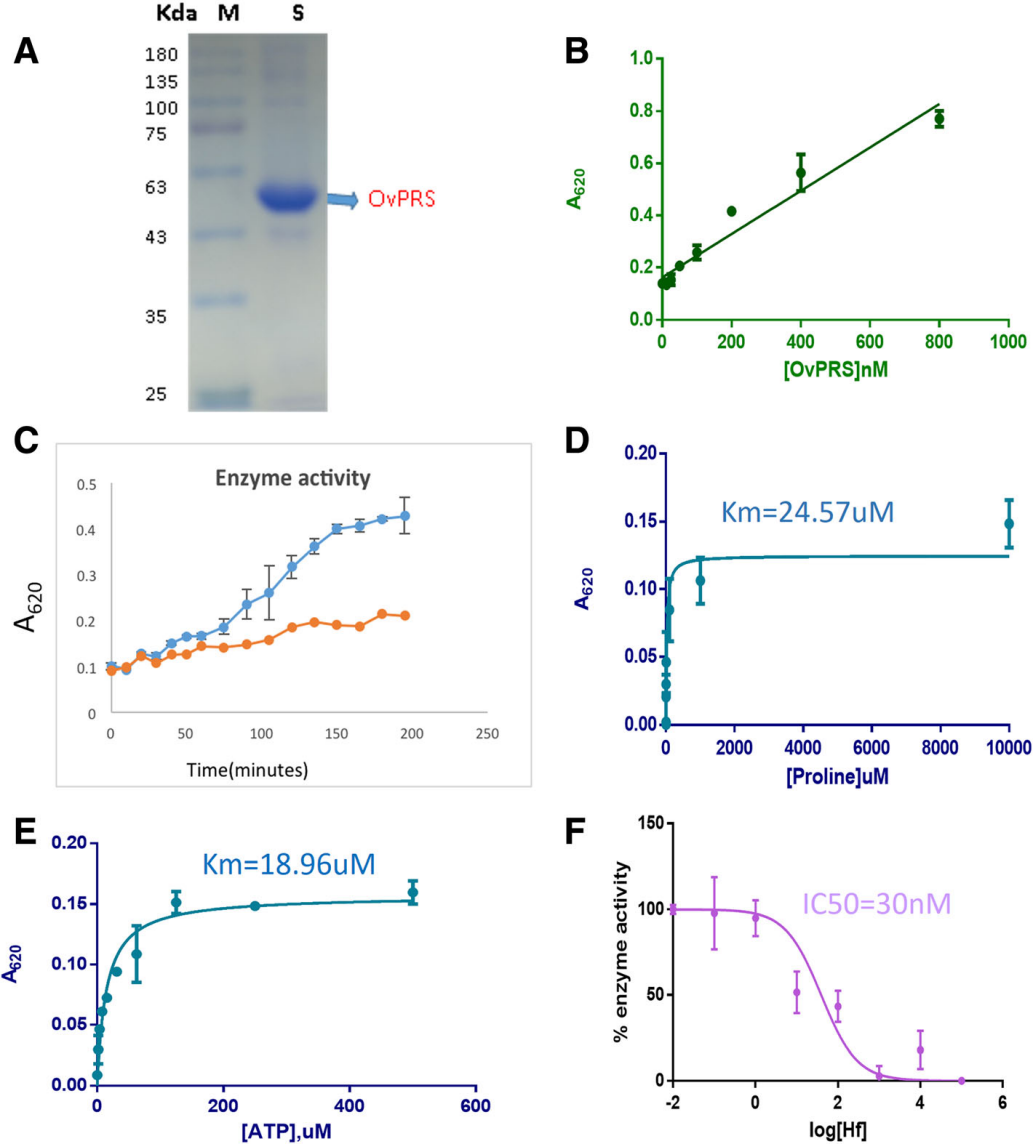
Enzymatic assays of *Onchocerca volvulus* PRS. **a**) Purified *Ov*PRS on SDS-PAGE **b**) Aminoacylation assay using malachite green dye (53). Linearity of the assay with respect to enzyme concentration. **c**) Assay linearity with respect to time, red shows control without enzyme and the blue is reaction in presence of enzyme. **d**, **e**) Proline Km determination in the presence of a saturating concentration of ATP (200 μM), ATP Km determination in the presence of saturating concentration of proline (1 mM). **f**) Enzyme inhibition with halofuginone using 50 μM proline, 150 μM ATP and 150 nM *Ov*PRS enzyme. The IC50 of halofuginone for inhibition of *Ov*PRS is estimated to be ~ 30 nM

Halofuginone is a known inhibitor of *Plasmodium falciparum* PRS [19]. HF was tested against *Onchocerca volvulus* PRS based on the binding site sequence similarities between *Pf*PRS and *Ov*PRS. Assay conditions were optimized to determine the activity of *Ov*PRS in absence and presence of HF (Fig.6). This involved determining the optimal enzyme concentration, the linearity of assay with respect to time, and the Michaelis Menten constants for proline and ATP (Fig. 6 a-e). The apparent *K*_m_ for proline and ATP were determined to be 24.17 ± 11 μM and 18.9 ± 2.2 μM. Interestingly, HF showed an IC50 value of ~ 30 nM against *Ov*PRS (Fig. 6f). These results thus highlight the utility of HF against *Ov*PRS as also potentially against other helminth PRSs.

## Discussion

Treatment and prevention of infections caused by helminths presents an emerging challenge. There is an imminent need to strengthen efforts to identify new anti-parasitic agents with alternative modes of action against helminth diseases. Dissecting and understanding the critical components of helminth protein production machinery will help establish a platform for targeting specific helminth aaRSs. Data from several laboratories over the past decade has shown that the aaRS family of enzymes from eukaryotic parasites are druggable [18, 19, 37–42]. The human/pathogen aaRSs can at times show minor/major differences in their active site residues or additional residues thereby conferring selectivity [36]. The idea of STOPP (Structure-based targeting of orthologous pathogen proteins) has been implemented for pathogen aaRSs [41]. Two examples of STOPP are (1) halofuginone in context of PRSs from *Plasmodium falciparum*, *Toxoplasma gondii*, *Cryptosporidium parvum*, *Leishmania major* and *Eimeria tenella* [43], and (2) cladosporin in context of KRSs of *Plasmodium falciparum*, *Loa loa* and *Schistosoma mansoni* [35].

In this work, we have studied the distribution of aaRS-like proteins in 27 human infecting helminths. The number of any particular aminoacyl tRNA synthetase varies from 1 to 8 in the studied group of helminths. Ideally there should be only two copies (one cytoplasmic and one mitochondrial) for a particular aaRS is each helminth but this does not hold true for most of the 27 studied organisms as shown in Fig.2.

We have modelled four aminoacyl tRNA synthetases from *Brugia malayi* and *Onchocerca volvulus* in order to investigate whether these helminth enzymes may be druggable just as their homologs are [19, 37–39]. The currently known anti-aaRSs compounds can be utilised to target these four helminth aaRSs and to jumpstart drug repurposing. We analysed multiple sequence alignments to assess the binding sites within aaRSs (PRS, KRS, LRS and TRS) for the following inhibitors: halofuginone, cladosporin, benzoborale and borellidin in the 27 studied human-infecting helminths (Additional file 3: Figure S1). Several helminth aaRSs display high conservation in the above binding residues and indeed modelling presented here shows the feasibility of drug targeting.

## Conclusions

There is an immediate need to find new targets and drugs for helminth infections due to the potential of evolving drug resistance. The novelty of this study lies in computational analysis that combines Hidden Markov Models (HMM) and similarity searches against tRNA synthetase catalytic domains to identify all aaRSs in 27 human-infecting helminths. This is the first report of an extensive analysis of helminth aaRSs, and sets up avenues for experimental validation of our observations. If pursued, helminth aaRSs may prove to be of utility as novel targets for anti-helminth drug development.

## Methods

### Data collection and computational analysis

**Fig. 7:**
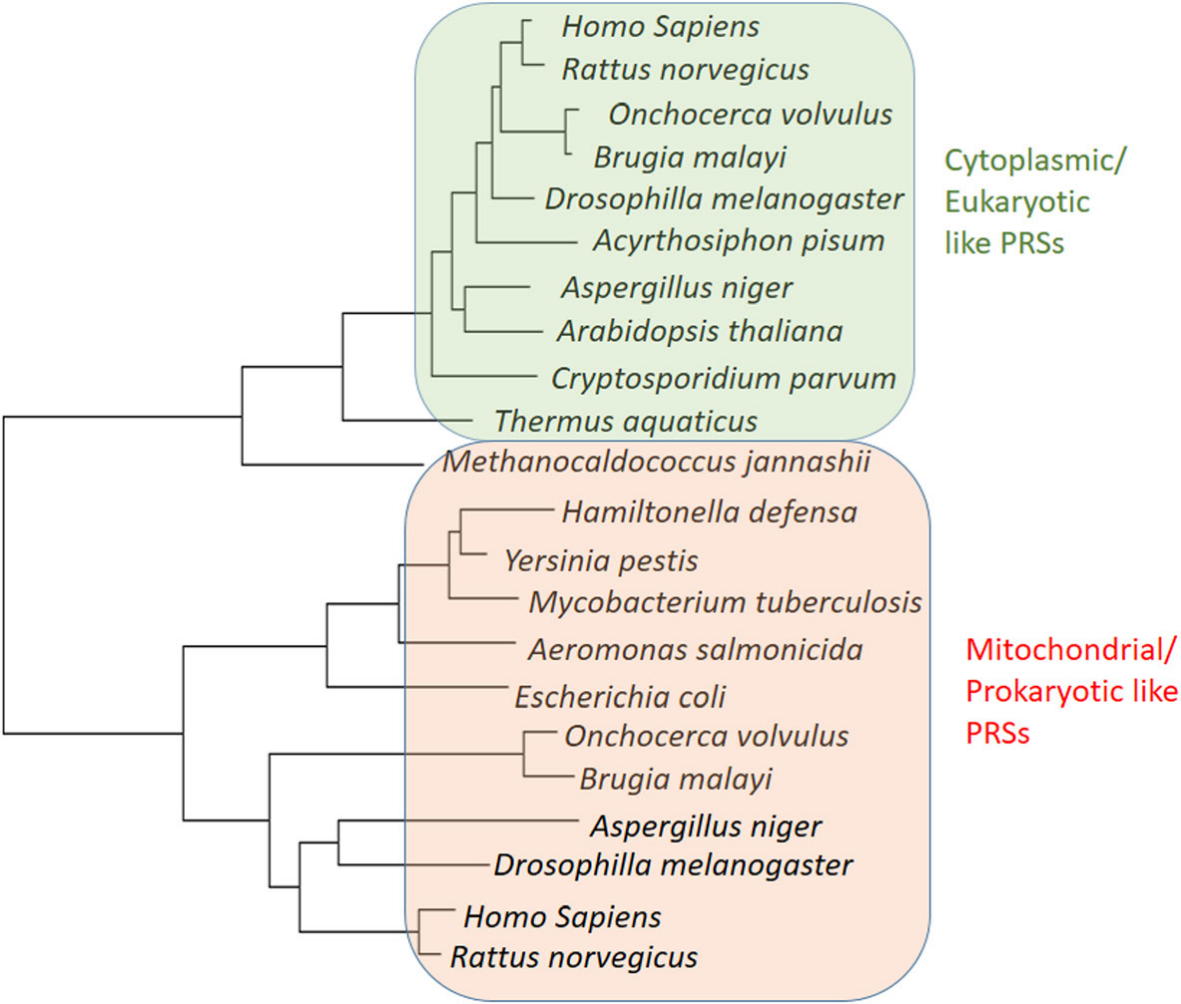
Phylogenetic analysis of the two (cytoplasmic and mitochondrial) prolyl tRNA synthetases of *Onchocerca Volvulus*

Whole proteome FASTA files for human-infecting helminths were downloaded from WormBase parasite database [44]. HMMER software package and HMMs for catalytic domains of tRNA synthetases were downloaded from pfam database [45]. To identify potential aaRSs, hmm searches were carried out using tRNA synthetases catalytic domains (tRNA-synt_1b, tRNA-synt_1f, tRNA-synt_2, tRNA-synt_2b, tRNA-synt_2c, tRNA-synt_1e, tRNA-synt_1g, tRNA-synt_2d, tRNA-synt_1d, tRNA-synt_1, tRNA-synt_2e, tRNA-synt_1c) with gathering threshold. This threshold is the default setting for hmmscan which instructs HMMER to use the sequence and hit thresholds defined in the searched HMM files. These values are set conservatively to ensure that there are no known false positives. These searches identified 1071 hits in 27 helminth genomes. The identified hits were annotated to specific tRNA synthetase by using BLAST searches against annotated tRNA synthetases downloaded from AARSs database using e-value of 1e^− 6^ [46]. Total of 1070 (Additional file 1: Table S1) of 1071 hits identified from hmm searches also gave at least 10 significant hits in the BLAST searches against annotated tRNA synthetase from AARS DataBase. Any given tRNA synthetase was annotated to specific amino acid if majority of the top 10 significant BLAST hits were for particular amino acid. Non-canonical domains in aaRSs were identified by analyzing hmm searches of all domains in Pfam-A database using gathering threshold. Orthologs of human infecting helminths were identified using the best bidirectional hit algorithm using BLASTP with 1e^− 6^ as the e-value cutoff [47]. There were 687 core genes in these 27 genomes with orthologs which were then aligned using clustalO [48]. The alignments were cleaned using GBlocks with default parameters to remove non-informative and gapped sites [49]. A maximum likelihood phylogenetic tree was constructed using RAxML with 100 bootstraps [50, 51]. The substitution model was automatically determined by RAxML. The tree was visualized using dendroscope [52]. Sequences of the two *Ov*PRSs (OVOC1179 AND OVOC11842) were retrieved from WormBase parasite database and a BLAST search was carried against them. The resulting sequences from different organisms were then aligned using Clustalw and a phylogenetic tree was constructed in MEGA7 [53]. The evolutionary history was inferred by using Maximum Likelihood method based on the JTT matrix-based model. The tree with highest log likelihood (− 6518.26) is shown in (Fig. 7) Initial tree (s) for the heuristic searches were obtained automatically by applying Neighbour-Join and BioNJ algorithms to a matrix of pairwise distances estimated using a JTT model, we then selected the topology with superior log likelihood value. The tree is drawn to scale, with branch lengths measured in the number of substitutions per site. All positions containing gaps and missing data were eliminated. The analysis involved 22 amino acid sequences. Evolutionary analyses were conducted in MEGA7.

Transcriptome data for *Schistosoma mansoni* were obtained from Lu Z et al. [34] Protein sequences of aaRSs (lysyl, leucyl, prolyl, and threonyl) and their structural information were downloaded from databases. A multiple alignment study was performed on lysyl, leucyl, prolyl and threonyl tRNA synthetases of human helminths using clustal Omega. Drug binding residues for each tRNA synthetase were identified from structure and literature. The structures of leucyl and threonyl tRNA synthetases of *Brugia malayi* were modelled and compared with the structure of already known leucyl tRNA synthetase of *Cryptosporidium muris* (PDB ID 5FOM*)* and threonyl tRNA synthetase of *E.coli* respectively (PDB ID4P3O). Lysyl and prolyl tRNA synthetase of *Onchocerca volvulus* were modelled and compared to lysyl and prolyl tRNA synthetases from *Plasmodium falciparum* (PDB IDs: 4YCV, 4YDQ).

### Protein expression and purification of *Ov*PRS

The *Ov*PRS sequence was retrieved from the WormBase database. We made a smaller construct of full-length *Ov*PRS on the basis of structural modelling. The construct was designed for optimal expression in *E. coli*. The *Ov*PRS construct consists of 497 amino acids (279–775; 57 kDa). This construct was sub-cloned in pETM-41 (maltose binding protein with histidine tag). Recombinant *Ov*PRS was produced in *E. coli* BL21 codon+ cells. Protein expression was induced by adding 0.5 mM isopropyl b-D-thiogalactopyranoside (IPTG) to cells grown at 37 °C for 4 h, and for 20 h post-induction at 18 °C. Cells were obtained by centrifugation at 5000 g for 15 min. Bacterial cell pellet was resuspended in a buffer containing 50 mM Tris–HCl pH 8.0, 500 mM NaCl, 10 mM bME, 15% *v*/v glycerol, 0.1 mg ml^-1^ lysozyme and EDTA free protease inhibitor cocktail (Roche). Cells were lysed by sonication and cleared by centrifugation at 13,000 g for 45 min. The supernatant was applied to amylose beads (NEB) and protein was eluted using the buffer (50 mM Tris–HCl pH 8.0, 250 mM NaCl, 10 mM bME,10 mM maltose). TEV protease was added and kept for 24 h at 20 °C to remove the tag. Cleaved *Ov*PRS protein was purified by gel filtration chromatography on Superdex 200 column 16/60 GL (GE Healthcare) which was equilibrated with 20 mM Tris-HCl pH 8, 250 mM NaCl and 2 mM DTT. Eluted *Ov*PRS fractions were observed on SDS-PAGE and the pure ones were pooled, concentrated to 15 mg/ml and stored at − 80 °C.

### Aminoacylation assays

The aminoacylation reactions (100 μl total volume each) were performed in clear, flat-bottomed, 96-well plates (Costar 96-well standard microplates), and the reaction mixtures were incubated for 1 h at 37 °C. In the first step of amino acylation reaction pyrophosphate is released which is converted to inorganic phosphate by PPiase. This liberated inorganic phosphate was detected by addition of 25 μl of malachite green solution and incubated for 10 min at room temperature. Malachite green is a dye that detects the PPi formed in the reaction which correlates with enzyme activity [54]. This method is based on the formation of a complex between malachite green molybdate and free inorganic phosphates that absorbs at 620–640 nm. Absorbance was measured at 620 nm using a spectrophotometer. Reactions without enzyme or without L-Pro were performed as background controls, and data from reactions without enzyme were subtracted from the measurements. 10 mM EDTA was added to the reaction mixture at different time points (5, 10, 20, 30, 40–180 min) and mixed on ice to stop the reaction in order to observe the dependence of enzyme activity on time. For *Ov*PRS inhibition, a reaction solution containing PRS (0.15 μM) was mixed with HF at 100uM-0.1 nM (10 fold dilutions).

## Additional files

Additional file 1: **Table S1.** A list of all aaRSs like proteins from 27 studied human infecting helminths along with their gene IDs. (PDF 97 kb)

Additional file 2: **Table S2.** Domain description of all the aaRSs like proteins showing the presence of unusual domains. Unusual domains are mentioned in red. (PDF 99 kb)

Additional file 3: **Figure S1.** Multiple sequence alignment (MSA) of aaRSs 1) PRS of *Plasmodium falciparum* with PRSs of all studied human-infecting helminths showing conservation in HF binding residues, 2) CLD binding residues in *Plasmodium falciparum* aligned with KRSs of helminths, 3) binding residues in TRS of *Cryptosporidium muris* aligned with TRSs of helminths, 4) Benzoborale binding residues in LRS of *E.coli* aligned with LRSs of helminths. (DOCX 1888 kb)

## Abbreviations

aaRSs: Aminoacyl tRNA synthetases
AMP: Adenosine mono phosphate
ATP: Adenosine tri phosphate
CLD: Cladosporin
DRS: Aspartyl tRNA synthetase
HF: Halofuginone
HMM: Hidden markov model
IC50: Inhibitory concentration 50
IL-8: Interleukin-8
Km: Michaelis Menten constant
KRS: Lysyl tRNA synthetase
LRS: Leucyl tRNA synthetase
MDA: Mass Drug Administration
NRS: Asparaginyl tRNA synthetase
*Ov*PRS: *Onchocerca volvulus* prolyl tRNA synthetase
PAC3: Proteasome assembly chaperone 3
THP: 2,6-disubstituted tetrahydropyran
TRS: Threonyl tRNA synthetase
