# Supplementary material for: Genomic analyses of aminoacyl tRNA synthetases from human-infecting helminths
Source: BMC Genomics. 2019 May 2;20:333. doi: 10.1186/s12864-019-5679-0 (PMC6498573; doi:10.1186/s12864-019-5679-0)
Supplement: Supplementary file 1 — Table S1. A list of all aaRSs like proteins from 27 studied human infecting helminths along with their gene IDs. (PDF 97 kb) [file 12864_2019_5679_MOESM1_ESM.pdf]

Ancyclostoma duodenale	aaRSs
ANCDUO_12845	Leucyl
ANCDUO_05963	Arginyl
ANCDUO_18591	Arginyl
ANCDUO_08798	Valyl
ANCDUO_25895	Alanyl
ANCDUO_09533	Alanyl
ANCDUO_17345	Alanyl
ANCDUO_05820	Asparaginyl
ANCDUO_13630	Lysyl
ANCDUO_26463	Alanyl
ANCDUO_20791	Threonyl
ANCDUO_19811	PhenylAlanyl
ANCDUO_02067	Cysteinyl
ANCDUO_18955	Aspartyl
ANCDUO_24055	Tryptophanyl
ANCDUO_24911	Prolyl
ANCDUO_18234	Glycyl
ANCDUO_19768	PhenylAlanyl
ANCDUO_19068	Histidyl
ANCDUO_27496	Tyrosyl
ANCDUO_15714	Seryl
ANCDUO_00820	Methionyl
ANCDUO_25110	Valyl
ANCDUO_11265	Tryptophanyl
ANCDUO_18661	Glutaminyl
ANCDUO_09186	Histidyl
ANCDUO_00405	Aspartyl
ANCDUO_16790	Lysyl
ANCDUO_03641	Glutamyl
ANCDUO_18953	Aspartyl
ANCDUO_09370	Tyrosyl
ANCDUO_24027	Alanyl
ANCDUO_24251	Leucyl
ANCDUO_03331	Arginyl
ANCDUO_07419	threonyl
ANCDUO_11081	Alanyl
ANCDUO_15560	Prolyl
ANCDUO_10383	Isoleucyl
ANCDUO_14858	threonyl
ANCDUO_18662	Glutamyl
ANCDUO_08616	Glutaminyl
ANCDUO_06892	Valyl
ANCDUO_11709	Leucyl
ANCDUO_06455	seryl
ANCDUO_23171	Phenylalaninyl
ANCDUO_12569	Tryptophanyl
ANCDUO_04992	Valyl
ANCDUO_07292	Methionyl
ANCDUO_13732	Asparaginyl
ANCDUO_11991	PhenylAlanyl
ANCDUO_25109	Leucyl

ANCDUO_26559	Glycyl
ANCDUO_07362	Tryptophanyl
ANCDUO_16028	Glutaminyl
ANCDUO_18271	threonyl
ANCDUO_18384	Tyrosyl
ANCDUO_24372	Methionyl
ANCDUO_12570	Tryptophanyl
ANCDUO_26984	Methionyl
ANCDUO_08542	Leucyl
ANCDUO_01345	Isoleucyl
ANCDUO_23741	Glutaminyl

Ascaris lumbricoides	aaRSs
ALUE_0000267201-mRNA-1	Glutamyl
ALUE_0000277801-mRNA-1	Histidyl
ALUE_0000337301-mRNA-1	Methionyl
ALUE_0000379701-mRNA-1	Glutaminyl
ALUE_0000385201-mRNA-1	Prolyl
ALUE_0000390001-mRNA-1	Alanyl
ALUE_0000390801-mRNA-1	Prolyl
ALUE_0000401801-mRNA-1	Leucyl
ALUE_0000463601-mRNA-1	Cysteinyl
ALUE_0000483701-mRNA-1	Prolyl
ALUE_0000498601-mRNA-1	Leucyl
ALUE_0000645401-mRNA-1	Lysyl
ALUE_0000681201-mRNA-1	Isoleucyl
ALUE_0000774701-mRNA-1	Glycyl
ALUE_0000822901-mRNA-1	Phenylalanyl
ALUE_0000865301-mRNA-1	Glutamyl
ALUE_0000883001-mRNA-1	Asparaginyl
ALUE_0000911501-mRNA-1	Valyl
ALUE_0001038001-mRNA-1	Valyl
ALUE_0001048001-mRNA-1	Aspartyl
ALUE_0001133801-mRNA-1	Tyrosyl
ALUE_0001210501-mRNA-1	Phenylalanyl
ALUE_0001228401-mRNA-1	Aspartyl
ALUE_0001289101-mRNA-1	Seryl
ALUE_0001307801-mRNA-1	Seryl
ALUE_0001367301-mRNA-1	Tyrosyl
ALUE_0001373501-mRNA-1	Tryptophanyl
ALUE_0001389901-mRNA-1	Isoleucyl
ALUE_0001586901-mRNA-1	Tryptophanyl
ALUE_0001615801-mRNA-1	Arginyl
ALUE_0001633301-mRNA-1	threonyl
ALUE_0001678701-mRNA-1	Asparaginyl
ALUE_0001753901-mRNA-1	Methionyl
ALUE_0001831201-mRNA-1	Alanyl
ALUE_0002027701-mRNA-1	Valyl
ALUE_0002066301-mRNA-1	Arginyl
ALUE_0002084501-mRNA-1	Valyl
ALUE_0002131801-mRNA-1	Arginyl
ALUE_0002174301-mRNA-1	Arginyl

ALUE_0002260301-mRNA-1	threonyl
ALUE_0002293401-mRNA-1	Valyl
ALUE_0002358501-mRNA-1	Isoleucyl
Brugia malayi	aaRSs
Bm10312a	Phenylalanyl
Bm11475a	Prolyl
Bm11983	Asparaginyl
Bm12638	Cysteinyl
Bm12796	Asparaginyl
Bm13678a	Alanyl
Bm13726	Isoleucyl
Bm13741	Aspartyl
Bm13816	Glutaminyl
Bm13878	Threonyl
Bm13920a	Lysyl
Bm13923a	Histidyl
Bm13950	Seryl
Bm13961a	Tyrosyl
Bm13983	PhenylAlanyl
Bm14742	Glutamyl
Bm15491	Aspartyl
Bm17689	Asparaginyl
Bm2357a	Isoleucyl
Bm2473	Arginyl
Bm2816	Seryl
Bm347	Glutamyl
Bm3517a	Asparaginyl
Bm3629a	Arginyl
Bm4545	Methionyl
Bm5408	Leucyl
Bm5713a	Glycyl
Bm5875	Prolyl
Bm6054	Prolyl
Bm6985	Asparaginyl
Bm7157	Tryptophanyl
Bm7171a	Valyl
Bm7239	Tyrosyl
Bm724	Tryptophanyl
Bm7240	Methionyl
Bm7405	Valyl
Bm7489a	Leucyl
Bm749	Asparaginyl
Bm7700	Asparaginyl
Bm9427a	Alanyl
Bm9833a	Asparaginyl
Brugia timori	aaRSs
BTMF_0000071201-mRNA-1	Valyl
BTMF_0000108701-mRNA-1	Tyrosyl
BTMF_0000142801-mRNA-1	Glutaminyl
BTMF_0000197801-mRNA-1	Alanyl

BTMF_0000272701-mRNA-1	Glutamyl
BTMF_0000351101-mRNA-1	Tyrosyl
BTMF_0000383501-mRNA-1	Isoleucyl
BTMF_0000436001-mRNA-1	Leucyl
BTMF_0000481701-mRNA-1	Seryl
BTMF_0000547201-mRNA-1	Phenylalanyl
BTMF_0000586501-mRNA-1	Arginyl
BTMF_0000791701-mRNA-1	Histidyl
BTMF_0000795301-mRNA-1	Alanyl
BTMF_0000826501-mRNA-1	Methionyl
BTMF_0000836201-mRNA-1	Leucyl
BTMF_0000840201-mRNA-1	Asparaginyl
BTMF_0000852201-mRNA-1	Prolyl
BTMF_0000859301-mRNA-1	Methionyl
BTMF_0000877601-mRNA-1	Arginyl
BTMF_0000882501-mRNA-1	Lysyl
BTMF_0000917001-mRNA-1	Arginyl
BTMF_0000924901-mRNA-1	Glycyl
BTMF_0000961301-mRNA-1	Isoleucyl
BTMF_0000965501-mRNA-1	Asparaginyl
BTMF_0000980601-mRNA-1	Alanyl
BTMF_0000994401-mRNA-1	Tryptophanyl
BTMF_0001103301-mRNA-1	Isoleucyl
BTMF_0001212101-mRNA-1	Valyl
BTMF_0001213901-mRNA-1	Cysteinyl
BTMF_0001214301-mRNA-1	Tryptophanyl
BTMF_0001222401-mRNA-1	Aspartyl
BTMF_0001243101-mRNA-1	Seryl
BTMF_0001326601-mRNA-1	Threonyl
BTMF_0001334901-mRNA-1	PhenylAlanyl
BTMF_0001416801-mRNA-1	PhenylAlanyl
BTMF_0001555901-mRNA-1	Prolyl
BTMF_0001599001-mRNA-1	Alanyl
BTMF_0001635901-mRNA-1	Leucyl
BTMF_0001656901-mRNA-1	Tyrosyl
BTMF_0001821501-mRNA-1	Prolyl

Colonorchis sinensis	aaRSs
csin103748	Glutamyl
csin107926	Alanyl
csin104824	Methionyl
csin113533	Prolyl
csin111222	Glutaminyl
csin113588	Tyrosyl
csin107343	Isoleucyl
csin102069	Isoleucyl
csin100230	Tyrosyl
csin109131	Lysyl
csin108881	Histidyl
csin103683	Alanyl
csin101029	Arginyl
csin107533	Asparaginyl

csin100392	Threonyl
csin111567	Glycyl
csin108055	Seryl
csin100225	Asparaginyl
csin106767	Cysteinyl
csin113592	Tyrosyl
csin106629	Aspartyl
csin100211	Arginyl
csin105292	Valyl
csin109169	PhenylAlanyl
csin108609	Leucyl
csin105286	Tryptophanyl
csin103200	Valyl
csin109806	Aspartyl
csin112601	Methionyl
csin103939	Tryptophanyl
csin113526	Aspartyl
csin111916	Seryl
csin100096	Leucyl
csin101886	Aspartyl
csin112273	Prolyl
csin109224	Glutaminyl
csin113519	Isoleucyl
csin109978	Methionyl
csin112583	Phenylalanyl

Diphyllobothrium latum	aaRSs
DILT_0000003901-mRNA-1	Valyl
DILT_0000330901-mRNA-1	Valyl
DILT_0000355801-mRNA-1	Prolyl
DILT_0000401801-mRNA-1	Alanyl
DILT_0000408101-mRNA-1	Glutamyl
DILT_0000510201-mRNA-1	Glycyl
DILT_0000519501-mRNA-1	Valyl
DILT_0000546901-mRNA-1	Histidyl
DILT_0000585301-mRNA-1	Aspartyl
DILT_0000621201-mRNA-1	Alanyl
DILT_0000721001-mRNA-1	Phenylalanyl
DILT_0000731901-mRNA-1	Methionyl
DILT_0000786001-mRNA-1	Isoleucyl
DILT_0000798901-mRNA-1	Isoleucyl
DILT_0000823801-mRNA-1	Aspartyl
DILT_0000824101-mRNA-1	Arginyl
DILT_0000894601-mRNA-1	Cysteinyl
DILT_0000909001-mRNA-1	Tryptophanyl
DILT_0000913801-mRNA-1	Tyrosyl
DILT_0000915901-mRNA-1	Arginyl
DILT_0000976601-mRNA-1	Lysyl
DILT_0001210401-mRNA-1	Glutaminyl
DILT_0001228301-mRNA-1	Leucyl
DILT_0001260701-mRNA-1	Leucyl
DILT_0001262101-mRNA-1	Leucyl

DILT_0001288401-mRNA-1	Asparaginyl
DILT_0001355001-mRNA-1	Arginyl
DILT_0001380301-mRNA-1	Methionyl
DILT_0001412401-mRNA-1	Arginyl
DILT_0001415301-mRNA-1	Methionyl
DILT_0001438101-mRNA-1	Arginyl
DILT_0001480801-mRNA-1	Seryl
DILT_0001528001-mRNA-1	Asparaginyl
DILT_0001540201-mRNA-1	Lysyl
DILT_0001573901-mRNA-1	Tyrosyl
DILT_0001616401-mRNA-1	Glutamyl
DILT_0001690201-mRNA-1	Tryptophanyl
DILT_0001828001-mRNA-1	Isoleucyl
DILT_0001855801-mRNA-1	threonyl
DILT_0001864201-mRNA-1	Glutaminyl
DILT_0001892101-mRNA-1	Leucyl
DILT_0001931501-mRNA-1	Tryptophanyl

Dracunculus medinensis	aaRSs
DME_0000011101-mRNA-1	Valyl
DME_0000048201-mRNA-1	Glycyl
DME_0000057101-mRNA-1	Arginyl
DME_0000087301-mRNA-1	Asparaginyl
DME_0000144701-mRNA-1	Glutaminyl
DME_0000183601-mRNA-1	PhenylAlanyl
DME_0000219401-mRNA-1	Prolyl
DME_0000289001-mRNA-1	Prolyl
DME_0000296501-mRNA-1	Isoleucyl
DME_0000302101-mRNA-1	Aspartyl
DME_0000322001-mRNA-1	Seryl
DME_0000372201-mRNA-1	Cysteinyl
DME_0000404601-mRNA-1	Asparaginyl
DME_0000489201-mRNA-1	Methionyl
DME_0000518801-mRNA-1	Tyrosyl
DME_0000521701-mRNA-1	Seryl
DME_0000547901-mRNA-1	Alanyl
DME_0000550201-mRNA-1	Prolyl
DME_0000578101-mRNA-1	Alanyl
DME_0000629901-mRNA-1	Arginyl
DME_0000654601-mRNA-1	Phenylalanyl
DME_0000699401-mRNA-1	Leucyl
DME_0000724601-mRNA-1	Tyrosyl
DME_0000729901-mRNA-1	Histidyl
DME_0000764501-mRNA-1	Methionyl
DME_0000849701-mRNA-1	Tryptophanyl
DME_0000866101-mRNA-1	Lysyl
DME_0000866701-mRNA-1	Glutamyl
DME_0000877501-mRNA-1	Isoleucyl
DME_0001024401-mRNA-1	Leucyl
DME_0001026001-mRNA-1	threonyl
DME_0001041101-mRNA-1	Valyl
DME_0001041301-mRNA-1	Valyl

DME_0001068101-mRNA-1	Aspartyl
DME_0001070301-mRNA-1	Tryptophanyl
Echinococcus granulosus	aaRSs
EgrG_000972000	Methionyl
EgrG_000520000	Prolyl
EgrG_000976400	Glycyl
EgrG_000348100	Arginyl
EgrG_000248300	Glutaminyl
EgrG_000435000	Valyl
EgrG_000104900	Leucyl
EgrG_000889700	Asparaginyl
EgrG_001147800	Tyrosyl
EgrG_000239900	Seryl
EgrG_000186800	Methionyl
EgrG_002008700	Isoleucyl
EgrG_000461800	Prolyl
EgrG_000861400	Leucyl
EgrG_001147900	PhenylAlanyl
EgrG_001197300	Seryl
EgrG_000095100	Arginyl
EgrG_002026800	Asparaginyl
EgrG_000629500	Lysyl
EgrG_000437500	Alanyl
EgrG_000932500	Phenylalanyl
EgrG_001008100	Histidyl
EgrG_000394100	Tryptophanyl
EgrG_000759100	Tryptophanyl
EgrG_000440600	Alanyl
EgrG_000793800	Cysteinyl
EgrG_000888800	Glutamyl
EgrG_000375800	Threonyl
EgrG_000348600	Asparaginyl
EgrG_000527000	Valyl
EgrG_000754600	Isoleucyl
EgrG_000777100	Aspartyl
EgrG_000901500	Tyrosyl
Echinococcus multilocularis	aaRSs
EmuJ_000095100.1	Arginyl
EmuJ_000104900.1	Leucyl
EmuJ_000186800.1	Methionyl
EmuJ_000239900.1	Seryl
EmuJ_000248300.1	Glutaminyl
EmuJ_000296500.1	Isoleucyl
EmuJ_000348100.1	Arginyl
EmuJ_000348300.1	Asparaginyl
EmuJ_000348600.1	Asparaginyl
EmuJ_000348700.1	Asparaginyl
EmuJ_000375800.1	Threonyl
EmuJ_000394100.1	Tryptophanyl
EmuJ_000435000.1	Valyl

EmuJ_000437500.1	Alanyl
EmuJ_000440600.1	Alanyl
EmuJ_000461800.1	Prolyl
EmuJ_000520000.1	Prolyl
EmuJ_000527000.1	Valyl
EmuJ_000629500.1	Lysyl
EmuJ_000754600.1	Isoleucyl
EmuJ_000759100.1	Tryptophanyl
EmuJ_000777100.1	Aspartyl
EmuJ_000793800.1	Cysteinyl
EmuJ_000861400.1	Leucyl
EmuJ_000888800.1	Glutamyl
EmuJ_000889700.1	Asparaginyl
EmuJ_000901500.1	Tyrosyl
EmuJ_000932500.1	Phenylalanyl
EmuJ_000972000.1	Methionyl
EmuJ_000976400.1	Glycyl
EmuJ_001008100.1	Histidyl
EmuJ_001147800.1	Tyrosyl
EmuJ_001147900.1	Phenylalanyl
EmuJ_001197300.1	Seryl
EmuJ_002101900.1	Aspartyl

Fasciola hepatica	aaRSs
D915_15485	Glutamyl
D915_07668	PhenylAlanyl
D915_12728	Lysyl
D915_04166	Methionyl
D915_12771	Tryptophanyl
D915_15934	Alanyl
D915_03300	Cysteinyl
D915_10078	Glutaminyl
D915_04073	Glutaminyl
D915_00715	Leucyl
D915_14857	PhenylAlanyl
D915_15471	Aspartyl
D915_02816	Histidyl
D915_13996	Isoleucyl
D915_14758	Alanyl
D915_12769	Tryptophanyl
D915_15382	Isoleucyl
D915_03274	Tyrosyl
D915_07610	Tyrosyl
D915_12414	Leucyl
D915_11237	Prolyl
D915_02080	Valyl
D915_09139	Asparaginyl
D915_15002	Tryptophanyl
D915_01628	Alanyl
D915_06994	Aspartyl
D915_14311	Leucyl
D915_03166	Arginyl

D915_15509	Seryl
D915_15939	Leucyl
D915_10079	Glutaminyl
D915_15358	Lysyl
D915_00713	Leucyl
D915_06032	Aspartyl
D915_13998	Isoleucyl
D915_15897	Alanyl
D915_10077	Glutaminyl
D915_15896	Alanyl
D915_13975	Glutamyl
D915_12770	Tryptophanyl
D915_14856	PhenylAlanyl
D915_03348	Tryptophanyl
D915_14106	Methionyl
D915_14414	Isoleucyl
D915_13997	Isoleucyl
D915_04076	Prolyl
D915_06033	Aspartyl
D915_14415	Isoleucyl
D915_09784	Valyl
D915_03301	Cysteinyl
D915_02817	Histidyl
D915_14757	Alanyl
D915_02081	Valyl
D915_13096	Glycyl
D915_15435	Valyl
D915_15547	Alanyl
D915_06401	Threonyl
D915_01627	Alanyl
D915_12727	Lysyl
D915_14824	threonyl
D915_15606	Glutamyl
D915_15245	Tyrosyl
D915_04614	Seryl
D915_15568	Arginyl
D915_04074	Glutamyl
D915_06993	Aspartyl
D915_15261	Methionyl
D915_15252	Cysteinyl
D915_15589	Isoleucyl

Hymenolepis diminuta	aaRSs
HDID_0000011001-mRNA-1	Valyl
HDID_0000014201-mRNA-1	Alanyl
HDID_0000036901-mRNA-1	Tryptophanyl
HDID_0000058101-mRNA-1	Glutaminyl
HDID_0000075901-mRNA-1	Glutamyl
HDID_0000128801-mRNA-1	Prolyl
HDID_0000131401-mRNA-1	Aspartyl
HDID_0000159301-mRNA-1	Glycyl
HDID_0000196101-mRNA-1	Tryptophanyl

HDID_0000228001-mRNA-1	Arginyl
HDID_0000271901-mRNA-1	Prolyl
HDID_0000312701-mRNA-1	Alanyl
HDID_0000353601-mRNA-1	Leucyl
HDID_0000354901-mRNA-1	Isoleucyl
HDID_0000365001-mRNA-1	Histidyl
HDID_0000391101-mRNA-1	Glutaminyl
HDID_0000416601-mRNA-1	Seryl
HDID_0000453901-mRNA-1	Alanyl
HDID_0000478501-mRNA-1	Aspartyl
HDID_0000521801-mRNA-1	Asparaginyl
HDID_0000528501-mRNA-1	threonyl
HDID_0000609801-mRNA-1	Isoleucyl
HDID_0000675801-mRNA-1	Asparaginyl
HDID_0000676001-mRNA-1	Arginyl
HDID_0000704501-mRNA-1	Leucyl
HDID_0000722401-mRNA-1	Seryl
HDID_0000748501-mRNA-1	Tyrosyl
HDID_0000758901-mRNA-1	Methionyl
HDID_0000791701-mRNA-1	Alanyl
HDID_0000848501-mRNA-1	Methionyl
HDID_0000875201-mRNA-1	Tyrosyl
HDID_0000929601-mRNA-1	Phenylalanyl
HDID_0000951301-mRNA-1	Cysteinyl
HDID_0000985301-mRNA-1	Lysyl
HDID_0001039201-mRNA-1	Valyl
HDID_0001060701-mRNA-1	Isoleucyl
HDID_0001090001-mRNA-1	Phenylalanyl
HDID_0001090801-mRNA-1	Isoleucyl
Hymenolepis mirostoma	
HmN_000000100.1	Tyrosyl
HmN_000043900.1	Aspartyl
HmN_000055600.1	Valyl
HmN_000057000.1	Prolyl
HmN_000119700.1	Methionyl
HmN_000147200.1	Methionyl
HmN_000152500.1	Arginyl
HmN_000161600.1	Histidyl
HmN_000166200.1	Seryl
HmN_000177600.1	Isoleucyl
HmN_000195600.1	Seryl
HmN_000326900.1	Arginyl
HmN_000327000.1	Asparaginyl
HmN_000327100.1	Asparaginyl
HmN_000368500.1	Threonyl
HmN_000369000.1	Glycyl
HmN_000434500.1	Cysteinyl
HmN_000450800.1	Phenylalanyl
HmN_000458500.1	Aspartyl
HmN_000470300.1	Glutaminyl
HmN_000495200.1	Alanyl

HmN_000496200.1	Valyl
HmN_000637600.1	Tryptophanyl
HmN_000648700.1	Tryptophanyl
HmN_000660100.1	Prolyl
HmN_000729500.1	Alanyl
HmN_000753800.1	Leucyl
HmN_000830300.1	Isoleucyl
HmN_000852000.1	Tyrosyl
HmN_002144100.1	Glutamyl
HmN_002145200.1	Asparaginyl
HmN_002193200.1	Lysyl
HmN_002221500.1	PhenylAlanyl
Hymenolepis nana	
HNAJ_0000079901-mRNA-1	Valyl
HNAJ_0000141301-mRNA-1	Seryl
HNAJ_0000187501-mRNA-1	Glutamyl
HNAJ_0000212901-mRNA-1	Leucyl
HNAJ_0000248201-mRNA-1	Threonyl
HNAJ_0000269001-mRNA-1	Alanyl
HNAJ_0000272201-mRNA-1	Cysteinyl
HNAJ_0000308801-mRNA-1	Leucyl
HNAJ_0000308901-mRNA-1	Arginyl
HNAJ_0000309001-mRNA-1	Asparaginyl
HNAJ_0000362001-mRNA-1	Asparaginyl
HNAJ_0000363601-mRNA-1	Tryptophanyl
HNAJ_0000370501-mRNA-1	Aspartyl
HNAJ_0000374601-mRNA-1	Glycyl
HNAJ_0000392801-mRNA-1	Valyl
HNAJ_0000395401-mRNA-1	Alanyl
HNAJ_0000466301-mRNA-1	Glutaminyl
HNAJ_0000513201-mRNA-1	PhenylAlanyl
HNAJ_0000548601-mRNA-1	Lysyl
HNAJ_0000612001-mRNA-1	Methionyl
HNAJ_0000717901-mRNA-1	Alanyl
HNAJ_0000748001-mRNA-1	Asparaginyl
HNAJ_0000782901-mRNA-1	PhenylAlanyl
HNAJ_0000783201-mRNA-1	Tyrosyl
HNAJ_0000882001-mRNA-1	Tyrosyl
HNAJ_0000884601-mRNA-1	Arginyl
HNAJ_0000906401-mRNA-1	Prolyl
HNAJ_0000919601-mRNA-1	Isoleucyl
HNAJ_0000996701-mRNA-1	Methionyl
HNAJ_0001038901-mRNA-1	Methionyl
HNAJ_0001051501-mRNA-1	Leucyl
HNAJ_0001056101-mRNA-1	Prolyl
HNAJ_0001103801-mRNA-1	Histidyl
HNAJ_0001113701-mRNA-1	Valyl
HNAJ_0001188701-mRNA-1	Aspartyl
HNAJ_0001205801-mRNA-1	Isoleucyl
HNAJ_0001272801-mRNA-1	Glutaminyl
HNAJ_0001358301-mRNA-1	Asparaginyl

Loa loa	aaRSs
EN70_1	Methionyl
EN70_1014	Glutamyl
EN70_10305	Asparaginyl
EN70_10426	Glutaminyl
EN70_10543	Threonyl
EN70_10784	Alanyl
EN70_11050	Methionyl
EN70_1212	Arginyl
EN70_12146	Cysteinyl
EN70_1252	Alanyl
EN70_1258	Leucyl
EN70_283	Seryl
EN70_3457	Tryptophanyl
EN70_3516	Aspartyl
EN70_3530	Seryl
EN70_4466	Methionyl
EN70_4745	Prolyl
EN70_4746	Glutaminyl
EN70_4912	Glutamyl
EN70_5052	Lysyl
EN70_5058	PhenylAlanyl
EN70_5214	Asparaginyl
EN70_5331	Tyrosyl
EN70_5345	Aspartyl
EN70_5346	Aspartyl
EN70_5411	Tryptophanyl
EN70_5799	Valyl
EN70_5800	PhenylAlanyl
EN70_6631	Glycyl
EN70_6707	Isoleucyl
EN70_7547	Prolyl
EN70_7700	Isoleucyl
EN70_8096	Prolyl
EN70_8395	Tyrosyl
EN70_8755	Valyl
EN70_8849	Histidyl
EN70_9796	Leucyl
EN70_9908	Arginyl

Necator americanus	aaRSs
NECAME_00719	Alanyl
NECAME_00844	Leucyl
NECAME_00891	Glycyl
NECAME_01037	Prolyl
NECAME_01128	threonyl
NECAME_01233	PhenylAlanyl
NECAME_02085	Glutaminyl
NECAME_02629	Seryl
NECAME_03481	Arginyl
NECAME_03562	Histidyl

NECAME_03860	Glutamyl
NECAME_04406	PhenylAlanyl
NECAME_05073	Seryl
NECAME_05692	Valyl
NECAME_05693	Valyl
NECAME_06000	Asparaginyl
NECAME_06002	threonyl
NECAME_06303	Glutaminyl
NECAME_06916	Tryptophanyl
NECAME_07680	Isoleucyl
NECAME_08865	Arginyl
NECAME_09221	Cysteinyl
NECAME_10241	Tryptophanyl
NECAME_10242	Tryptophanyl
NECAME_10420	Alanyl
NECAME_10498	Valyl
NECAME_11083	Aspartyl
NECAME_11885	Lysyl
NECAME_11931	Isoleucyl
NECAME_12225	Methionyl
NECAME_14660	Prolyl
NECAME_15187	Aspartyl
NECAME_15188	Aspartyl
NECAME_16052	Tyrosyl
NECAME_16192	Tyrosyl
NECAME_16774	Methionyl
NECAME_16854	Asparaginyl
NECAME_17299	Aspartyl
NECAME_18683	Valyl
NECAME_19565	Aspartyl

Onchocerca volvulus	aaRSs
OVOC10390	Alanyl
OVOC10793	Arginyl
OVOC11137	Histidyl
OVOC11195	Glutamyl
OVOC11365a	Prolyl
OVOC11631	Methionyl
OVOC1179	Prolyl
OVOC11842	Prolyl
OVOC13489	Valyl
OVOC1573	Leucyl
OVOC1585	Alanyl
OVOC193	Cysteinyl
OVOC2324	Glutamyl
OVOC2471	threonyl
OVOC2562	PhenylAlanyl
OVOC2569a	Lysyl
OVOC2679	Cysteinyl
OVOC3179	Methionyl
OVOC459	Isoleucyl
OVOC4642	Tyrosyl

OVOC5077	Seryl
OVOC6300	Isoleucyl
OVOC6356	Tryptophanyl
OVOC6432	Aspartyl
OVOC6455	Tyrosyl
OVOC6597	Asparaginyl
OVOC6599	Asparaginyl
OVOC7102	PhenylAlanyl
OVOC7103	Valyl
OVOC8035	Leucyl
OVOC820	Isoleucyl
OVOC8625	Tryptophanyl
OVOC8700	Aspartyl
OVOC8714	Seryl
OVOC8719	Seryl
OVOC8962	Glycyl
OVOC9040	Asparaginyl
OVOC9151	Arginyl
OVOC9483	Glutaminyl

Ophisthorchis viverrini	aaRSs
T265_14558	Prolyl
T265_08843	Isoleucyl
T265_08946	Lysyl
T265_09084	Tryptophanyl
T265_14849	Asparaginyl
T265_09842	Cysteinyl
T265_10380	Alanyl
T265_01087	threonyl
T265_11299	Tyrosyl
T265_11386	Leucyl
T265_11659	Arginyl
T265_15554	Tryptophanyl
T265_15582	Valyl
T265_01274	Leucyl
T265_16294	Isoleucyl
T265_02630	Prolyl
T265_02835	Seryl
T265_03456	Alanyl
T265_03819	Arginyl
T265_03894	Seryl
T265_04925	Histidyl
T265_05270	Tyrosyl
T265_05275	Asparaginyl
T265_13744	Aspartyl
T265_13748	Valyl
T265_13749	Valyl
T265_05757	Methionyl
T265_13851	Methionyl
T265_13902	Aspartyl
T265_00610	Glycyl
T265_06528	Glutaminyl

T265_07520	Glutamyl
T265_14369	PhenylAlanyl
T265_07791	Methionyl
T265_07943	Isoleucyl
Schistosoma haematobium	aaRSs
A_01599	Prolyl
A_04057	threonyl
A_07093	Glutamyl
A_01184	Valyl
A_02141	Histidyl
A_07424	Valyl
B_00613	Lysyl
A_02332	PhenylAlanyl
A_08080	Isoleucyl
A_03316	Isoleucyl
C_00986	Glutamyl
A_06086	Methionyl
A_07337	Tryptophanyl
A_00749	Seryl
B_00444	Cysteinyl
B_00258	Leucyl
A_01043	Leucyl
A_00384	Alanyl
A_03318	Leucyl
A_08373	Arginyl
A_04059	Arginyl
A_00552	Alanyl
A_06406	Methionyl
A_01273	Tryptophanyl
A_02925	Aspartyl
A_04779	Asparaginyl
B_00823	Asparaginyl
A_02305	PhenylAlanyl
A_00703	Tryptophanyl
A_06665	Glutaminyl
B_00054	Tryptophanyl
A_04168	Aspartyl
A_07113	Glycyl
B_00563	Lysyl
A_08183	Tyrosyl
B_00498	Tyrosyl
A_07985	Prolyl
Schistosoma japonicum	aaRSs
Sjp_0009980	Isoleucyl
Sjp_0056410	Leucyl
Sjp_0021140	Glutaminyl
Sjp_0072210	Aspartyl
Sjp_0072220	Aspartyl
Sjp_0037370	Methionyl
Sjp_0047670	Methionyl

Sjp_0122730	Cysteinyl
Sjp_0054640	Prolyl
Sjp_0010800	Lysyl
Sjp_0105270	Methionyl
Sjp_0068870	Valyl
Sjp_0050100	Leucyl
Sjp_0075820	Seryl
Sjp_0124150	Alanyl
Sjp_0088910	Alanyl
Sjp_0085650	Arginyl
Sjp_0077090	Leucyl
Sjp_0072860	Tyrosyl
Sjp_0038640	Arginyl
Sjp_0037460	Asparaginyl
Sjp_0052070	Seryl
Sjp_0133030	Tyrosyl
Sjp_0092060	Tryptophanyl
Sjp_0051950	Aspartyl
Sjp_0075600	Alanyl
Sjp_0053670	Asparaginyl
Sjp_0007710	Histidyl
Sjp_0043850	Lysyl
Sjp_0106740	Isoleucyl
Sjp_0080790	PhenylAlanyl
Sjp_0105480	Isoleucyl
Sjp_0002700	Valyl
Sjp_0066020	Glutamyl
Sjp_0061760	Alanyl
Sjp_0013140	Isoleucyl
Sjp_0030360	Tryptophanyl
Sjp_0117790	Prolyl

Schistosoma mansoni	aaRSs
Smp_005330.1	Methionyl
Smp_009520.1	Glutamyl
Smp_023360.1	Histidyl
Smp_025260.1	Prolyl
Smp_038240.1	Alanyl
Smp_040770.1	Methionyl
Smp_040800.1	Glycyl
Smp_041450.1	Asparaginyl
Smp_041600.1	Isoleucyl
Smp_053510.1	Aspartyl
Smp_053610.1	Seryl
Smp_057230.1	Seryl
Smp_082860.1	Tryptophanyl
Smp_088800.1	Aspartyl
Smp_096270.1	Valyl
Smp_097590.1	Valyl
Smp_101230.1	PhenylAlanyl
Smp_101240.1	PhenylAlanyl
Smp_104470.1	Lysyl

Smp_122780.1	Leucyl
Smp_129210.1	Tyrosyl
Smp_129650.1	Isoleucyl
Smp_133280.1	Arginyl
Smp_135400.1	Alanyl
Smp_138930.2	Prolyl
Smp_148050.1	Glutaminyl
Smp_149430.1	PhenylAlanyl
Smp_150680.1	Tryptophanyl
Smp_153430.1	Arginyl
Smp_169030.1	Asparaginyl
Smp_170800.2	Cysteinyl
Smp_176290.1	Tyrosyl
Smp_179140.1	Aspartyl
Smp_190720.1	Aspartyl
Smp_194140.1	Aspartyl
Smp_194150.1	Aspartyl
Smp_194160.1	Leucyl
Smp_199340.1	Aspartyl
Strongyloides stercoralis	aaRSs
SSTP_0000084000.1	Lysyl
SSTP_0000088600.1	threonyl
SSTP_0000150200.1	Asparaginyl
SSTP_0000197400.1	Glycyl
SSTP_0000213200.1	PhenylAlanyl
SSTP_0000230400.1	Glutamyl
SSTP_0000296300.1	Valyl
SSTP_0000322900.1	Arginyl
SSTP_0000337100.1	Histidyl
SSTP_0000374100.1	Glutaminyl
SSTP_0000394900.1	Isoleucyl
SSTP_0000461100.1	Asparaginyl
SSTP_0000603200.1	Tryptophanyl
SSTP_0000627200.1	Glutaminyl
SSTP_0000652400.1	Alanyl
SSTP_0000654000.1	Prolyl
SSTP_0000657200.1	Arginyl
SSTP_0000734350.1	PhenylAlanyl
SSTP_0000753600.1	Leucyl
SSTP_0000773200.1	Prolyl
SSTP_0000787100.1	Seryl
SSTP_0000803500.1	Leucyl
SSTP_0000900000.1	Aspartyl
SSTP_0000924200.1	Valyl
SSTP_0000927100.1	Aspartyl
SSTP_0000944800.1	Alanyl
SSTP_0000978500.1	Tyrosyl
SSTP_0001174200.1	Cysteinyl
SSTP_0001180000.1	Tyrosyl
SSTP_0001200800.1	Methionyl
SSTP_0001219100.1	Seryl

SSTP_0001221300.1	Methionyl
SSTP_0001261600.1	Tryptophanyl
Taenia asiatica	aaRSs
TASK_0000070501-mRNA-1	Histidyl
TASK_0000081301-mRNA-1	Arginyl
TASK_0000158301-mRNA-1	Arginyl
TASK_0000175501-mRNA-1	Methionyl
TASK_0000278001-mRNA-1	Seryl
TASK_0000315801-mRNA-1	Glutaminyl
TASK_0000321701-mRNA-1	Valyl
TASK_0000364801-mRNA-1	Alanyl
TASK_0000415101-mRNA-1	Tryptophanyl
TASK_0000426301-mRNA-1	Glutaminyl
TASK_0000455501-mRNA-1	Threonyl
TASK_0000469601-mRNA-1	Leucyl
TASK_0000472401-mRNA-1	Lysyl
TASK_0000488901-mRNA-1	Valyl
TASK_0000511501-mRNA-1	Prolyl
TASK_0000532601-mRNA-1	PhenylAlanyl
TASK_0000532801-mRNA-1	Tyrosyl
TASK_0000535501-mRNA-1	Cysteinyl
TASK_0000548301-mRNA-1	Aspartyl
TASK_0000568301-mRNA-1	Asparaginyl
TASK_0000685401-mRNA-1	Aspartyl
TASK_0000695801-mRNA-1	Seryl
TASK_0000718401-mRNA-1	Isoleucyl
TASK_0000729501-mRNA-1	Alanyl
TASK_0000758701-mRNA-1	Glutamyl
TASK_0000788501-mRNA-1	Glycyl
TASK_0000831801-mRNA-1	Isoleucyl
TASK_0000864801-mRNA-1	Tryptophanyl
TASK_0000870001-mRNA-1	Leucyl
TASK_0000898701-mRNA-1	Asparaginyl
TASK_0000902501-mRNA-1	Methionyl
TASK_0000956901-mRNA-1	PhenylAlanyl
Taenia saginata	aaRSs
TSAs00001g00232m00001	Glutaminyl
TSAs00001g00308m00001	Seryl
TSAs00003g01004m00001	Glutamyl
TSAs00003g01010m00001	Asparaginyl
TSAs00003g01100m00001	Tyrosyl
TSAs00006g01626m00001	Aspartyl
TSAs00006g01758m00001	Cysteinyl
TSAs00010g02399m00001	Valyl
TSAs00010g02417m00001	Alanyl
TSAs00010g02441m00001	Alanyl
TSAs00011g02653m00001	Lysyl
TSAs00013g02841m00001	Methionyl
TSAs00014g02967m00001	Leucyl
TSAs00015g03193m00001	Histidyl

TSAs00019g03660m00001	Aspartyl
TSAs00020g03757m00001	Isoleucyl
TSAs00024g04081m00001	PhenylAlanyl
TSAs00032g04889m00001	Threonyl
TSAs00039g05536m00001	Arginyl
TSAs00045g06038m00001	Prolyl
TSAs00052g06539m00001	Leucyl
TSAs00054g06661m00001	Methionyl
TSAs00060g07040m00001	Tryptophanyl
TSAs00065g07297m00001	Glycyl
TSAs00067g07399m00001	Seryl
TSAs00067g07402m00001	Seryl
TSAs00070g07595m00001	Tyrosyl
TSAs00070g07597m00001	PhenylAlanyl
TSAs00071g07636m00001	Isoleucyl
TSAs00074g07734m00001	Valyl
TSAs00090g08306m00001	Prolyl
TSAs00092g08357m00001	Tryptophanyl
TSAs00116g08895m00001	Arginyl
TSAs00116g08897m00001	Asparaginyl

Taenia solium	aaRSs
TsM_000644900	Methionyl
TsM_000555300	Asparaginyl
TsM_000244400	Alanyl
TsM_000721100	Aspartyl
TsM_000521500	Seryl
TsM_001155800	Cysteinyl
TsM_000029200	Tyrosyl
TsM_000648300	Phenylalanyl
TsM_001063400	Histidyl
TsM_000839800	Tryptophanyl
TsM_000698100	Alanyl
TsM_000536400	Valyl
TsM_000773100	Isoleucyl
TsM_000809000	Aspartyl
TsM_000192200	Threonyl
TsM_000996100	Isoleucyl
TsM_000542800	Leucyl
TsM_001085300	Prolyl
TsM_000773500	Methionyl
TsM_000330700	Glutamyl
TsM_000620600	Glutaminyl
TsM_000887200	Tryptophanyl
TsM_000846600	Arginyl
TsM_000729400	Glycyl
TsM_001228300	Tyrosyl
TsM_001034600	Valyl
TsM_000900500	Lysyl
TsM_001077300	Leucyl
TsM_000665500	Asparaginyl
TsM_000828100	PhenylAlanyl

TsM_001071600	Methionyl
TsM_001029600	Arginyl
TsM_000894900	Seryl
Trichinella spiralis	aaRSs
EFV58402	Arginyl
EFV58317	Methionyl
EFV58263	Histidyl
EFV58184	Leucyl
EFV57903	Prolyl
EFV57522	Glycyl
EFV61250	Seryl
EFV60730	Phenylalanyl
EFV55995	Glutamyl
EFV55653	Isoleucyl
EFV51304	Tryptophanyl
EFV50681	Seryl
EFV56401	Asparaginyl
EFV59837	Valyl
EFV59666	Methionyl
EFV59613	Lysyl
EFV62542	Asparaginyl
EFV54413	Isoleucyl
EFV54261	Aspartyl
EFV55105	Glutaminyl
EFV53828	Leucyl
EFV61767	Prolyl
EFV61632	Tyrosyl
EFV54757	Tyrosyl
EFV54739	Cysteinyl
EFV54681	Valyl
EFV51596	Alanyl
EFV50282	PhenylAlanyl
EFV52543	Alanyl
EFV53336	Arginyl
EFV59868	threonyl
EFV49888	threonyl
EFV49577	Valyl
EFV49576	Valyl
EFV49113	Glutaminyl
EFV48446	Leucyl
EFV48184	Prolyl
Trichuris trichiura	aaRSs
TTRE_0000013401-mRNA-1	Asparaginyl
TTRE_0000029701-mRNA-1	Valyl
TTRE_0000046401-mRNA-1	Threonyl
TTRE_0000049201-mRNA-1	Glutamyl
TTRE_0000058801-mRNA-1	Phenylalanyl
TTRE_0000143501-mRNA-1	Glutaminyl
TTRE_0000200701-mRNA-1	Leucyl
TTRE_0000228101-mRNA-1	Isoleucyl

TTRE_0000229401-mRNA-1	Seryl
TTRE_0000233201-mRNA-1	Lysyl
TTRE_0000258501-mRNA-1	Valyl
TTRE_0000273301-mRNA-1	Tyrosyl
TTRE_0000292901-mRNA-1	Methionyl
TTRE_0000305301-mRNA-1	Phenylalanyl
TTRE_0000394101-mRNA-1	Aspartyl
TTRE_0000413801-mRNA-1	Aspartyl
TTRE_0000416201-mRNA-1	Tryptophanyl
TTRE_0000426701-mRNA-1	Arginyl
TTRE_0000457401-mRNA-1	Tryptophanyl
TTRE_0000465101-mRNA-1	Seryl
TTRE_0000465901-mRNA-1	Glycyl
TTRE_0000474501-mRNA-1	Cysteinyl
TTRE_0000478601-mRNA-1	Asparaginyl
TTRE_0000482001-mRNA-1	Prolyl
TTRE_0000525201-mRNA-1	Lysyl
TTRE_0000526901-mRNA-1	Lysyl
TTRE_0000569901-mRNA-1	Methionyl
TTRE_0000593301-mRNA-1	Tyrosyl
TTRE_0000602301-mRNA-1	Alanyl
TTRE_0000625901-mRNA-1	Isoleucyl
TTRE_0000642101-mRNA-1	Alanyl
TTRE_0000691801-mRNA-1	Arginyl
TTRE_0000730701-mRNA-1	Leucyl
TTRE_0000776101-mRNA-1	Valyl
TTRE_0000783401-mRNA-1	Histidyl
TTRE_0000806801-mRNA-1	Alanyl
TTRE_0000811301-mRNA-1	Histidyl
TTRE_0000814101-mRNA-1	Prolyl
TTRE_0000820201-mRNA-1	Prolyl
TTRE_0000824901-mRNA-1	Cysteinyl
TTRE_0000825101-mRNA-1	Glutamyl
TTRE_0000826101-mRNA-1	Arginyl
TTRE_0000828401-mRNA-1	Alanyl
TTRE_0000847801-mRNA-1	Leucyl
TTRE_0000861901-mRNA-1	Glutaminyl
TTRE_0000864901-mRNA-1	Valyl
TTRE_0000885801-mRNA-1	Glutaminyl
TTRE_0000892301-mRNA-1	Tryptophanyl
TTRE_0000915101-mRNA-1	Leucyl
TTRE_0000926601-mRNA-1	threonyl
TTRE_0000926701-mRNA-1	PhenylAlanyl
TTRE_0000966201-mRNA-1	Glutaminyl

Wuchereria bancrofti	aaRSs
maker-PairedContig_1194-snap-gene-0.2-mRNA-1	Aspartyl
maker-PairedContig_1198-snap-gene-4.15-mRNA-1	Tyrosyl
maker-PairedContig_1326-snap-gene-0.4-mRNA-1	Glutamyl
maker-PairedContig_1338-snap-gene-1.11-mRNA-1	Tryptophanyl
maker-PairedContig_1459-snap-gene-0.11-mRNA-1	Asparaginyl
maker-PairedContig_151-snap-gene-0.2-mRNA-1	Prolyl

maker-PairedContig_1555-snap-gene-0.5-mRNA-1	Phenylalanyl
maker-PairedContig_1555-snap-gene-0.6-mRNA-1	Valyl
maker-PairedContig_16-snap-gene-0.3-mRNA-1	Valyl
maker-PairedContig_1625-snap-gene-2.36-mRNA-1	Arginyl
maker-PairedContig_1679-snap-gene-0.25-mRNA-1	Lysyl
maker-PairedContig_1679-snap-gene-0.28-mRNA-1	PhenylAlanyl
maker-PairedContig_1765-snap-gene-0.5-mRNA-1	Glutaminyl
maker-PairedContig_1808-snap-gene-0.20-mRNA-1	Methionyl
maker-PairedContig_201-snap-gene-0.9-mRNA-1	Prolyl
maker-PairedContig_2047-snap-gene-1.25-mRNA-1	Glycyl
maker-PairedContig_206-snap-gene-6.13-mRNA-1	Tryptophanyl
maker-PairedContig_2062-snap-gene-1.12-mRNA-1	Asparaginyl
maker-PairedContig_2091-snap-gene-7.12-mRNA-1	Alanyl
maker-PairedContig_2091-snap-gene-8.15-mRNA-1	Leucyl
maker-PairedContig_3141-snap-gene-0.5-mRNA-1	Leucyl
maker-PairedContig_3580-snap-gene-0.4-mRNA-1	Tyrosyl
maker-PairedContig_4131-snap-gene-0.5-mRNA-1	Aspartyl
maker-PairedContig_432-snap-gene-1.12-mRNA-1	Isoleucyl
maker-PairedContig_4489-snap-gene-0.16-mRNA-1	Alanyl
maker-PairedContig_467-snap-gene-0.15-mRNA-1	Methionyl
maker-PairedContig_4689-snap-gene-1.14-mRNA-1	Seryl
maker-PairedContig_5379-snap-gene-2.26-mRNA-1	Histidyl
maker-PairedContig_5864-snap-gene-0.9-mRNA-1	Prolyl
maker-PairedContig_5969-snap-gene-0.12-mRNA-1	Methionyl
maker-PairedContig_650-snap-gene-1.19-mRNA-1	Isoleucyl
maker-PairedContig_979-snap-gene-0.3-mRNA-1	Cysteinyl
snap_masked-PairedContig_1070-processed-gene-0.0-mRNA-1	Valyl
snap_masked-PairedContig_3489-processed-gene-0.1-mRNA-1	Arginyl
snap_masked-PairedContig_4361-processed-gene-0.0-mRNA-1	Glutamyl
snap_masked-PairedContig_5411-processed-gene-8.11-mRNA-1	Seryl
snap_masked-PairedContig_6294-processed-gene-1.0-mRNA-1	Threonyl
