# Supplementary material for: Genomic analyses of aminoacyl tRNA synthetases from human-infecting helminths
Source: BMC Genomics. 2019 May 2;20:333. doi: 10.1186/s12864-019-5679-0 (PMC6498573; doi:10.1186/s12864-019-5679-0)
Supplement: Supplementary file 2 — Table S2. Domain description of all the aaRSs like proteins showing the presence of unusual domains. Unusual domains are mentioned in red. (PDF 99 kb) [file 12864_2019_5679_MOESM2_ESM.pdf]

Helminths Gene IDs	pfam_domains
ANCDUO_12845	tRNA-synt_1;tRNA-synt_1g
ANCDUO_05963	tRNA-synt_1d;DALR_1
ANCDUO_18591	tRNA-synt_1d;Arg_tRNA_synt_N
ANCDUO_08798	tRNA-synt_1;Anticodon_1;tRNA-synt_1g
ANCDUO_25895	tRNA-synt_2c
ANCDUO_09533	tRNA-synt_2c;MRP-S23;tRNA_SAD;DHHA1
ANCDUO_17345	tRNA-synt_2c
ANCDUO_05820	tRNA-synt_2
ANCDUO_13630	tRNA-synt_2
ANCDUO_26463	tRNA-synt_2c
ANCDUO_20791	tRNA-synt_2b
ANCDUO_19811	tRNA-synt_2d
ANCDUO_02067	tRNA-synt_1e;tRNA-synt_1g
ANCDUO_18955	tRNA-synt_2;tRNA_anti-codon
ANCDUO_24055	tRNA-synt_1b
ANCDUO_24911	tRNA-synt_2b
ANCDUO_18234	tRNA-synt_2b
ANCDUO_19768	tRNA-synt_2d
ANCDUO_19068	tRNA-synt_His;WHEP-TRS
ANCDUO_27496	tRNA-synt_1b
ANCDUO_15714	tRNA-synt_2b
ANCDUO_00820	tRNA-synt_1g;tRNA_bind;tRNA-synt_1
ANCDUO_25110	tRNA-synt_1
ANCDUO_11265	tRNA-synt_1b
ANCDUO_18661	tRNA-synt_1c;tRNA-synt_1e
ANCDUO_09186	tRNA-synt_His;WHEP-TRS
ANCDUO_00405	tRNA-synt_2;tRNA_anti-codon
ANCDUO_16790	tRNA-synt_2;tRNA_anti-codon;tRNA-synt_2d
ANCDUO_03641	tRNA-synt_1c
ANCDUO_18953	tRNA-synt_2
ANCDUO_09370	tRNA-synt_1b
ANCDUO_24027	tRNA-synt_2c
ANCDUO_24251	tRNA-synt_1
ANCDUO_03331	tRNA-synt_1d;DALR_1
ANCDUO_07419	tRNA-synt_2b
ANCDUO_11081	tRNA-synt_2c;MRP-S23
ANCDUO_15560	tRNA-synt_2b
ANCDUO_10383	tRNA-synt_1;Anticodon_1;tRNA-synt_1g
ANCDUO_14858	tRNA-synt_2b;TGS;tRNA_SAD;HGTP_anticodon
ANCDUO_18662	tRNA-synt_1c
ANCDUO_08616	tRNA-synt_1c;tRNA-synt_1c_C
ANCDUO_06892	tRNA-synt_1;Anticodon_1;tRNA-synt_1g;tRNA-synt_1_2
ANCDUO_11709	tRNA-synt_1;Anticodon_1;tRNA-synt_1g;tRNA-synt_1e
ANCDUO_06455	tRNA-synt_2b
ANCDUO_23171	tRNA-synt_2d

ANCDUO_12569	tRNA-synt_1b
ANCDUO_04992	tRNA-synt_1;Anticodon_1;tRNA-synt_1g
ANCDUO_07292	tRNA-synt_1g;tRNA-synt_1;tRNA-synt_1e;Anticodon_1
ANCDUO_13732	tRNA-synt_2;tRNA_anti-codon
ANCDUO_11991	tRNA-synt_2d
ANCDUO_25109	tRNA-synt_1
ANCDUO_26559	tRNA-synt_2b
ANCDUO_07362	tRNA-synt_1b
ANCDUO_16028	tRNA-synt_1c;tRNA_synt_1c_R2
ANCDUO_18271	tRNA-synt_2b;TGS;tRNA_SAD
ANCDUO_18384	tRNA-synt_1b
ANCDUO_24372	tRNA-synt_1g;tRNA-synt_1
ANCDUO_12570	tRNA-synt_1b
ANCDUO_26984	tRNA-synt_1g
ANCDUO_08542	tRNA-synt_1;tRNA-synt_1g
ANCDUO_01345	tRNA-synt_1;Anticodon_1;tRNA-synt_1g
ANCDUO_23741	tRNA-synt_1c;tRNA-synt_1e
ALUE_0000267201-mRNA-1	tRNA-synt_1c
ALUE_0000277801-mRNA-1	tRNA-synt_His;HGTP_anticodon;HGTP_anticodon2
ALUE_0000337301-mRNA-1	tRNA-synt_1g;tRNA_bind;NUC153;tRNA-synt_1
ALUE_0000379701-mRNA-1	tRNA-synt_1c;tRNA_synt_1c_R1;tRNA-synt_1c_C;tRNA_synt_1c_R2
ALUE_0000385201-mRNA-1	Utp14;tRNA-synt_2b;HGTP_anticodon;ProRS-C_1;WHEP-TRS
ALUE_0000390001-mRNA-1	tRNA-synt_2c;tRNA_SAD;DHHA1
ALUE_0000390801-mRNA-1	tRNA-synt_2b;HGTP_anticodon
ALUE_0000401801-mRNA-1	tRNA-synt_1;tRNA-synt_1g;Anticodon_1;tRNA-synt_1e
ALUE_0000463601-mRNA-1	tRNA-synt_1e;tRNA-synt_1g;tRNA-synt_1
ALUE_0000483701-mRNA-1	WHEP-TRS;tRNA-synt_1c;tRNA-synt_1c_C;ProRS-C_1;
	tRNA-synt_2b;HGTP_anticodon
ALUE_0000498601-mRNA-1	tRNA-synt_1;tRNA-synt_1g;Anticodon_1
ALUE_0000645401-mRNA-1	tRNA-synt_2;tRNA_anti-codon;tRNA-synt_2d
ALUE_0000681201-mRNA-1	tRNA-synt_1;tRNA-synt_1g;Anticodon_1
ALUE_0000774701-mRNA-1	HGTP_anticodon;WHEP-TRS;tRNA-synt_2b
ALUE_0000822901-mRNA-1	tRNA-synt_2d;FDX-ACB
ALUE_0000865301-mRNA-1	tRNA-synt_1c
ALUE_0000883001-mRNA-1	tRNA-synt_2;tRNA_anti-codon
ALUE_0000911501-mRNA-1	Anticodon_1;tRNA-synt_1
ALUE_0001038001-mRNA-1	tRNA-synt_1;tRNA-synt_1g
ALUE_0001048001-mRNA-1	tRNA-synt_2
ALUE_0001133801-mRNA-1	tRNA-synt_1b
ALUE_0001210501-mRNA-1	tRNA-synt_2d
ALUE_0001228401-mRNA-1	tRNA-synt_2;tRNA_anti-codon
ALUE_0001289101-mRNA-1	tRNA-synt_2b
ALUE_0001307801-mRNA-1	tRNA-synt_2b;Seryl_tRNA_N
ALUE_0001367301-mRNA-1	tRNA-synt_1b
ALUE_0001373501-mRNA-1	tRNA-synt_1b
ALUE_0001389901-mRNA-1	tRNA-synt_1;Anticodon_1;tRNA-synt_1g

ALUE_0001586901-mRNA-1	tRNA-synt_1b
ALUE_0001615801-mRNA-1	tRNA-synt_1d
ALUE_0001633301-mRNA-1	tRNA-synt_2b;HGTP_anticodon;TGS;tRNA_SAD
ALUE_0001678701-mRNA-1	tRNA-synt_2;RNase_P_Rpp14
ALUE_0001753901-mRNA-1	tRNA-synt_1g;tRNA-synt_1
ALUE_0001831201-mRNA-1	tRNA-synt_2c
ALUE_0002027701-mRNA-1	tRNA-synt_1;tRNA-synt_1g
ALUE_0002066301-mRNA-1	DALR_1;tRNA-synt_1d
ALUE_0002084501-mRNA-1	tRNA-synt_1;Anticodon_1;tRNA-synt_1g
ALUE_0002131801-mRNA-1	tRNA-synt_1d
ALUE_0002174301-mRNA-1	tRNA-synt_1d;DALR_1
ALUE_0002260301-mRNA-1	tRNA-synt_2b
ALUE_0002293401-mRNA-1	tRNA-synt_1
ALUE_0002358501-mRNA-1	tRNA-synt_1;tRNA-synt_1g
Bm10312a	tRNA-synt_2d;tRNA-synt_2
Bm10312b	tRNA-synt_2d;tRNA-synt_2
Bm10312c	tRNA-synt_2d;tRNA-synt_2
Bm10312d	tRNA-synt_2d;tRNA-synt_2
Bm11475a	ProRS-C_1;tRNA-synt_2b;HGTP_anticodon
Bm11983	tRNA-synt_2;tRNA_anti-codon
Bm12638	tRNA-synt_1e;tRNA-synt_1g
Bm12796	tRNA-synt_2;Peptidase_C1;tRNA_anti-codon
Bm13678a	tRNA-synt_2c;tRNA_SAD;DHHA1
Bm13678b	tRNA-synt_2c
Bm13726	tRNA-synt_1;Anticodon_1;tRNA-synt_1g
Bm13741	tRNA-synt_2;tRNA_anti-codon
Bm13816	tRNA-synt_1c;tRNA_synt_1c_R1;tRNA-synt_1c_C;tRNA_synt_1c_R2
Bm13878	tRNA-synt_2b;HGTP_anticodon;TGS;tRNA_SAD
Bm13920a	tRNA-synt_2;tRNA_anti-codon;tRNA-synt_2d
Bm13920b	tRNA-synt_2;tRNA_anti-codon;tRNA-synt_2d
Bm13923a	tRNA-synt_His;HGTP_anticodon;HGTP_anticodon2
Bm13923b	tRNA-synt_His;HGTP_anticodon;HGTP_anticodon2
Bm13950	tRNA-synt_2b
Bm13961a	tRNA-synt_1b
Bm13961b	tRNA-synt_1b
Bm13961c	tRNA-synt_1b
Bm13983	tRNA-synt_2d;FDX-ACB
Bm14742	tRNA-synt_1c
Bm15491	tRNA-synt_2
Bm17689	tRNA-synt_2;tRNA_anti-codon
Bm2357a	tRNA-synt_1;Anticodon_1;tRNA-synt_1g
Bm2357b	tRNA-synt_1;tRNA-synt_1g;Anticodon_1
Bm2473	tRNA-synt_1d;DALR_1
Bm2816	tRNA-synt_2b;Seryl_tRNA_N
Bm347	tRNA-synt_1c
Bm3517a	tRNA-synt_2;tRNA_anti-codon

Bm3517b	tRNA-synt_2
Bm3629a	tRNA-synt_1d;DALR_1;Arg_tRNA_synt_N
Bm3629b	tRNA-synt_1d;DALR_1;Arg_tRNA_synt_N
Bm4545	tRNA-synt_1g;tRNA_bind;tRNA-synt_1
Bm5408	tRNA-synt_1;Anticodon_1;tRNA-synt_1g;tRNA-synt_1e
Bm5713a	HGTP_anticodon;WHEP-TRS;tRNA-synt_2b
Bm5713b	HGTP_anticodon;WHEP-TRS;tRNA-synt_2b
Bm5875	tRNA-synt_1c;WHEP-TRS;tRNA-synt_1c_C;ProRS-C_1;
	HGTP_anticodon;tRNA-synt_2b
Bm6054	tRNA-synt_2b;HGTP_anticodon
Bm6985	tRNA-synt_2
Bm7157	tRNA-synt_1b
Bm7171a	tRNA-synt_1;Anticodon_1;tRNA-synt_1g;tRNA-synt_1_2
Bm7171b	tRNA-synt_1;Anticodon_1;tRNA-synt_1_2;tRNA-synt_1g
Bm7239	tRNA-synt_1b
Bm724	tRNA-synt_1b;Pex2_Pex12;zf-C3HC4
Bm7240	tRNA-synt_1g;tRNA-synt_1;tRNA-synt_1e
Bm7405	tRNA-synt_1;Anticodon_1;tRNA-synt_1g
Bm7489a	tRNA-synt_1;tRNA-synt_1g
Bm7489b	tRNA-synt_1;tRNA-synt_1g
Bm7489c	tRNA-synt_1;tRNA-synt_1g
Bm749	tRNA-synt_2;tRNA_anti-codon
Bm7700	tRNA-synt_2
Bm9427a	tRNA-synt_2c;tRNA_SAD
Bm9427b	tRNA-synt_2c;tRNA_SAD
Bm9427c	tRNA-synt_2c;tRNA_SAD
Bm9427d	tRNA-synt_2c
Bm9427e	tRNA-synt_2c
Bm9833a	tRNA-synt_2;tRNA_anti-codon
Bm9833b	tRNA-synt_2
BTMF_0000071201-mRNA-1	tRNA-synt_1;tRNA-synt_1g;Anticodon_1;tRNA-synt_1_2
BTMF_0000108701-mRNA-1	tRNA-synt_1b
BTMF_0000142801-mRNA-1	tRNA-synt_1c;tRNA-synt_1c_C;tRNA_synt_1c_R2
BTMF_0000197801-mRNA-1	tRNA-synt_2c
BTMF_0000272701-mRNA-1	tRNA-synt_1c
BTMF_0000351101-mRNA-1	tRNA-synt_1b
BTMF_0000383501-mRNA-1	tRNA-synt_1
BTMF_0000436001-mRNA-1	tRNA-synt_1
BTMF_0000481701-mRNA-1	tRNA-synt_2b
BTMF_0000547201-mRNA-1	tRNA-synt_2d;tRNA-synt_2
BTMF_0000586501-mRNA-1	tRNA-synt_1d
BTMF_0000791701-mRNA-1	tRNA-synt_His;HGTP_anticodon
BTMF_0000795301-mRNA-1	tRNA-synt_2c;tRNA_SAD;DHHA1
BTMF_0000826501-mRNA-1	tRNA-synt_1g;Ku;tRNA-synt_1;tRNA-synt_1e;Anticodon_1;Ku_C;Ku_N
BTMF_0000836201-mRNA-1	tRNA-synt_1;tRNA-synt_1g
BTMF_0000840201-mRNA-1	tRNA-synt_2

BTMF_0000852201-mRNA-1	tRNA-synt_1c;WHEP-TRS;tRNA-synt_1c_C;HGTP_anticodon ;tRNA-synt_2b;ProRS-C_1
BTMF_0000859301-mRNA-1	tRNA-synt_1g;tRNA_bind;tRNA-synt_1
BTMF_0000877601-mRNA-1	tRNA-synt_1d;DALR_1;Arg_tRNA_synt_N
BTMF_0000882501-mRNA-1	tRNA-synt_2;tRNA_anti-codon;tRNA-synt_2d
BTMF_0000917001-mRNA-1	tRNA-synt_1d
BTMF_0000924901-mRNA-1	HGTP_anticodon;WHEP-TRS;tRNA-synt_2b
BTMF_0000961301-mRNA-1	utp
BTMF_0000965501-mRNA-1	tRNA-synt_2
BTMF_0000980601-mRNA-1	tRNA-synt_2c;tRNA_SAD
BTMF_0000994401-mRNA-1	tRNA-synt_1b
BTMF_0001103301-mRNA-1	tRNA-synt_1;Anticodon_1;tRNA-synt_1g
BTMF_0001212101-mRNA-1	tRNA-synt_1;Anticodon_1;tRNA-synt_1g
BTMF_0001213901-mRNA-1	tRNA-synt_1e;tRNA-synt_1g
BTMF_0001214301-mRNA-1	tRNA-synt_1b
BTMF_0001222401-mRNA-1	tRNA-synt_2;tRNA_anti-codon
BTMF_0001243101-mRNA-1	tRNA-synt_2b
BTMF_0001326601-mRNA-1	tRNA-synt_2b;TGS;tRNA_SAD
BTMF_0001334901-mRNA-1	tRNA-synt_2d
BTMF_0001416801-mRNA-1	tRNA-synt_2d;FDX-ACB
BTMF_0001555901-mRNA-1	tRNA-synt_2b
BTMF_0001599001-mRNA-1	tRNA-synt_2c
BTMF_0001635901-mRNA-1	tRNA-synt_1;tRNA-synt_1g;tRNA-synt_1e
BTMF_0001656901-mRNA-1	tRNA-synt_1b
BTMF_0001821501-mRNA-1	tRNA-synt_2b
csin103748	tRNA-synt_1c
csin107926	tRNA-synt_2c;tRNA_SAD
csin104824	tRNA-synt_1g;tRNA-synt_1
csin113533	HGTP_anticodon;tRNA-synt_2b
csin111222	tRNA-synt_1c;tRNA-synt_1c_C;tRNA_synt_1c_R2
csin113588	tRNA-synt_1b;tRNA_bind
csin107343	tRNA-synt_1;Anticodon_1;tRNA-synt_1g
csin102069	tRNA-synt_1;Anticodon_1;tRNA-synt_1g;tRNA-synt_1e
csin100230	tRNA-synt_1b
csin109131	tRNA-synt_2;tRNA-synt_2d;tRNA_anti-codon
csin108881	tRNA-synt_His;HGTP_anticodon
csin103683	tRNA-synt_2c;tRNA_SAD;DHHA1
csin101029	tRNA-synt_1d;DALR_1;Arg_tRNA_synt_N
csin107533	tRNA-synt_2;tRNA_anti-codon
csin100392	tRNA-synt_2b;HGTP_anticodon;TGS;tRNA_SAD
csin111567	tRNA-synt_2b
csin108055	tRNA-synt_2b;Seryl_tRNA_N
csin100225	tRNA-synt_2;tRNA_anti-codon
csin106767	tRNA-synt_1e
csin113592	tRNA-synt_1b
csin106629	tRNA-synt_2

csin100211	tRNA-synt_1d
csin105292	tRNA-synt_1;Anticodon_1;tRNA-synt_1g
csin109169	tRNA-synt_2d;FDX-ACB
csin108609	tRNA-synt_1;tRNA-synt_1g
csin105286	tRNA-synt_1b
csin103200	tRNA-synt_1;Anticodon_1;tRNA-synt_1g;tRNA-synt_1_2
csin109806	tRNA-synt_2
csin112601	tRNA-synt_1g;tRNA-synt_1
csin103939	tRNA-synt_1b
csin113526	tRNA-synt_2;GAD
csin111916	TPR_11;tRNA-synt_2b;TPR_12;TPR_2;TPR_1;TPR_8;TPR_14;TPR_19;TPR_17
csin100096	tRNA-synt_1;tRNA-synt_1g;Anticodon_1;tRNA-synt_1e
csin101886	DHR-2;tRNA-synt_2
csin112273	tRNA-synt_2b
csin109224	tRNA-synt_1c;tRNA-synt_1c_C;tRNA-synt_2b
csin113519	tRNA-synt_1;tRNA-synt_1_2
csin109978	tRNA-synt_1g;Anticodon_1
csin112583	tRNA-synt_2d
DILT_0000003901-mRNA-1	tRNA-synt_1
DILT_0000330901-mRNA-1	tRNA-synt_1;tRNA-synt_1_2
DILT_0000355801-mRNA-1	tRNA-synt_2b
DILT_0000401801-mRNA-1	tRNA-synt_2c
DILT_0000408101-mRNA-1	tRNA-synt_1c
DILT_0000510201-mRNA-1	tRNA-synt_2b;WHEP-TRS
DILT_0000519501-mRNA-1	tRNA-synt_1;tRNA-synt_1g
DILT_0000546901-mRNA-1	tRNA-synt_His;HGTP_anticodon
DILT_0000585301-mRNA-1	tRNA-synt_2;tRNA_anti-codon;tRNA-synt_2d
DILT_0000621201-mRNA-1	tRNA-synt_2c
DILT_0000721001-mRNA-1	tRNA-synt_2d
DILT_0000731901-mRNA-1	tRNA-synt_1g;tRNA-synt_1
DILT_0000786001-mRNA-1	tRNA-synt_1;tRNA-synt_1g
DILT_0000798901-mRNA-1	tRNA-synt_1;tRNA-synt_1g;tRNA-synt_1_2
DILT_0000823801-mRNA-1	tRNA-synt_2
DILT_0000824101-mRNA-1	tRNA-synt_1d
DILT_0000894601-mRNA-1	tRNA-synt_1e
DILT_0000909001-mRNA-1	tRNA-synt_1b
DILT_0000913801-mRNA-1	tRNA-synt_1b
DILT_0000915901-mRNA-1	tRNA-synt_1d
DILT_0000976601-mRNA-1	tRNA-synt_2
DILT_0001210401-mRNA-1	tRNA-synt_1c
DILT_0001228301-mRNA-1	tRNA-synt_1
DILT_0001260701-mRNA-1	tRNA-synt_1
DILT_0001262101-mRNA-1	tRNA-synt_1
DILT_0001288401-mRNA-1	tRNA-synt_2
DILT_0001355001-mRNA-1	tRNA-synt_1d
DILT_0001380301-mRNA-1	tRNA-synt_1g

DILT_0001412401-mRNA-1	tRNA-synt_1d
DILT_0001415301-mRNA-1	tRNA-synt_1g
DILT_0001438101-mRNA-1	Arg_tRNA_synt_N;tRNA-synt_1d
DILT_0001480801-mRNA-1	tRNA-synt_2b
DILT_0001528001-mRNA-1	tRNA-synt_2
DILT_0001540201-mRNA-1	tRNA_anti-codon;tRNA-synt_2
DILT_0001573901-mRNA-1	tRNA-synt_1b
DILT_0001616401-mRNA-1	tRNA-synt_1c
DILT_0001690201-mRNA-1	tRNA-synt_1b
DILT_0001828001-mRNA-1	tRNA-synt_1;tRNA-synt_1g
DILT_0001855801-mRNA-1	tRNA-synt_2b
DILT_0001864201-mRNA-1	tRNA-synt_1c
DILT_0001892101-mRNA-1	tRNA-synt_1;tRNA-synt_1g
DILT_0001931501-mRNA-1	tRNA-synt_1b
DME_0000011101-mRNA-1	tRNA-synt_1;Glyco_transf_22;Anticodon_1;tRNA-synt_1g
DME_0000048201-mRNA-1	HGTP_anticodon;WHEP-TRS;tRNA-synt_2b
DME_0000057101-mRNA-1	tRNA-synt_1d;DALR_1;Arg_tRNA_synt_N
DME_0000087301-mRNA-1	tRNA-synt_2;tRNA_anti-codon
DME_0000144701-mRNA-1	tRNA-synt_1c;tRNA-synt_1c_C;tRNA_synt_1c_R1;tRNA_synt_1c_R2
DME_0000183601-mRNA-1	tRNA-synt_2d;FDX-ACB
DME_0000219401-mRNA-1	Utp14;HGTP_anticodon;tRNA-synt_2b;ProRS-C_1
DME_0000289001-mRNA-1	Met_10;tRNA-synt_2b
DME_0000296501-mRNA-1	tRNA-synt_1;Anticodon_1;tRNA-synt_1g
DME_0000302101-mRNA-1	tRNA-synt_2;tRNA_anti-codon
DME_0000322001-mRNA-1	tRNA-synt_2b;Seryl_tRNA_N
DME_0000372201-mRNA-1	tRNA-synt_1e;tRNA-synt_1g;tRNA-synt_1c
DME_0000404601-mRNA-1	tRNA-synt_2;tRNA_anti-codon
DME_0000489201-mRNA-1	tRNA-synt_1g;tRNA-synt_1;tRNA-synt_1e
DME_0000518801-mRNA-1	tRNA-synt_1b;Nt_Gln_amidase
DME_0000521701-mRNA-1	tRNA-synt_2b
DME_0000547901-mRNA-1	tRNA-synt_2c;tRNA_SAD
DME_0000550201-mRNA-1	tRNA-synt_1c;WHEP-TRS;ProRS-C_1;tRNA-synt_2b;<br>tRNA-synt_1c_C;HGTP_anticodon
DME_0000578101-mRNA-1	tRNA-synt_2c;tRNA_SAD
DME_0000629901-mRNA-1	tRNA-synt_1d;DALR_1
DME_0000654601-mRNA-1	tRNA-synt_2d
DME_0000699401-mRNA-1	tRNA-synt_1;tRNA-synt_1g
DME_0000724601-mRNA-1	tRNA-synt_1b
DME_0000729901-mRNA-1	tRNA-synt_His;HGTP_anticodon
DME_0000764501-mRNA-1	tRNA-synt_1g;tRNA_bind;tRNA-synt_1
DME_0000849701-mRNA-1	tRNA-synt_1b
DME_0000866101-mRNA-1	tRNA-synt_2;tRNA_anti-codon;tRNA-synt_2d
DME_0000866701-mRNA-1	tRNA-synt_1c
DME_0000877501-mRNA-1	tRNA-synt_1;Anticodon_1;tRNA-synt_1g
DME_0001024401-mRNA-1	tRNA-synt_1;tRNA-synt_1g;Anticodon_1;tRNA-synt_1e
DME_0001026001-mRNA-1	tRNA-synt_2b;HGTP_anticodon;TGS;tRNA_SAD

DME_0001041101-mRNA-1	tRNA-synt_1;Anticodon_1
DME_0001041301-mRNA-1	tRNA-synt_1;tRNA-synt_1_2
DME_0001068101-mRNA-1	tRNA-synt_2;tRNA_anti-codon
DME_0001070301-mRNA-1	tRNA-synt_1b
EgrG_000972000	tRNA-synt_1g;Anticodon_1;tRNA-synt_1
EgrG_000520000	tRNA-synt_1c;tRNA-synt_1c_C;ProRS-C_1;HGTP_anticodon;tRNA-synt_2b
EgrG_000976400	HGTP_anticodon;tRNA-synt_2b;WHEP-TRS
EgrG_000348100	tRNA-synt_1d;DALR_1;Arg_tRNA_synt_N
EgrG_000248300	tRNA-synt_1c;tRNA-synt_1c_C;tRNA_synt_1c_R2
EgrG_000435000	tRNA-synt_1;Anticodon_1;tRNA-synt_1g
EgrG_000104900	tRNA-synt_1;tRNA-synt_1g;Anticodon_1;tRNA-synt_1e
EgrG_000889700	tRNA-synt_2
EgrG_001147800	tRNA-synt_1b;tRNA_bind
EgrG_000239900	tRNA-synt_2b
EgrG_000186800	tRNA-synt_1g;tRNA-synt_1
EgrG_002008700	tRNA-synt_1;tRNA-synt_1g;Anticodon_1
EgrG_000461800	tRNA-synt_2b
EgrG_000861400	tRNA-synt_1;tRNA-synt_1g;Anticodon_1
EgrG_001147900	tRNA-synt_2d
EgrG_001197300	tRNA-synt_2b;Seryl_tRNA_N
EgrG_000095100	tRNA-synt_1d;DALR_1
EgrG_002026800	tRNA-synt_2;tRNA_anti-codon
EgrG_000629500	tRNA-synt_2;tRNA_anti-codon;tRNA-synt_2d
EgrG_000437500	tRNA-synt_2c;tRNA_SAD
EgrG_000932500	tRNA-synt_2d;FDX-ACB
EgrG_001008100	tRNA-synt_His;HGTP_anticodon
EgrG_000394100	tRNA-synt_1b
EgrG_000759100	tRNA-synt_1b
EgrG_000440600	tRNA-synt_2c;tRNA_SAD
EgrG_000793800	tRNA-synt_1e;tRNA-synt_1g
EgrG_000888800	tRNA-synt_1c
EgrG_000375800	tRNA-synt_2b;HGTP_anticodon;TGS;tRNA_SAD
EgrG_000348600	tRNA-synt_2;tRNA_anti-codon
EgrG_000527000	tRNA-synt_1;Anticodon_1;tRNA-synt_1g;tRNA-synt_1_2
EgrG_000754600	tRNA-synt_1;tRNA-synt_1g;tRNA-synt_1_2
EgrG_000777100	tRNA-synt_2;tRNA_anti-codon;tRNA-synt_2d
EgrG_000901500	tRNA-synt_1b
EmuJ_000095100.1	tRNA-synt_1d;DALR_1
EmuJ_000104900.1	tRNA-synt_1;tRNA-synt_1g;Anticodon_1;tRNA-synt_1e
EmuJ_000186800.1	tRNA-synt_1g;tRNA-synt_1
EmuJ_000239900.1	tRNA-synt_2b
EmuJ_000248300.1	tRNA-synt_1c;tRNA-synt_1c_C;tRNA_synt_1c_R2
EmuJ_000296500.1	tRNA-synt_1;Anticodon_1;tRNA-synt_1g
EmuJ_000348100.1	tRNA-synt_1d;DALR_1;Arg_tRNA_synt_N
EmuJ_000348300.1	tRNA-synt_2;tRNA_anti-codon
EmuJ_000348600.1	tRNA-synt_2;tRNA_anti-codon

EmuJ_000348700.1	tRNA-synt_2;tRNA_anti-codon
EmuJ_000375800.1	tRNA-synt_2b;HGTP_anticodon;TGS;tRNA_SAD
EmuJ_000394100.1	tRNA-synt_1b
EmuJ_000435000.1	tRNA-synt_1;Anticodon_1;tRNA-synt_1g
EmuJ_000437500.1	tRNA-synt_2c;tRNA_SAD;DHHA1
EmuJ_000440600.1	tRNA-synt_2c;tRNA_SAD
EmuJ_000461800.1	tRNA-synt_2b
EmuJ_000520000.1	tRNA-synt_1c;tRNA-synt_1c_C;ProRS-C_1;HGTP_anticodon;tRNA-synt_2b
EmuJ_000527000.1	tRNA-synt_1;Anticodon_1;tRNA-synt_1g;tRNA-synt_1_2
EmuJ_000629500.1	tRNA-synt_2;tRNA-synt_2d;tRNA_anti-codon
EmuJ_000754600.1	tRNA-synt_1;tRNA-synt_1g;tRNA-synt_1_2
EmuJ_000759100.1	tRNA-synt_1b
EmuJ_000777100.1	tRNA-synt_2;tRNA_anti-codon;tRNA-synt_2d
EmuJ_000793800.1	tRNA-synt_1e;tRNA-synt_1g
EmuJ_000861400.1	tRNA-synt_1;tRNA-synt_1g;Anticodon_1
EmuJ_000888800.1	tRNA-synt_1c
EmuJ_000889700.1	tRNA-synt_2
EmuJ_000901500.1	tRNA-synt_1b
EmuJ_000932500.1	tRNA-synt_2d;FDX-ACB
EmuJ_000972000.1	tRNA-synt_1g;Anticodon_1;tRNA-synt_1
EmuJ_000976400.1	HGTP_anticodon;tRNA-synt_2b;WHEP-TRS
EmuJ_001008100.1	tRNA-synt_His;HGTP_anticodon
EmuJ_001147800.1	tRNA-synt_1b;tRNA_bind
EmuJ_001147900.1	tRNA-synt_2d
EmuJ_001197300.1	tRNA-synt_2b;Seryl_tRNA_N
EmuJ_002101900.1	tRNA-synt_2
D915_15485	tRNA-synt_1c
D915_07668	tRNA-synt_2d
D915_12728	tRNA-synt_2;tRNA-synt_2d
D915_04166	tRNA-synt_1g
D915_12771	tRNA-synt_1b
D915_15934	tRNA-synt_2c
D915_03300	tRNA-synt_1e
D915_10078	tRNA-synt_1c
D915_04073	tRNA-synt_1c
D915_00715	tRNA-synt_1e;tRNA-synt_1g
D915_14857	tRNA-synt_2d
D915_15471	tRNA-synt_2;GAD;tRNA_anti-codon
D915_02816	tRNA-synt_His;HGTP_anticodon
D915_13996	tRNA-synt_1;tRNA-synt_1g
D915_14758	tRNA-synt_2c
D915_12769	tRNA-synt_1b
D915_15382	tRNA-synt_1;tRNA-synt_1g
D915_03274	tRNA_bind;tRNA-synt_1b
D915_07610	tRNA-synt_1b
D915_12414	tRNA-synt_1

D915_11237	tRNA-synt_2b
D915_02080	tRNA-synt_1
D915_09139	tRNA-synt_2
D915_15002	SHMT;CPSase_L_D2;tRNA-synt_1b;tRNA-synt_2d;
D915_01628	tRNA-synt_2c
D915_06994	tRNA-synt_2;tRNA_anti-codon
D915_14311	tRNA-synt_1;tRNA-synt_1_2;tRNA-synt_1g;Anticodon_1
	;tRNA-synt_1e;tRNA-synt_1f
D915_03166	tRNA-synt_1d;DALR_1
D915_15509	tRNA-synt_2b;Seryl_tRNA_N
D915_15939	tRNA-synt_1
D915_10079	tRNA-synt_1c;tRNA_synt_1c_R2
D915_15358	tRNA-synt_1f;GTP_EFTU;LepA_C;EFG_C;GTP_EFTU_D2;EFG_II;MMR_HSR1
D915_00713	tRNA-synt_1
D915_06032	tRNA-synt_2
D915_13998	tRNA-synt_1
D915_15897	tRNA-synt_2c
D915_10077	tRNA-synt_1c_C;tRNA-synt_1c
D915_15896	tRNA-synt_2c
D915_13975	tRNA-synt_1c
D915_12770	tRNA-synt_1b
D915_14856	tRNA-synt_2d
D915_03348	tRNA-synt_1b
D915_14106	tRNA-synt_1g;tRNA-synt_1
D915_14414	tRNA-synt_1;tRNA-synt_1g
D915_13997	tRNA-synt_1
D915_04076	tRNA-synt_2b;HGTP_anticodon;ProRS-C_1
D915_06033	tRNA-synt_2
D915_14415	tRNA-synt_1
D915_09784	tRNA-synt_1;Anticodon_1;tRNA-synt_1_2;tRNA-synt_1g
D915_03301	tRNA-synt_1e
D915_02817	tRNA-synt_His
D915_14757	tRNA-synt_2c
D915_02081	tRNA-synt_1;tRNA-synt_1g
D915_13096	tRNA-synt_2b
D915_15435	tRNA-synt_1;Anticodon_1;tRNA-synt_1g;tRNA-synt_1_2
D915_15547	tRNA-synt_2c
D915_06401	tRNA-synt_2b
D915_01627	tRNA-synt_2c;tRNA_SAD;DHHA1
D915_12727	tRNA-synt_2
D915_14824	HSP70;tRNA-synt_2b;HGTP_anticodon;tRNA_SAD
D915_15606	tRNA-synt_1c
D915_15245	tRNA-synt_1b;S4
D915_04614	tRNA-synt_2b
D915_15568	DALR_1;tRNA-synt_1d;Arg_tRNA_synt_N;tRNA-synt_1g
D915_04074	tRNA-synt_1c;tRNA-synt_1c_C

D915_06993	tRNA-synt_2
D915_15261	tRNA-synt_1g;tRNA-synt_1;tRNA-synt_1e
D915_15252	tRNA-synt_1e;tRNA-synt_1g;DALR_2;tRNA-synt_1;tRNA-synt_1f
D915_15589	tRNA-synt_1;Pribosyltran;tRNA-synt_1g
HDID_0000011001-mRNA-1	tRNA-synt_1;Anticodon_1;tRNA-synt_1g;tRNA-synt_1_2
HDID_0000014201-mRNA-1	tRNA-synt_2c
HDID_0000036901-mRNA-1	tRNA-synt_1b
HDID_0000058101-mRNA-1	tRNA-synt_1c;tRNA-synt_1c_C;tRNA_synt_1c_R2
HDID_0000075901-mRNA-1	tRNA-synt_1c
HDID_0000128801-mRNA-1	tRNA-synt_2b
HDID_0000131401-mRNA-1	tRNA-synt_2;tRNA_anti-codon
HDID_0000159301-mRNA-1	HGTP_anticodon;WHEP-TRS;tRNA-synt_2b
HDID_0000196101-mRNA-1	tRNA-synt_1b;ANAPC4;ANAPC4_WD40
HDID_0000228001-mRNA-1	tRNA-synt_1d;DALR_1
HDID_0000271901-mRNA-1	tRNA-synt_1c;tRNA-synt_1c_C;ProRS-C_1;tRNA-synt_2b;HGTP_anticodon
HDID_0000312701-mRNA-1	tRNA-synt_2c
HDID_0000353601-mRNA-1	tRNA-synt_1;tRNA-synt_1g
HDID_0000354901-mRNA-1	tRNA-synt_1;tRNA-synt_1g
HDID_0000365001-mRNA-1	tRNA-synt_His;HGTP_anticodon
HDID_0000391101-mRNA-1	tRNA-synt_1c
HDID_0000416601-mRNA-1	tRNA-synt_2b;Seryl_tRNA_N
HDID_0000453901-mRNA-1	tRNA_SAD;tRNA-synt_2c;DHHA1
HDID_0000478501-mRNA-1	tRNA-synt_2;tRNA_anti-codon;tRNA-synt_2d
HDID_0000521801-mRNA-1	tRNA-synt_2
HDID_0000528501-mRNA-1	tRNA-synt_2b;TGS;tRNA_SAD;HGTP_anticodon
HDID_0000609801-mRNA-1	tRNA-synt_1;tRNA-synt_1g
HDID_0000675801-mRNA-1	tRNA-synt_2;tRNA_anti-codon
HDID_0000676001-mRNA-1	tRNA-synt_1d;DALR_1;Arg_tRNA_synt_N
HDID_0000704501-mRNA-1	tRNA-synt_1;tRNA-synt_1g;Anticodon_1;tRNA-synt_1e
HDID_0000722401-mRNA-1	tRNA-synt_2b;TPR_11;TPR_8;TPR_17
HDID_0000748501-mRNA-1	tRNA-synt_1b
HDID_0000758901-mRNA-1	tRNA-synt_1g;tRNA-synt_1;tRNA-synt_1e
HDID_0000791701-mRNA-1	tRNA-synt_2c;tRNA_SAD
HDID_0000848501-mRNA-1	tRNA-synt_1g;Anticodon_1;tRNA-synt_1
HDID_0000875201-mRNA-1	tRNA-synt_1b;tRNA_bind
HDID_0000929601-mRNA-1	tRNA-synt_2d
HDID_0000951301-mRNA-1	tRNA-synt_1e;tRNA-synt_1g
HDID_0000985301-mRNA-1	tRNA-synt_2;tRNA_anti-codon;tRNA-synt_2d
HDID_0001039201-mRNA-1	tRNA-synt_1;Anticodon_1;tRNA-synt_1g
HDID_0001060701-mRNA-1	tRNA-synt_1;tRNA-synt_1g
HDID_0001090001-mRNA-1	tRNA-synt_2d;FDX-ACB
HDID_0001090801-mRNA-1	tRNA-synt_1;Anticodon_1;tRNA-synt_1g
HmN_000000100.1	tRNA-synt_1b;tRNA_bind
HmN_000043900.1	tRNA-synt_2;tRNA_anti-codon;tRNA-synt_2d
HmN_000055600.1	tRNA-synt_1;Anticodon_1;tRNA-synt_1g;tRNA-synt_1_2
HmN_000057000.1	tRNA-synt_1c;tRNA-synt_1c_C;ProRS-C_1;tRNA-synt_2b;HGTP_anticodon;

HmN_000119700.1	tRNA-synt_1g;Anticodon_1;tRNA-synt_1
HmN_000147200.1	tRNA-synt_1g;tRNA-synt_1;tRNA-synt_1e
HmN_000152500.1	tRNA-synt_1d
HmN_000161600.1	tRNA-synt_His;HGTP_anticodon
HmN_000166200.1	tRNA-synt_2b
HmN_000177600.1	tRNA-synt_1;Anticodon_1;tRNA-synt_1g
HmN_000195600.1	tRNA-synt_2b;Seryl_tRNA_N
HmN_000326900.1	tRNA-synt_1d;DALR_1;Arg_tRNA_synt_N
HmN_000327000.1	tRNA-synt_2;tRNA_anti-codon
HmN_000327100.1	tRNA-synt_2
HmN_000368500.1	tRNA-synt_2b;HGTP_anticodon;TGS;tRNA_SAD
HmN_000369000.1	HGTP_anticodon;tRNA-synt_2b;WHEP-TRS
HmN_000434500.1	tRNA-synt_1e;tRNA-synt_1g
HmN_000450800.1	tRNA-synt_2d;FDX-ACB
HmN_000458500.1	tRNA-synt_2
HmN_000470300.1	tRNA-synt_1c;tRNA-synt_1c_C;tRNA_synt_1c_R2
HmN_000495200.1	tRNA-synt_2c;tRNA_SAD
HmN_000496200.1	tRNA-synt_1;Glyco_hydro_85;Anticodon_1;Ribosomal_L17;tRNA-synt_1g
HmN_000637600.1	tRNA-synt_1b
HmN_000648700.1	tRNA-synt_1b
HmN_000660100.1	tRNA-synt_2b
HmN_000729500.1	tRNA-synt_2c;tRNA_SAD
HmN_000753800.1	tRNA-synt_1;tRNA-synt_1g
HmN_000830300.1	tRNA-synt_1;Anticodon_1;tRNA-synt_1g
HmN_000852000.1	tRNA-synt_1b
HmN_002144100.1	tRNA-synt_1c
HmN_002145200.1	tRNA-synt_2
HmN_002193200.1	tRNA-synt_2;tRNA_anti-codon;tRNA-synt_2d
HmN_002221500.1	tRNA-synt_2d
HNAJ_0000079901-mRNA-1	tRNA-synt_1
HNAJ_0000141301-mRNA-1	tRNA-synt_2b;Seryl_tRNA_N
HNAJ_0000187501-mRNA-1	tRNA-synt_1c
HNAJ_0000212901-mRNA-1	Anticodon_1;tRNA-synt_1;tRNA-synt_1g;tRNA-synt_1e
HNAJ_0000248201-mRNA-1	tRNA-synt_2b;HGTP_anticodon;TGS;tRNA_SAD
HNAJ_0000269001-mRNA-1	tRNA-synt_2c;tRNA_SAD
HNAJ_0000272201-mRNA-1	tRNA-synt_1e;tRNA-synt_1g
HNAJ_0000308801-mRNA-1	tRNA-synt_1;tRNA-synt_1g
HNAJ_0000308901-mRNA-1	tRNA-synt_1d;DALR_1;Arg_tRNA_synt_N
HNAJ_0000309001-mRNA-1	tRNA-synt_2;tRNA_anti-codon
HNAJ_0000362001-mRNA-1	tRNA-synt_2;tRNA_anti-codon
HNAJ_0000363601-mRNA-1	tRNA-synt_1b
HNAJ_0000370501-mRNA-1	tRNA-synt_2;tRNA_anti-codon;tRNA-synt_2d
HNAJ_0000374601-mRNA-1	WHEP-TRS;tRNA-synt_2b
HNAJ_0000392801-mRNA-1	tRNA-synt_1;Anticodon_1
HNAJ_0000395401-mRNA-1	tRNA-synt_2c
HNAJ_0000466301-mRNA-1	tRNA-synt_1c;tRNA_synt_1c_R2

HNAJ_0000513201-mRNA-1	tRNA-synt_2d
HNAJ_0000548601-mRNA-1	tRNA-synt_2;tRNA-synt_2d;tRNA_anti-codon
HNAJ_0000612001-mRNA-1	tRNA-synt_1g
HNAJ_0000717901-mRNA-1	tRNA-synt_2c;tRNA_SAD
HNAJ_0000748001-mRNA-1	tRNA-synt_2
HNAJ_0000782901-mRNA-1	tRNA-synt_2d
HNAJ_0000783201-mRNA-1	tRNA-synt_1b;tRNA_bind
HNAJ_0000882001-mRNA-1	tRNA-synt_1b
HNAJ_0000884601-mRNA-1	tRNA-synt_1d
HNAJ_0000906401-mRNA-1	tRNA-synt_1c;tRNA-synt_1c_C;ProRS-C_1;tRNA-synt_2b;HGTP_anticodon;
HNAJ_0000919601-mRNA-1	tRNA-synt_1;tRNA-synt_1g;Anticodon_1
HNAJ_0000996701-mRNA-1	tRNA-synt_1g;Anticodon_1;tRNA-synt_1
HNAJ_0001038901-mRNA-1	tRNA-synt_1g;tRNA-synt_1
HNAJ_0001051501-mRNA-1	tRNA-synt_1;tRNA-synt_1g
HNAJ_0001056101-mRNA-1	tRNA-synt_2b
HNAJ_0001103801-mRNA-1	tRNA-synt_His;HGTP_anticodon
HNAJ_0001113701-mRNA-1	tRNA-synt_1;Anticodon_1;tRNA-synt_1g;tRNA-synt_1_2
HNAJ_0001188701-mRNA-1	tRNA-synt_2
HNAJ_0001205801-mRNA-1	tRNA-synt_1;Anticodon_1;tRNA-synt_1g
HNAJ_0001272801-mRNA-1	tRNA-synt_1c_C;tRNA-synt_1c
HNAJ_0001358301-mRNA-1	tRNA-synt_2;tRNA_anti-codon;tRNA_anti_2;
EN70_1	tRNA-synt_1g;Methyltransf_11
EN70_1014	tRNA-synt_1c
EN70_10305	tRNA-synt_2
EN70_10426	tRNA-synt_1c;tRNA_synt_1c_R1;tRNA_synt_1c_R2;tRNA-synt_1c_C
EN70_10543	tRNA-synt_2b;HGTP_anticodon;TGS;tRNA_SAD
EN70_10784	tRNA-synt_2c;tRNA_SAD
EN70_11050	tRNA-synt_1g;tRNA_bind;tRNA-synt_1
EN70_1212	tRNA-synt_1d;DALR_1
EN70_12146	tRNA-synt_1e;tRNA-synt_1g
EN70_1252	tRNA-synt_2c;tRNA_SAD;DHHA1
EN70_1258	tRNA-synt_1;tRNA-synt_1g
EN70_283	tRNA-synt_2b
EN70_3457	tRNA-synt_1b;Pex2_Pex12
EN70_3516	tRNA-synt_2;tRNA_anti-codon
EN70_3530	tRNA-synt_2b;Seryl_tRNA_N
EN70_4466	tRNA-synt_1g;tRNA-synt_1;Methyltransf_11;Anticodon_1;
	tRNA-synt_1e;Ubie_methyltran
EN70_4745	WHEP-TRS;ProRS-C_1;tRNA-synt_1c_C;tRNA-synt_2b;HGTP_anticodon
EN70_4746	tRNA-synt_1c;tRNA-synt_1e
EN70_4912	tRNA-synt_1c
EN70_5052	tRNA-synt_2;tRNA_anti-codon;tRNA-synt_2d
EN70_5058	tRNA-synt_2d Rhomboid;tRNA-synt_2
EN70_5214	tRNA-synt_2;tRNA_anti-codon
EN70_5331	tRNA-synt_1b
EN70_5345	tRNA-synt_2

EN70_5346	tRNA-synt_2
EN70_5411	tRNA-synt_1b
EN70_5799	tRNA-synt_1;Anticodon_1;tRNA-synt_1g;tRNA-synt_1_2
EN70_5800	tRNA-synt_2d;FDX-ACB
EN70_6631	HGTP_anticodon;WHEP-TRS;tRNA-synt_2b
EN70_6707	tRNA-synt_1;Anticodon_1;tRNA-synt_1g
EN70_7547	tRNA-synt_2b;HGTP_anticodon
EN70_7700	tRNA-synt_1;Anticodon_1;tRNA-synt_1g
EN70_8096	Utp14;tRNA-synt_2b;HGTP_anticodon;ProRS-C_1
EN70_8395	Nt_Gln_amidase;tRNA-synt_1b
EN70_8755	tRNA-synt_1;Anticodon_1;tRNA-synt_1g;tRNA-synt_1_2
EN70_8849	tRNA-synt_His;HGTP_anticodon;HGTP_anticodon2;tRNA-synt_2
EN70_9796	Mito_carr;tRNA-synt_1;tRNA-synt_1g;Anticodon_1;tRNA-synt_1e
EN70_9908	tRNA-synt_1d;Arg_tRNA_synt_N;DALR_1
NECAME_00719	tRNA-synt_2c;MRP-S23;tRNA_SAD;DHHA1
NECAME_00844	tRNA-synt_1;Anticodon_1;tRNA-synt_1g
NECAME_00891	HGTP_anticodon;WHEP-TRS;tRNA-synt_2b
NECAME_01037	Proteasome;HGTP_anticodon;tRNA-synt_2b;TPR_11;WHEP-TRS;
	Proteasome_A_N;TPR_14;TPR_16;TPR_17;TPR_1;TPR_2;TPR_19;TPR_8
NECAME_01128	tRNA-synt_2b;HGTP_anticodon;TGS;tRNA_SAD
NECAME_01233	tRNA-synt_2d;FDX-ACB
NECAME_02085	tRNA-synt_1c;tRNA_synt_1c_R1;tRNA_synt_1c_R2;tRNA-synt_1c_C
NECAME_02629	tRNA-synt_2b
NECAME_03481	tRNA-synt_1d;DALR_1;Arg_tRNA_synt_N
NECAME_03562	tRNA-synt_His;HGTP_anticodon;RRM_1;RRM_5
NECAME_03860	tRNA-synt_1c
NECAME_04406	tRNA-synt_2d;tRNA-synt_2
NECAME_05073	tRNA-synt_2b;Seryl_tRNA_N
NECAME_05692	Anticodon_1;tRNA-synt_1
NECAME_05693	tRNA-synt_1
NECAME_06000	tRNA-synt_2
NECAME_06002	HGTP_anticodon;tRNA-synt_2b
NECAME_06303	tRNA-synt_1c;WHEP-TRS;tRNA-synt_1c_C
NECAME_06916	tRNA-synt_1b
NECAME_07680	tRNA-synt_1;tRNA-synt_1g;PAC3
NECAME_08865	tRNA-synt_1d;DALR_1
NECAME_09221	tRNA-synt_1e;tRNA-synt_1g
NECAME_10241	tRNA-synt_1b
NECAME_10242	tRNA-synt_1b
NECAME_10420	tRNA-synt_2c;tRNA_SAD
NECAME_10498	tRNA-synt_1;Anticodon_1;tRNA-synt_1g;tRNA-synt_1_2
NECAME_11083	tRNA-synt_2
NECAME_11885	tRNA-synt_2;tRNA_anti-codon;tRNA-synt_2d
NECAME_11931	tRNA-synt_1;Anticodon_1;tRNA-synt_1g
	tRNA-synt_1g;tRNA_bind;tRNA-synt_1;NUC153
NECAME_14660	tRNA-synt_2b

NECAME_15187	tRNA-synt_2;Glyco_hydro_47;tRNA_anti-codon
NECAME_15188	tRNA-synt_2
NECAME_16052	tRNA-synt_1b
NECAME_16192	tRNA-synt_1b
NECAME_16774	tRNA-synt_1g;tRNA-synt_1;tRNA-synt_1e
NECAME_16854	tRNA-synt_2;tRNA_anti-codon
NECAME_17299	tRNA-synt_2
NECAME_18683	tRNA-synt_1;tRNA-synt_1g
NECAME_19565	tRNA-synt_2
OVOC10390	tRNA-synt_2c;tRNA_SAD
OVOC10793	tRNA-synt_1d;DALR_1
OVOC11137	tRNA-synt_His;HGTP_anticodon
OVOC11195	tRNA-synt_1c;
OVOC11365a	tRNA-synt_1c;WHEP-TRS;tRNA-synt_1c_C;ProRS-C_1;
	HGTP_anticodon;tRNA-synt_2b
OVOC11365b	WHEP-TRS;ProRS-C_1;HGTP_anticodon;tRNA-synt_2b
OVOC11631	tRNA-synt_1g;tRNA_bind;tRNA-synt_1
OVOC1179	tRNA-synt_2b;HGTP_anticodon
OVOC11842	ProRS-C_1;HGTP_anticodon;tRNA-synt_2b
OVOC13489	tRNA-synt_1;Anticodon_1;tRNA-synt_1g;tRNA-synt_1_2
OVOC1573	tRNA-synt_1;tRNA-synt_1g
OVOC1585	tRNA-synt_2c;tRNA_SAD;DHHA1
OVOC193	tRNA-synt_1e;tRNA-synt_1g
OVOC2324	tRNA-synt_1c
OVOC2471	tRNA-synt_2b;HGTP_anticodon;TGS;tRNA_SAD
OVOC2562	tRNA-synt_2d;tRNA-synt_2
OVOC2569a	tRNA-synt_2;tRNA_anti-codon;tRNA-synt_2d
OVOC2569b	tRNA-synt_2;tRNA_anti-codon;tRNA-synt_2d
OVOC2679	tRNA-synt_1e
OVOC3179	tRNA-synt_1g;tRNA-synt_1;Anticodon_1
OVOC459	tRNA-synt_1;Anticodon_1;tRNA-synt_1g
OVOC4642	Nt_Gln_amidase;tRNA-synt_1b
OVOC5077	tRNA-synt_2b
OVOC6300	tRNA-synt_1;tRNA-synt_1g
OVOC6356	tRNA-synt_1b
OVOC6432	tRNA-synt_2
OVOC6455	tRNA-synt_1b
OVOC6597	tRNA-synt_2;tRNA_anti-codon
OVOC6599	tRNA-synt_2;tRNA_anti-codon
OVOC7102	tRNA-synt_2d;FDX-ACB
OVOC7103	tRNA-synt_1;Anticodon_1;tRNA-synt_1g
OVOC8035	tRNA-synt_1;tRNA-synt_1g;Anticodon_1;tRNA-synt_1e
OVOC820	tRNA-synt_1;Anticodon_1;tRNA-synt_1g;
OVOC8625	tRNA-synt_1b;Pex2_Pex12
OVOC8700	tRNA-synt_2;tRNA_anti-codon
OVOC8714	tRNA-synt_2b;Seryl_tRNA_N

OVOC8719	tRNA-synt_2b;Seryl_tRNA_N
OVOC8962	HGTP_anticodon;WHEP-TRS;tRNA-synt_2b
OVOC9040	tRNA-synt_2
OVOC9151	tRNA-synt_1d;DALR_1;Arg_tRNA_synt_N
OVOC9483	tRNA-synt_1c;tRNA_synt_1c_R1;tRNA-synt_1c_C;tRNA_synt_1c_R2
T265_14558	tRNA-synt_2b
T265_08843	tRNA-synt_1;tRNA-synt_1g;Anticodon_1
T265_08946	tRNA-synt_2;tRNA-synt_2d;tRNA_anti-codon
T265_09084	tRNA-synt_1b
T265_14849	tRNA-synt_2;tRNA_anti-codon
T265_09842	tRNA-synt_1e
T265_10380	tRNA-synt_2c;tRNA_SAD;DHHA1
T265_01087	tRNA-synt_2b;HGTP_anticodon;TGS;tRNA_SAD
T265_11299	tRNA-synt_1b;tRNA_bind
T265_11386	tRNA-synt_1;Anticodon_1;tRNA-synt_1g;tRNA-synt_1e
T265_11659	tRNA-synt_1d;DALR_1
T265_15554	tRNA-synt_1b
T265_15582	tRNA-synt_1;Glyco_hydro_85;tRNA-synt_1g;Anticodon_1
T265_01274	tRNA-synt_1;tRNA-synt_1g
T265_16294	tRNA-synt_1
T265_02630	tRNA-synt_1c;tRNA-synt_1c_C;ProRS-C_1;tRNA-synt_2b;HGTP_anticodon
T265_02835	tRNA-synt_2b;Seryl_tRNA_N
T265_03456	tRNA-synt_2c;tRNA_SAD
T265_03819	tRNA-synt_1d;DALR_1;Arg_tRNA_synt_N
T265_03894	tRNA-synt_2b;TPR_11;TPR_2;TPR_12;TPR_8;TPR_1;TPR_14;TPR_16;TPR_9
T265_04925	tRNA-synt_His;HGTP_anticodon
T265_05270	tRNA-synt_1b
T265_05275	tRNA-synt_2;tRNA_anti-codon
T265_13744	tRNA-synt_2;tRNA_anti-codon
T265_13748	tRNA-synt_1;Anticodon_1;tRNA-synt_1g;tRNA-synt_1_2;tRNA-synt_1e
T265_13749	tRNA-synt_1;tRNA-synt_1g
T265_05757	tRNA-synt_1g
T265_13851	tRNA-synt_1g;tRNA-synt_1
T265_13902	tRNA-synt_2;tRNA-synt_2d
T265_00610	HGTP_anticodon;WHEP-TRS;tRNA-synt_2b
T265_06528	tRNA-synt_1c;tRNA-synt_1c_C;tRNA_synt_1c_R2
T265_07520	tRNA-synt_1c
T265_14369	tRNA-synt_2d;FDX-ACB
T265_07791	tRNA-synt_1g;Anticodon_1;tRNA-synt_1
T265_07943	tRNA-synt_1;Anticodon_1;tRNA-synt_1g;tRNA-synt_1e
A_01599	tRNA-synt_2b
A_04057	tRNA-synt_2b;HGTP_anticodon;TGS;tRNA_SAD
A_07093	tRNA-synt_1c
A_01184	tRNA-synt_1;Anticodon_1;tRNA-synt_1g;tRNA-synt_1_2
A_02141	tRNA-synt_His;HGTP_anticodon
A_07424	tRNA-synt_1;Anticodon_1;tRNA-synt_1g;tRNA-synt_1_2

B_00613	tRNA-synt_2
A_02332	tRNA-synt_2d
A_08080	tRNA-synt_1;Anticodon_1;tRNA-synt_1g
A_03316	tRNA-synt_1;tRNA-synt_1g
C_00986	tRNA-synt_1c
A_06086	tRNA-synt_1g;Anticodon_1;tRNA-synt_1
A_07337	tRNA-synt_1b
A_00749	tRNA-synt_2b
B_00444	tRNA-synt_1e
B_00258	TBCC;tRNA-synt_1g;tRNA-synt_1e
A_01043	tRNA-synt_1;tRNA-synt_1g
A_00384	tRNA-synt_2c
A_03318	tRNA-synt_1
A_08373	tRNA-synt_1d;DALR_1;Arg_tRNA_synt_N
A_04059	tRNA-synt_1d;DALR_1
A_00552	tRNA-synt_2c;tRNA_SAD;DHHA1
A_06406	tRNA-synt_1g;tRNA-synt_1
A_01273	tRNA-synt_1b
A_02925	tRNA-synt_2;tRNA_anti-codon;tRNA-synt_2d
A_04779	tRNA-synt_2;tRNA_anti-codon
B_00823	tRNA-synt_2
A_02305	tRNA-synt_2d;FDX-ACB
A_00703	tRNA-synt_1b
A_06665	tRNA-synt_1c;tRNA-synt_1c_C;tRNA_synt_1c_R2
B_00054	tRNA-synt_1b
A_04168	tRNA-synt_2;tRNA_anti-codon
A_07113	HGTP_anticodon;tRNA-synt_2b;WHEP-TRS
B_00563	tRNA-synt_2;tRNA-synt_2d
A_08183	tRNA-synt_1b;tRNA_bind
B_00498	tRNA-synt_1b
A_07985	tRNA-synt_1c;tRNA-synt_1c_C;ProRS-C_1;tRNA-synt_2b;HGTP_anticodon
Sjp_0009980	tRNA-synt_1;tRNA-synt_1g;tRNA-synt_1_2
Sjp_0056410	tRNA-synt_1;tRNA-synt_1g
Sjp_0021140	tRNA-synt_1c;tRNA-synt_1c_C;tRNA_synt_1c_R2
Sjp_0072210	tRNA-synt_2
Sjp_0072220	tRNA-synt_2
Sjp_0037370	tRNA-synt_1g;tRNA-synt_1
Sjp_0047670	tRNA_bind;tRNA-synt_1b
Sjp_0122730	tRNA-synt_1e;tRNA-synt_1g
Sjp_0054640	tRNA-synt_1c;tRNA-synt_1c_C;tRNA-synt_2b;HGTP_anticodon;ProRS-C_1
Sjp_0010800	tRNA-synt_2;tRNA_anti-codon;tRNA-synt_2d
Sjp_0105270	tRNA-synt_1g;tRNA-synt_1
Sjp_0068870	tRNA-synt_1;Anticodon_1
Sjp_0050100	tRNA-synt_1;tRNA-synt_1g;Anticodon_1;tRNA-synt_1e
Sjp_0075820	tRNA-synt_2b;TPR_12;TPR_11;TPR_8;TPR_2;TPR_1
Sjp_0124150	tRNA-synt_2c

Sjp_0088910	tRNA_SAD;tRNA-synt_2c;DHHA1
Sjp_0085650	tRNA-synt_1d;DALR_1;tRNA-synt_1e
Sjp_0077090	tRNA-synt_1
Sjp_0072860	tRNA-synt_1b
Sjp_0038640	tRNA-synt_1d;DALR_1;Arg_tRNA_synt_N
Sjp_0037460	tRNA-synt_2;HlyIII;TMEM219
Sjp_0052070	tRNA-synt_2b;Seryl_tRNA_N
Sjp_0133030	tRNA-synt_1b
Sjp_0092060	tRNA-synt_1b
Sjp_0051950	tRNA-synt_2;tRNA_anti-codon
Sjp_0075600	tRNA-synt_2c
Sjp_0053670	tRNA-synt_2
Sjp_0007710	tRNA-synt_His;HGTP_anticodon
Sjp_0043850	tRNA-synt_2;tRNA-synt_2d
Sjp_0106740	tRNA-synt_1;tRNA-synt_1g
Sjp_0080790	tRNA-synt_2d
Sjp_0105480	tRNA-synt_1;Anticodon_1
Sjp_0002700	tRNA-synt_1;Anticodon_1;tRNA-synt_1g;tRNA-synt_1_2
Sjp_0066020	tRNA-synt_1c
Sjp_0061760	tRNA-synt_2c
Sjp_0013140	tRNA-synt_1;Anticodon_1;tRNA-synt_1g
Sjp_0030360	tRNA-synt_1b
Sjp_0117790	tRNA-synt_2b
Smp_005330.1	tRNA-synt_1g;tRNA-synt_1
Smp_009520.1	tRNA-synt_1c
Smp_023360.1	tRNA-synt_His;HGTP_anticodon;tRNA-synt_2b
Smp_023360.2	tRNA-synt_His;HGTP_anticodon;tRNA-synt_2b
Smp_025260.1	tRNA-synt_2b
Smp_038240.1	tRNA-synt_2c;tRNA_SAD;DHHA1
Smp_040770.1	tRNA-synt_1g;Anticodon_1;tRNA-synt_1
Smp_040800.1	HGTP_anticodon;tRNA-synt_2b;WHEP-TRS
Smp_041450.1	tRNA-synt_2;tRNA_anti-codon
Smp_041600.1	tRNA-synt_1;Anticodon_1;tRNA-synt_1g;tRNA-synt_1e
Smp_053510.1	tRNA-synt_2;tRNA_anti-codon;tRNA-synt_2d
Smp_053510.2	tRNA-synt_2;tRNA_anti-codon
Smp_053610.1	tRNA-synt_2b;Seryl_tRNA_N
Smp_057230.1	tRNA-synt_2b
Smp_082860.1	tRNA-synt_1b
Smp_088800.1	tRNA-synt_2;tRNA_anti-codon
Smp_096270.1	tRNA-synt_1;Anticodon_1;tRNA-synt_1g;tRNA-synt_1_2
Smp_097590.1	tRNA-synt_1;Anticodon_1;tRNA-synt_1g;tRNA-synt_1_2
Smp_101230.1	tRNA-synt_2d
Smp_101240.1	tRNA-synt_2d
Smp_104470.1	tRNA-synt_2;tRNA_anti-codon;tRNA-synt_2d
Smp_104470.2	tRNA-synt_2;tRNA_anti-codon;tRNA-synt_2d
Smp_122780.1	tRNA-synt_1;tRNA-synt_1g

Smp_129210.1	tRNA-synt_1b;tRNA_bind
Smp_129650.1	tRNA-synt_1;tRNA-synt_1g;Anticodon_1
Smp_133280.1	tRNA-synt_1d;DALR_1;Arg_tRNA_synt_N
Smp_135400.1	tRNA-synt_2c
Smp_138930.1	tRNA-synt_1c;tRNA-synt_1c_C;ProRS-C_1;tRNA-synt_2b;HGTP_anticodon
Smp_138930.2	tRNA-synt_1c;tRNA-synt_1c_C;ProRS-C_1;tRNA-synt_2b;HGTP_anticodon
Smp_148050.1	tRNA-synt_1c;tRNA_synt_1c_R2;tRNA-synt_1c_C
Smp_149430.1	tRNA-synt_2d;FDX-ACB
Smp_150680.1	tRNA-synt_1b
Smp_153430.1	tRNA-synt_1d;DALR_1
Smp_169030.1	tRNA-synt_2
Smp_170800.1	tRNA-synt_1e;tRNA-synt_1g
Smp_170800.2	tRNA-synt_1e;tRNA-synt_1g
Smp_176290.1	tRNA-synt_1b
Smp_179140.1	tRNA-synt_2;tRNA_anti-codon
Smp_190720.1	tRNA-synt_2
Smp_194140.1	tRNA-synt_2
Smp_194150.1	tRNA_anti-codon;tRNA-synt_2
Smp_194160.1	tRNA-synt_1;tRNA-synt_1g;Anticodon_1;tRNA-synt_1e
Smp_199340.1	tRNA-synt_2;tRNA_anti-codon
SSTP_0000084000.1	tRNA-synt_2;tRNA_anti-codon;tRNA-synt_2d
SSTP_0000088600.1	tRNA-synt_2b;HGTP_anticodon;TGS;tRNA_SAD
SSTP_0000150200.1	tRNA-synt_2
SSTP_0000197400.1	HGTP_anticodon;tRNA-synt_2b;WHEP-TRS
SSTP_0000213200.1	tRNA-synt_2d;tRNA-synt_2
SSTP_0000230400.1	tRNA-synt_1c
SSTP_0000296300.1	tRNA-synt_1;tRNA-synt_1g;Anticodon_1
SSTP_0000322900.1	tRNA-synt_1d;DALR_1
SSTP_0000337100.1	tRNA-synt_His;HGTP_anticodon;WHEP-TRS;tRNA-synt_2b;HGTP_anticodon2
SSTP_0000374100.1	tRNA-synt_1c;tRNA-synt_1c_C
SSTP_0000394900.1	tRNA-synt_1;tRNA-synt_1g;Anticodon_1
SSTP_0000461100.1	tRNA-synt_2;tRNA_anti-codon
SSTP_0000603200.1	tRNA-synt_1b
SSTP_0000627200.1	tRNA-synt_1c;tRNA-synt_1c_C;tRNA_synt_1c_R1;tRNA_synt_1c_R2
SSTP_0000652400.1	tRNA-synt_2c;tRNA_SAD;DHHA1
SSTP_0000654000.1	ProRS-C_1;tRNA-synt_2b;HGTP_anticodon;WHEP-TRS
SSTP_0000657200.1	tRNA-synt_1d;DALR_1;Arg_tRNA_synt_N
SSTP_0000734350.1	tRNA-synt_2d;FDX-ACB
SSTP_0000753600.1	tRNA-synt_1;tRNA-synt_1g;Anticodon_1;tRNA-synt_1e
SSTP_0000773200.1	tRNA-synt_2b
SSTP_0000787100.1	tRNA-synt_2b;Seryl_tRNA_N
SSTP_0000803500.1	tRNA-synt_1;tRNA-synt_1g;Anticodon_1
SSTP_0000900000.1	tRNA-synt_2;tRNA_anti-codon
SSTP_0000924200.1	tRNA-synt_1;Anticodon_1;tRNA-synt_1_2;tRNA-synt_1g
SSTP_0000927100.1	tRNA-synt_2
SSTP_0000944800.1	tRNA-synt_2c

SSTP_0000978500.1	tRNA-synt_1b
SSTP_0001174200.1	tRNA-synt_1e;tRNA-synt_1c;tRNA-synt_1g
SSTP_0001180000.1	tRNA-synt_1b
SSTP_0001200800.1	tRNA-synt_1g;tRNA_bind;tRNA-synt_1;Anticodon_1
SSTP_0001219100.1	tRNA-synt_2b
SSTP_0001221300.1	tRNA-synt_1g;tRNA-synt_1;tRNA-synt_1e
SSTP_0001261600.1	tRNA-synt_1b
TASK_0000070501-mRNA-1	tRNA-synt_His;HGTP_anticodon
TASK_0000081301-mRNA-1	tRNA-synt_1d
TASK_0000158301-mRNA-1	tRNA-synt_1d;DALR_1;Arg_tRNA_synt_N
TASK_0000175501-mRNA-1	tRNA-synt_1g;zf-DHHC;Ank_2;Ank_4;Ank_3;Ank_5;Ank;Anticodon_1;tRNA-synt_1
TASK_0000278001-mRNA-1	tRNA-synt_2b;Seryl_tRNA_N
TASK_0000315801-mRNA-1	tRNA-synt_1c
TASK_0000321701-mRNA-1	tRNA-synt_1;Glyco_hydro_85;Anticodon_1;Ribosomal_L17;tRNA-synt_1g
TASK_0000364801-mRNA-1	tRNA-synt_2c
TASK_0000415101-mRNA-1	tRNA-synt_1b;ANAPC4;ANAPC4_WD40
TASK_0000426301-mRNA-1	tRNA-synt_1c;tRNA-synt_1c_C;tRNA_synt_1c_R2
TASK_0000455501-mRNA-1	tRNA-synt_2b;HGTP_anticodon;TGS;tRNA_SAD
TASK_0000469601-mRNA-1	tRNA-synt_1;tRNA-synt_1g;Anticodon_1
TASK_0000472401-mRNA-1	tRNA-synt_2;tRNA_anti-codon;tRNA-synt_2d
TASK_0000488901-mRNA-1	tRNA-synt_1;Anticodon_1;tRNA-synt_1g;tRNA-synt_1_2
TASK_0000511501-mRNA-1	tRNA-synt_2b
TASK_0000532601-mRNA-1	tRNA-synt_2d
TASK_0000532801-mRNA-1	tRNA-synt_1b;tRNA_bind
TASK_0000535501-mRNA-1	tRNA-synt_1e;tRNA-synt_1g
TASK_0000548301-mRNA-1	tRNA-synt_2
TASK_0000568301-mRNA-1	tRNA-synt_2;tRNA_anti-codon
TASK_0000685401-mRNA-1	tRNA-synt_2;tRNA_anti-codon
TASK_0000695801-mRNA-1	TPR_11;TPR_8;TPR_2;TPR_1;TPR_17;tRNA-synt_2b;TPR_14;TPR_19
TASK_0000718401-mRNA-1	tRNA-synt_1;Anticodon_1;tRNA-synt_1g
TASK_0000729501-mRNA-1	tRNA-synt_2c;tRNA_SAD;DHHA1
TASK_0000758701-mRNA-1	tRNA-synt_1c
TASK_0000788501-mRNA-1	HGTP_anticodon;tRNA-synt_2b;WHEP-TRS
TASK_0000831801-mRNA-1	tRNA-synt_1;tRNA-synt_1g;Anticodon_1;tRNA-synt_1_2
TASK_0000864801-mRNA-1	tRNA-synt_1b
TASK_0000870001-mRNA-1	tRNA-synt_1;tRNA-synt_1g;Anticodon_1;tRNA-synt_1e
TASK_0000898701-mRNA-1	tRNA-synt_2;tRNA_anti-codon
TASK_0000902501-mRNA-1	tRNA-synt_1g;tRNA-synt_1
TASK_0000956901-mRNA-1	tRNA-synt_2d;FDX-ACB
TSAs00001g00232m00001	tRNA-synt_1c;tRNA-synt_1c_C
TSAs00001g00308m00001	tRNA-synt_2b
TSAs00003g01004m00001	tRNA-synt_1c
TSAs00003g01010m00001	tRNA-synt_2;tRNA_anti-codon
TSAs00003g01100m00001	DUF4147;tRNA-synt_1b;MOFRL
TSAs00006g01626m00001	tRNA-synt_2;tRNA_anti-codon
TSAs00006g01758m00001	tRNA-synt_1e;tRNA-synt_1g

TSAs00010g02399m00001	tRNA-synt_1;Glyco_hydro_85;Anticodon_1;Ribosomal_L17
TSAs00010g02417m00001	tRNA-synt_2c;tRNA_SAD;DHHA1
TSAs00010g02441m00001	tRNA-synt_2c;tRNA_SAD
TSAs00011g02653m00001	tRNA-synt_2;tRNA_anti-codon;tRNA-synt_2d
TSAs00013g02841m00001	tRNA-synt_1g;zf-DHHC;Ank_2;Ank_4;Ank_3;Ank_5;Anticodon_1;tRNA-synt_1
TSAs00014g02967m00001	tRNA-synt_1;tRNA-synt_1g;Anticodon_1
TSAs00015g03193m00001	tRNA-synt_His;HGTP_anticodon
TSAs00019g03660m00001	tRNA-synt_2
TSAs00020g03757m00001	tRNA-synt_1
TSAs00024g04081m00001	tRNA-synt_2d;FDX-ACB
TSAs00032g04889m00001	tRNA-synt_2b;HGTP_anticodon;TGS;tRNA_SAD
TSAs00039g05536m00001	tRNA-synt_1d;PaaSYMP
TSAs00045g06038m00001	tRNA-synt_2b
TSAs00052g06539m00001	tRNA-synt_1;tRNA-synt_1g;Anticodon_1;tRNA-synt_1e
TSAs00054g06661m00001	tRNA-synt_1g;tRNA-synt_1
TSAs00060g07040m00001	Arf;tRNA-synt_1b
TSAs00065g07297m00001	HGTP_anticodon;tRNA-synt_2b;WHEP-TRS
TSAs00067g07399m00001	tRNA-synt_2b;Seryl_tRNA_N
TSAs00067g07402m00001	tRNA-synt_2b;Seryl_tRNA_N
TSAs00070g07595m00001	tRNA-synt_1b;tRNA_bind
TSAs00070g07597m00001	tRNA-synt_2d
TSAs00071g07636m00001	tRNA-synt_1;Anticodon_1;tRNA-synt_1g
TSAs00074g07734m00001	tRNA-synt_1;Anticodon_1;tRNA-synt_1g;tRNA-synt_1_2
TSAs00090g08306m00001	tRNA-synt_1c;tRNA-synt_1c_C;HGTP_anticodon;ProRS-C_1;tRNA-synt_2b
TSAs00092g08357m00001	tRNA-synt_1b;ANAPC4;ANAPC4_WD40
TSAs00116g08895m00001	tRNA-synt_1d;PI3_PI4_kinase;DALR_1;Arg_tRNA_synt_N
TSAs00116g08897m00001	tRNA-synt_2;tRNA_anti-codon
TsM_000644900	tRNA-synt_1g
TsM_000555300	tRNA-synt_2;tRNA_anti-codon
TsM_000244400	tRNA-synt_2c;tRNA_SAD
TsM_000721100	tRNA-synt_2
TsM_000521500	tRNA-synt_2b
TsM_001155800	tRNA-synt_1e;tRNA-synt_1g
TsM_000029200	tRNA-synt_1b
TsM_000648300	tRNA-synt_2d;FDX-ACB
TsM_001063400	tRNA-synt_His;HGTP_anticodon
TsM_000839800	tRNA-synt_1b
TsM_000698100	tRNA-synt_2c;tRNA_SAD;DHHA1
TsM_000536400	tRNA-synt_1;Anticodon_1;tRNA-synt_1g;tRNA-synt_1_2
TsM_000773100	tRNA-synt_1;tRNA-synt_1g;Anticodon_1
TsM_000809000	tRNA-synt_2;tRNA_anti-codon
TsM_000192200	tRNA-synt_2b;HGTP_anticodon;TGS;tRNA_SAD
TsM_000996100	tRNA-synt_1;Anticodon_1;tRNA-synt_1g
TsM_000542800	tRNA-synt_1;tRNA-synt_1g;Anticodon_1;tRNA-synt_1e
TsM_001085300	tRNA-synt_1c;tRNA-synt_1c_C;HGTP_anticodon;ProRS-C_1;tRNA-synt_2b
TsM_000773500	tRNA-synt_1g;tRNA-synt_1

TsM_000330700	tRNA-synt_1c
TsM_000620600	tRNA-synt_1c;tRNA-synt_1c_C;tRNA_synt_1c_R2
TsM_000887200	tRNA-synt_1b
TsM_000846600	tRNA-synt_1d;DALR_1
TsM_000729400	HGTP_anticodon;tRNA-synt_2b;WHEP-TRS
TsM_001228300	tRNA-synt_1b;tRNA_bind
TsM_001034600	tRNA-synt_1;Anticodon_1;tRNA-synt_1g
TsM_000900500	tRNA-synt_2;tRNA_anti-codon;tRNA-synt_2d
TsM_001077300	tRNA-synt_1;tRNA-synt_1g;Anticodon_1
TsM_000665500	tRNA-synt_2;tRNA_anti-codon;HlyIII
TsM_000828100	tRNA-synt_2d
TsM_001071600	tRNA-synt_1g
TsM_001029600	tRNA-synt_1d;DALR_1;Arg_tRNA_synt_N
TsM_000894900	tRNA-synt_2b
EFV58402	tRNA-synt_1d;Arg_tRNA_synt_N;DALR_1
EFV58317	tRNA-synt_1g;tRNA_bind;tRNA-synt_1;Anticodon_1
EFV58263	tRNA-synt_His;HGTP_anticodon;WHEP-TRS
EFV58184	tRNA-synt_1;tRNA-synt_1g;Anticodon_1;tRNA-synt_1e
EFV57903	tRNA-synt_2b;HGTP_anticodon
EFV57522	HGTP_anticodon;tRNA-synt_2b
EFV61250	tRNA-synt_2b;Seryl_tRNA_N
EFV60730	TsM_001029600
EFV55995	tRNA-synt_1c
EFV55653	tRNA-synt_1;Anticodon_1;tRNA-synt_1g
EFV51304	tRNA-synt_1b
EFV50681	tRNA-synt_2b
EFV56401	tRNA-synt_2;tRNA_anti-codon
EFV59837	tRNA-synt_1;Anticodon_1;tRNA-synt_1_2;tRNA-synt_1g
EFV59666	tRNA-synt_1g;His_Phos_2;tRNA-synt_1
EFV59613	tRNA-synt_2;tRNA_anti-codon;tRNA-synt_2d
EFV62542	tRNA-synt_2
EFV54413	tRNA-synt_1;tRNA-synt_1g;Anticodon_1;tRNA-synt_1e
EFV54261	tRNA-synt_2;tRNA_anti-codon;tRNA-synt_2d
EFV55105	tRNA-synt_1c;tRNA_synt_1c_R2;tRNA_synt_1c_R1;;tRNA-synt_1c_C
EFV53828	tRNA-synt_1;tRNA-synt_1g;Anticodon_1
EFV61767	tRNA-synt_1c;WHEP-TRS;tRNA-synt_1c_C;ProRS-C_1
	;tRNA-synt_2b;HGTP_anticodon
EFV61632	tRNA-synt_1b;tRNA_bind
EFV54757	tRNA-synt_1b;ThiF;E2_bind
EFV54739	tRNA-synt_1e;tRNA-synt_1g;tRNA-synt_1
EFV54681	tRNA-synt_1;Glyco_transf_22;Anticodon_1;tRNA-synt_1g
EFV51596	tRNA-synt_2c;tRNA_SAD
EFV50282	tRNA-synt_2d;FDX-ACB;Methyltransf_11
EFV52543	tRNA-synt_2c;UcrQ
EFV53336	tRNA-synt_1d;Ribosomal_L22;DALR_1
EFV59868	Aa_trans;tRNA-synt_2b;HGTP_anticodon;tRNA_SAD;TGS

EFV49888	tRNA-synt_2b;HGTP_anticodon;tRNA_SAD;TGS
EFV49577	tRNA-synt_1
EFV49576	tRNA-synt_1
EFV49113	tRNA-synt_1c;tRNA-synt_1c_C;WHEP-TRS
EFV48446	tRNA-synt_1;tRNA-synt_1g
EFV48184	tRNA-synt_2b
TTRE_0000013401-mRNA-1	tRNA-synt_2;tRNA_anti-codon
TTRE_0000029701-mRNA-1	tRNA-synt_1;Glyco_transf_22;Anticodon_1;RNase_PH;tRNA-synt_1g
TTRE_0000046401-mRNA-1	tRNA-synt_2b;HGTP_anticodon;TGS;tRNA_SAD
TTRE_0000049201-mRNA-1	tRNA-synt_1c
TTRE_0000058801-mRNA-1	Evr1_Alr;tRNA-synt_2d;FDX-ACB;Thioredoxin;Methyltransf_11;Methyltransf_23
	;Methyltransf_31;Methyltransf_12;Methyltransf_25;Ubie_methyltran
TTRE_0000143501-mRNA-1	tRNA-synt_1c;tRNA_synt_1c_R1;tRNA-synt_1c_C;tRNA_synt_1c_R2
TTRE_0000200701-mRNA-1	tRNA-synt_1
TTRE_0000228101-mRNA-1	tRNA-synt_1;Anticodon_1;tRNA-synt_1g
TTRE_0000229401-mRNA-1	tRNA-synt_2b;Seryl_tRNA_N
TTRE_0000233201-mRNA-1	tRNA-synt_2;tRNA_anti-codon;tRNA-synt_2d
TTRE_0000258501-mRNA-1	tRNA-synt_1;Anticodon_1;tRNA-synt_1_2;tRNA-synt_1g
TTRE_0000273301-mRNA-1	tRNA-synt_1b;tRNA_bind
TTRE_0000292901-mRNA-1	tRNA-synt_1g;Methyltransf_11;tRNA-synt_1;tRNA-synt_1e;
	Methyltransf_31;Anticodon_1
TTRE_0000305301-mRNA-1	tRNA-synt_2d
TTRE_0000394101-mRNA-1	tRNA-synt_2;tRNA_anti-codon;tRNA-synt_2d
TTRE_0000413801-mRNA-1	tRNA-synt_2;tRNA_anti-codon
TTRE_0000416201-mRNA-1	tRNA-synt_1b;ABC_tran;AAA_21;SMC_N;RLI;AAA_15;Fer4;AAA_16;AAA_22;AAA
TTRE_0000426701-mRNA-1	HMG_CoA_synt_N;tRNA-synt_1d;HMG_CoA_synt_C;DALR_1
TTRE_0000457401-mRNA-1	tRNA-synt_1b
TTRE_0000465101-mRNA-1	tRNA-synt_2b
TTRE_0000465901-mRNA-1	HGTP_anticodon;tRNA-synt_2b
TTRE_0000474501-mRNA-1	tRNA-synt_1e;zf-C2H2_jaz;tRNA-synt_1g;tRNA-synt_1
TTRE_0000478601-mRNA-1	tRNA-synt_2;tRNA_anti-codon
TTRE_0000482001-mRNA-1	tRNA-synt_2b;HGTP_anticodon
TTRE_0000525201-mRNA-1	tRNA-synt_2
TTRE_0000526901-mRNA-1	tRNA-synt_2;tRNA_anti-codon;tRNA-synt_2d
TTRE_0000569901-mRNA-1	tRNA-synt_1g;tRNA_bind;tRNA-synt_1
TTRE_0000593301-mRNA-1	tRNA-synt_1b
TTRE_0000602301-mRNA-1	tRNA-synt_2c
TTRE_0000625901-mRNA-1	tRNA-synt_1;tRNA-synt_1g;Anticodon_1
TTRE_0000642101-mRNA-1	tRNA-synt_2c;tRNA_SAD;DHHA1
TTRE_0000691801-mRNA-1	tRNA-synt_1d;DALR_1;Arg_tRNA_synt_N;tRNA-synt_1e
TTRE_0000730701-mRNA-1	tRNA-synt_1_2;tRNA-synt_1;tRNA-synt_1g;Anticodon_1
	;tRNA-synt_1e;tRNA-synt_1f
TTRE_0000776101-mRNA-1	tRNA-synt_1;Anticodon_1;tRNA-synt_1g;
	Val_tRNA-synt_C;tRNA-synt_1_2;tRNA-synt_1e;tRNA-synt_1f
TTRE_0000783401-mRNA-1	tRNA-synt_His;HGTP_anticodon
TTRE_0000806801-mRNA-1	tRNA-synt_2c;tRNA_SAD;DHHA1

TTRE_0000811301-mRNA-1	tRNA-synt_His;tRNA-synt_2b;TPR_21;HGTP_anticodon
TTRE_0000814101-mRNA-1	tRNA-synt_2b;tRNA_edit;HGTP_anticodon
TTRE_0000820201-mRNA-1	ProRS-C_1;tRNA-synt_2b;HGTP_anticodon
TTRE_0000824901-mRNA-1	tRNA-synt_1e;tRNA-synt_1g;DALR_2;tRNA-synt_1f;tRNA-synt_1
TTRE_0000825101-mRNA-1	tRNA-synt_1c
TTRE_0000826101-mRNA-1	tRNA-synt_1d;DALR_1;Arg_tRNA_synt_N
TTRE_0000828401-mRNA-1	tRNA-synt_2c;tRNA_SAD;DHHA1
TTRE_0000847801-mRNA-1	Anticodon_1;tRNA-synt_1g;tRNA-synt_1e;tRNA-synt_1
TTRE_0000861901-mRNA-1	tRNA-synt_1c;tRNA-synt_1c_C
TTRE_0000864901-mRNA-1	tRNA-synt_1;Anticodon_1;tRNA-synt_1g;Val_tRNA-synt_C;tRNA-synt_1_2
TTRE_0000885801-mRNA-1	tRNA-synt_1c;tRNA-synt_1c_C
TTRE_0000892301-mRNA-1	tRNA-synt_1b
TTRE_0000915101-mRNA-1	tRNA-synt_1;tRNA-synt_1g
TTRE_0000926601-mRNA-1	tRNA-synt_2b;Ribosomal_L20;IF3_C;IF3_N;HGTP_anticodon; Ribosomal_L35p;tRNA_SAD;TGS
TTRE_0000926701-mRNA-1	tRNA-synt_2d;Phe_tRNA-synt_N
TTRE_0000966201-mRNA-1	tRNA-synt_1c
maker-PairedContig_1194-snap-gene-0.2-mRNA-1	tRNA-synt_2;tRNA_anti-codon
maker-PairedContig_1198-snap-gene-4.15-mRNA-1	Nt_Gln_amidase;tRNA-synt_1b
maker-PairedContig_1326-snap-gene-0.4-mRNA-1	tRNA-synt_1c
maker-PairedContig_1338-snap-gene-1.11-mRNA-1	tRNA-synt_1b
maker-PairedContig_1459-snap-gene-0.11-mRNA-1	tRNA-synt_2;tRNA_anti-codon
maker-PairedContig_151-snap-gene-0.2-mRNA-1	ProRS-C_1;tRNA-synt_2b;HGTP_anticodon
maker-PairedContig_1555-snap-gene-0.5-mRNA-1	tRNA-synt_2d;FDX-ACB
maker-PairedContig_1555-snap-gene-0.6-mRNA-1	tRNA-synt_1;Anticodon_1;tRNA-synt_1g
maker-PairedContig_16-snap-gene-0.3-mRNA-1	tRNA-synt_1;Anticodon_1;tRNA-synt_1g
maker-PairedContig_1625-snap-gene-2.36-mRNA-1	tRNA-synt_1d;DALR_1
maker-PairedContig_1679-snap-gene-0.25-mRNA-1	tRNA-synt_2;tRNA_anti-codon
maker-PairedContig_1679-snap-gene-0.28-mRNA-1	tRNA-synt_2d;tRNA-synt_2
maker-PairedContig_1765-snap-gene-0.5-mRNA-1	tRNA-synt_1c;tRNA_synt_1c_R1;tRNA-synt_1c_C;tRNA_synt_1c_R2
maker-PairedContig_1808-snap-gene-0.20-mRNA-1	tRNA-synt_1g;tRNA-synt_1;tRNA-synt_1e
maker-PairedContig_201-snap-gene-0.9-mRNA-1	tRNA-synt_1c;WHEP-TRS;tRNA-synt_1c_C;tRNA-synt_2b
maker-PairedContig_2047-snap-gene-1.25-mRNA-1	HGTP_anticodon;WHEP-TRS;tRNA-synt_2b
maker-PairedContig_206-snap-gene-6.13-mRNA-1	tRNA-synt_1b
maker-PairedContig_2062-snap-gene-1.12-mRNA-1	tRNA-synt_2
maker-PairedContig_2091-snap-gene-7.12-mRNA-1	tRNA-synt_2c;tRNA_SAD;DHHA1
maker-PairedContig_2091-snap-gene-8.15-mRNA-1	tRNA-synt_1;tRNA-synt_1g
maker-PairedContig_3141-snap-gene-0.5-mRNA-1	tRNA-synt_1;Anticodon_1;tRNA-synt_1g;tRNA-synt_1e
maker-PairedContig_3580-snap-gene-0.4-mRNA-1	tRNA-synt_1b
maker-PairedContig_4131-snap-gene-0.5-mRNA-1	tRNA-synt_2
maker-PairedContig_432-snap-gene-1.12-mRNA-1	tRNA-synt_1;Anticodon_1;tRNA-synt_1g
maker-PairedContig_4489-snap-gene-0.16-mRNA-1	tRNA-synt_2c;tRNA_SAD
maker-PairedContig_467-snap-gene-0.15-mRNA-1	tRNA-synt_1g;tRNA_bind;tRNA-synt_1
maker-PairedContig_4689-snap-gene-1.14-mRNA-1	tRNA-synt_2b
maker-PairedContig_5379-snap-gene-2.26-mRNA-1	tRNA-synt_His;HGTP_anticodon;HGTP_anticodon2
maker-PairedContig_5864-snap-gene-0.9-mRNA-1	tRNA-synt_2b;HGTP_anticodon

maker-PairedContig_5969-snap-gene-0.12-mRNA-1	tRNA-synt_1g
maker-PairedContig_650-snap-gene-1.19-mRNA-1	tRNA-synt_1;tRNA-synt_1g
maker-PairedContig_979-snap-gene-0.3-mRNA-1	tRNA-synt_1e;tRNA-synt_1g
snap_masked-PairedContig_1070-processed-gene-0.0-mRNA-1	tRNA-synt_1;Chromo
snap_masked-PairedContig_3489-processed-gene-0.1-mRNA-1	tRNA-synt_1d;Arg_tRNA_synt_N;DALR_1
snap_masked-PairedContig_4361-processed-gene-0.0-mRNA-1	RRM_1;RRM_6;tRNA-synt_1c;RRM_5
snap_masked-PairedContig_5411-processed-gene-8.11-mRNA-1	tRNA-synt_2b;Seryl_tRNA_N
snap_masked-PairedContig_6294-processed-gene-1.0-mRNA-1	tRNA-synt_2b;HGTP_anticodon;TGS;tRNA_SAD
