# Supplementary figures and images for: Genomic analyses of aminoacyl tRNA synthetases from human-infecting helminths
Source: BMC Genomics. 2019 May 2;20:333. doi: 10.1186/s12864-019-5679-0 (PMC6498573; doi:10.1186/s12864-019-5679-0)

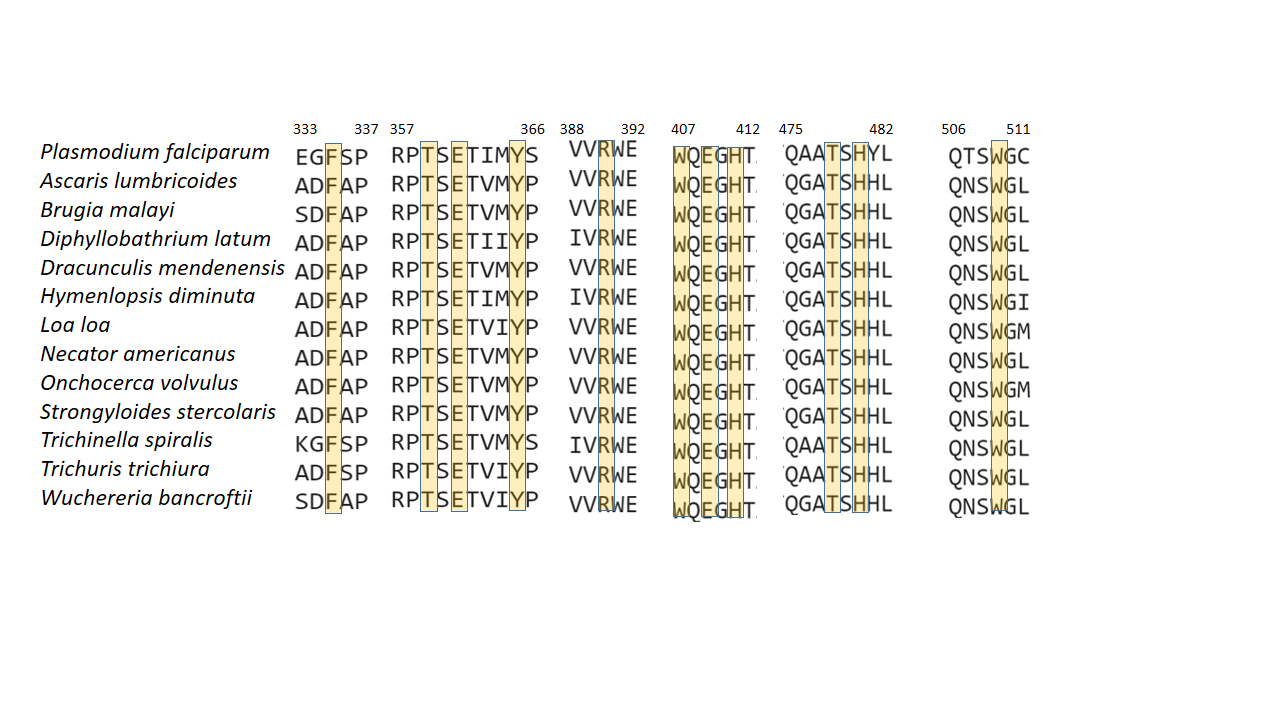


A


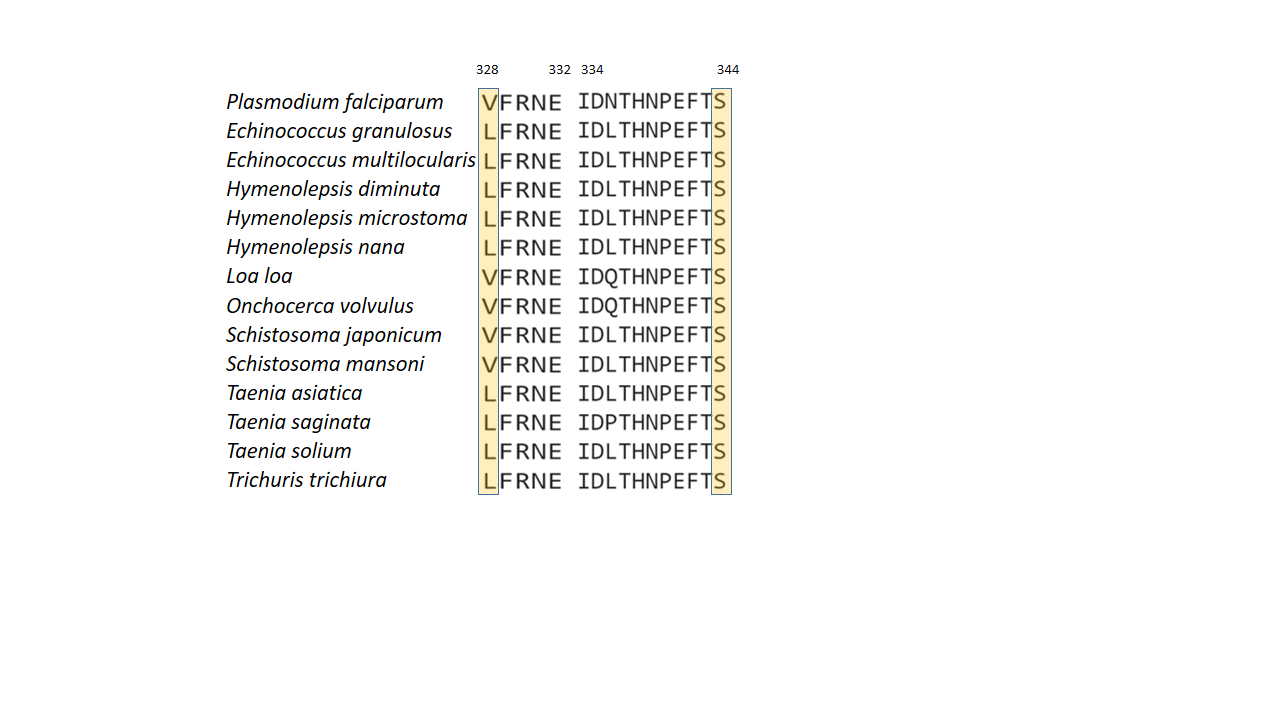


C

B


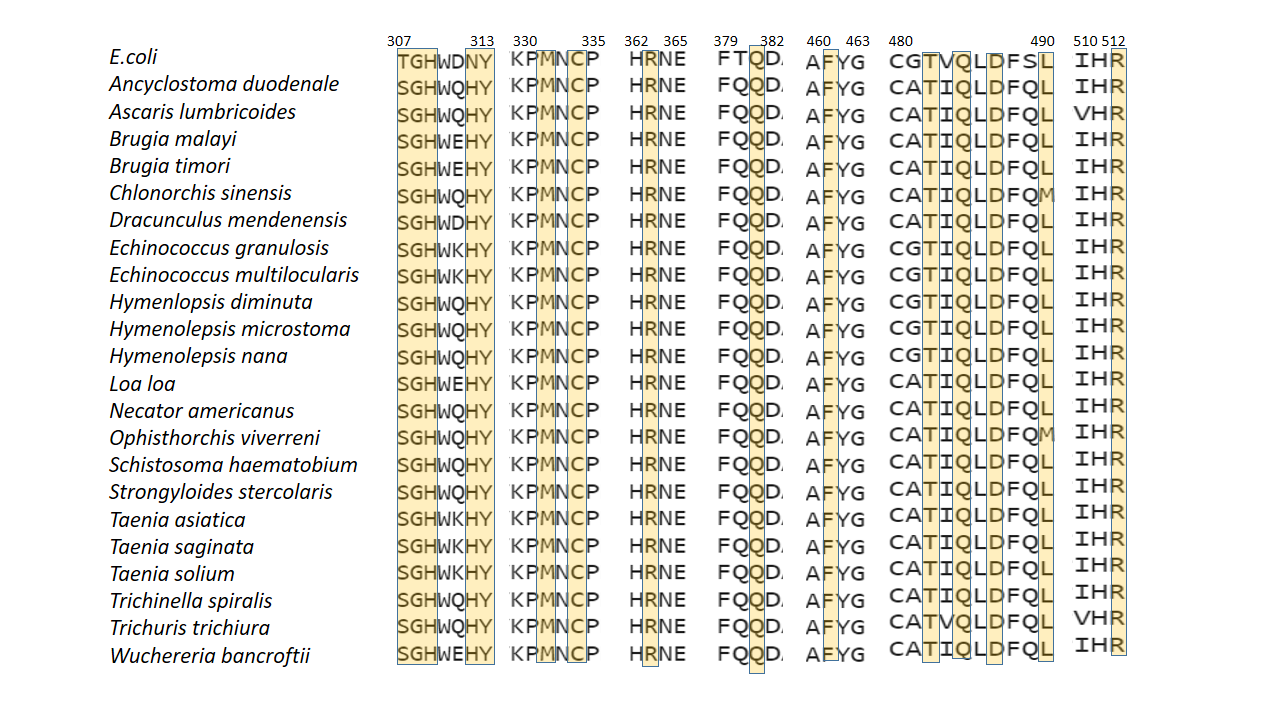


D


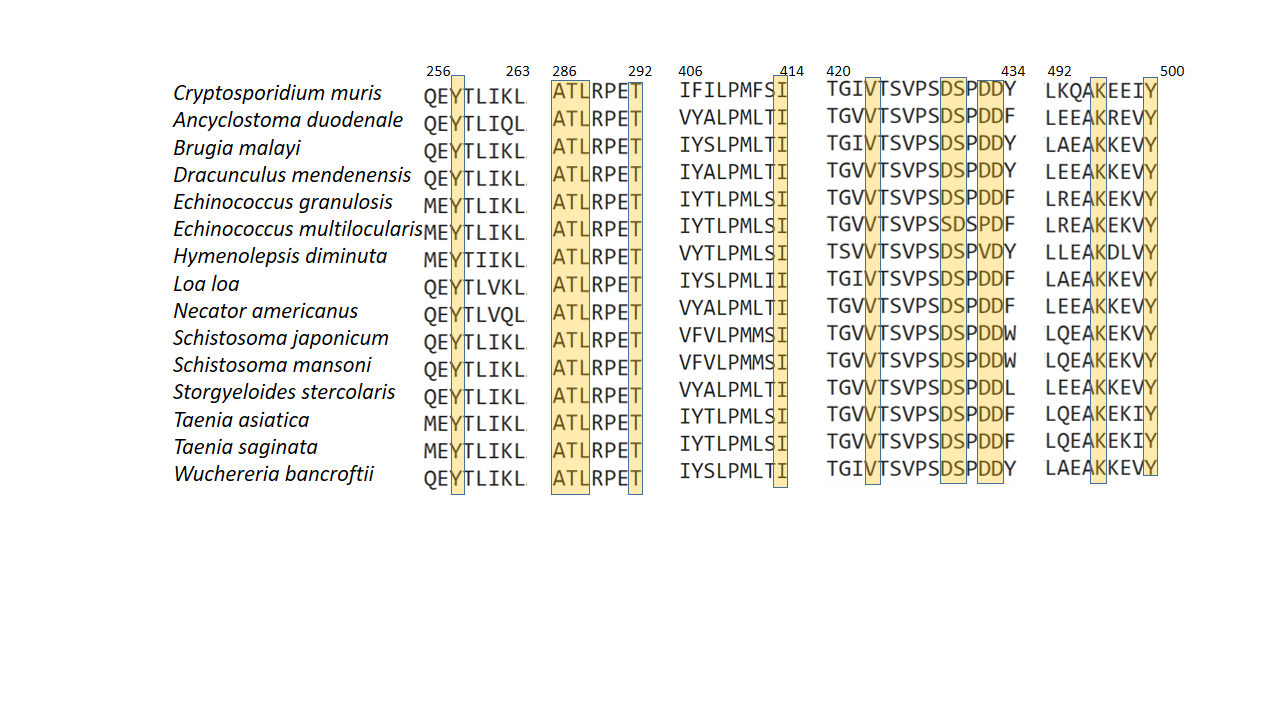

Supplement: Supplementary file 3 — Figure S1. Multiple sequence alignment (MSA) of aaRSs 1) PRS of Plasmodium falciparum with PRSs of all studied human-infecting helminths showing conservation in HF binding residues, 2) CLD binding residues in Plasmodium falciparum aligned with KRSs of helminths, 3) binding residues in TRS of Cryptosporidium muris aligned with TRSs of helminths, 4) Benzoborale binding residues in LRS of E.coli aligned with LRSs of helminths. (DOCX 1888 kb) [file 12864_2019_5679_MOESM3_ESM.docx]
